# Accelerated Discovery of Topological Conductors for Nanoscale Interconnects

**DOI:** 10.1002/advs.202520535

**Published:** 2026-01-14

**Authors:** Alexander C. Tyner, William Rogers, Po‐Hsin Shih, Yi‐Hsin Tu, Gengchiau Liang, Hsin Lin, Ching‐Tzu Chen, James M. Rondinelli

**Affiliations:** ^1^ NORDITA, KTH Royal Institute of Technology and Stockholm University Stockholm Sweden; ^2^ Department of Physics University of Connecticut Storrs CT USA; ^3^ Graduate Program in Applied Physics Northwestern University Evanston IL USA; ^4^ IBM Thomas J. Watson Research Center Yorktown Heights NY USA; ^5^ Industry Academia Innovation School National Yang Ming Chiao Tung University Hsinchu Taiwan; ^6^ Institute of Physics Academia Sinica Taipei Taiwan; ^7^ Department of Materials Science and Engineering Northwestern University Evanston IL USA

**Keywords:** DFT calculations, interconnects, machine learning, nanowire transport, topological conductors

## Abstract

The sharp increase in resistivity of copper interconnects at ultra‐scaled dimensions threatens the continued miniaturization of integrated circuits. Topological semimetals (TSMs) with gapless surface states (Fermi arcs) provide conduction channels resistant to localization. Here we develop an efficient computational framework to quantify 0 K surface‐state transmission in nanowires derived from Wannier tight‐binding models of topological conductors that faithfully reproduce relativistic density functional theory results. Sparse matrix techniques enable scalable simulations incorporating disorder and surface roughness, allowing systematic materials screening across sizes, chemical potentials, and transport directions. A dataset of 3000 surface transmission values reveals TiS, ${\mathrm{ZrB}}_{2}$, MoC, WC, and nitrides AN where A=(Mo,Ta,W) as candidates with conductance matching or exceeding copper and benchmark TSMs NbAs and NbP. This dataset further supports machine learning models for rapid interconnect compound identification. Our results highlight the promise of topological conductors in overcoming copper's scaling limits and provide a roadmap for data‐driven discovery of next‐generation interconnects.

## Graphical abstract

Copper interconnects exhibit a sharp increase in resistivity at ultra‐scaled dimensions, threatening continued miniaturization of integrated circuits. The gapless surface states of topological semimetals provide conduction channels resistant to localization. We develop an efficient computational framework to generate a dataset of 3000 zero‐Kelvin surface transmission values for topological semimetals. This dataset reveals optimal interconnect candidates and supports machine learning models for future discovery workflows.

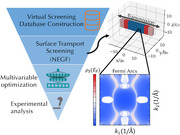

## 1 Introduction

As the dimensions of logic and memory components in modern integrated circuits continue to shrink, copper interconnects between devices must also be proportionally scaled down to maintain density. At nanoscale dimensions, the electrical resistivity of copper increases sharply, leading to higher energy consumption and RC delays. This effect becomes significant when the critical dimension (CD) of copper interconnects falls below the electron mean free path, λ=39 nm [1, 2, 3, 4, 5], where surface and grain boundary scattering dominate electron transport. Degradation in performance results in slower, less energy‐efficient computation, underscoring the need for alternative materials to replace copper. Topological semimetals (TSMs) [6, 7, 8, 9, 10] and topological metals (TMs) [11, 12, 13] have emerged as a promising materials class to replace copper [2, 14, 15, 16], owing to their topologically protected surface states that enable robust, low‐resistance conducting channels (see Discussion V, Supporting Information). The TSM/TM classification is broad, encompassing Weyl [10, 17], multifold [18, 19], triple point [11], and nodal line [20, 21, 22, 23] systems among others. Common among each of these classes are nodal points or lines in the Brilloun zone where bands cross near the Fermi energy. In the case of a nodal point semimetal, the band crossing locations act as monopoles and antimonopoles of Berry curvature. Importantly, the non‐trivial Berry flux gives rise to chiral edge states, commonly known as Fermi arcs states, between the projection of the nodal points on the surface. Prior computational works have investigated the utility of the Fermi arcs states as conduction channels in the ultra‐thin limit for Weyl semimetal NbAs [24, 25] and multifold fermion semimetal CoSi [26]. These works showed that conduction is increasingly dominated by Fermi arcs as the sample thickness is decreased, leading to a decreasing resistance‐area (RA) product with decreasing thickness.

Although isolated case studies have demonstrated the promise of topological conductors as next‐generation interconnects, they have also shown that not all are created equal in this context. A broader and systematic exploration is needed to fully assess and optimize their potential. Recent high‐throughout screenings of inorganic materials for nontrivial topology, such as those compiled in the topological quantum chemistry database [27, 28], have significantly expanded the catalog of known TSMs, providing a reasonable starting point for targeted screening efforts aimed at identifying TSMs with favorable surface transport properties for interconnect applications. Nevertheless, such a screening is yet to be undertaken due to the computational demands associated with determination of nanowire surface conductance via first‐principles. This expense arises due to the need to consider nanowire geometries of sufficient size to project the conductance performance at the relevant length scale for the interconnect technologies. Such nanowires can contain hundreds of atoms, creating a bottleneck not only for convergence within density functional theory (DFT), but particularly for computation of transmission via the non‐equilibrium Green's function (NEGF) formalism [29]. To make matters worse, these nanowire computations must be performed multiple times, considering a variety of surfaces, transmission directions, and disorder.

We reduce this computational expense by constructing nanowires from Wannier tight‐binding models of candidate TSMs to obtain a high‐fidelity representation of the electronic structure of each compound using automated and efficient schemes. Once constructed, we use the hopping parameters provided by the Wannier tight‐binding to construct nanowire geometries, which are represented as sparse matrices for which computationally advantageous methods can be used to tackle large system sizes efficiently. Furthermore, the computational efficiency of this protocol allows us to investigate the effects of disorder and surface roughness on the bulk and surface transmission. These computations are performed in the  0 K limit, however we expect the results to be relevant for down‐selection of optimal room‐temperature interconnects due to the low electron–phonon coupling computed for topological surface states in prior works [31, 32, 33, 34]. In particular, we expect finite temperature effects to have greater impact on the relative ranking of interconnect candidates within distinct topological class when compared to candidates within the same topological class due to prior work demonstrating the effect of surface state geometry on the electron‐phonon scattering phase space [34]. Only idealized straight‐line Fermi arcs are shown to have a vanishing electron‐phonon scattering phase space [34]. A finite curvature is shown to increase the phase space with a dramatic increase in the scattering phase space for surface states forming an arc with an angle greater than $180^{\circ }$. This distinction separates the Fermi arc states of TSMs and the Dirac cone surface states of topological insulators (TIs).

Our analysis reveals TiS [35, 36], ${\mathrm{ZrB}}_{2}$ [20, 37], MoC, WC and nitrides AN [11] where A=(Mo,Ta,W) as previously known but unidentified interconnect candidates, displaying surface transmission competitive with TSMs that have been studied in depth previously. For interconnect candidates displaying large surface transmission, we further investigate the resistivity scaling coefficient, $\rho_{0}\lambda$, where $\rho_{0}$ is the bulk resistivity and λ is the mean‐free path. This first‐principles accessible quantity [1, 38], provides complementary information about bulk transmission, important for optimization of the device at realistic dimensions. Last, we discuss properties such as surface energy and electro‐migration barrier, which require optimization to ensure manufacturability and reliability during device operation.

## 2 Computational Workflow

### 2.1 Candidate Selection

**FIGURE 1:**
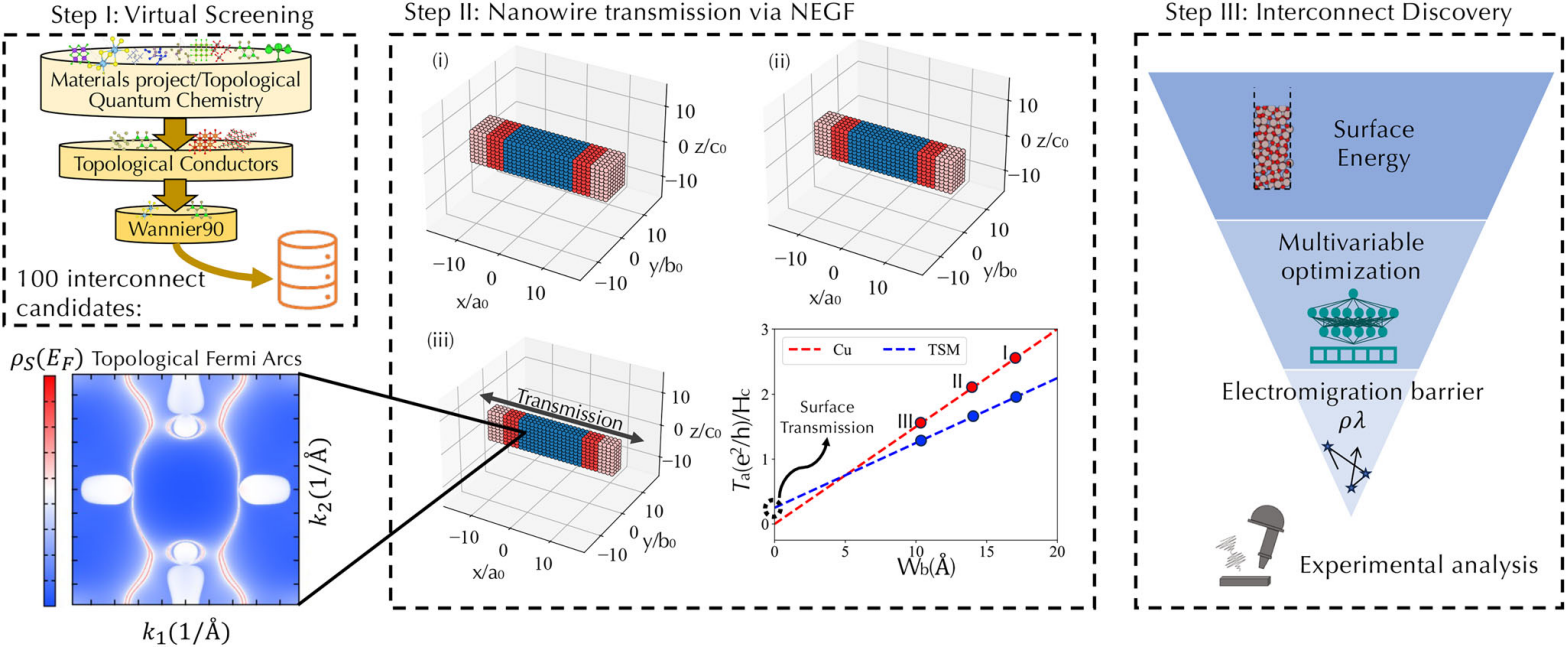
Accelerated topological semimetal interconnect discovery workflow. Step I (top left) filtering material candidates from existing databases of thermodynamic and topological properties. The current search is limited to binary and selected ternary and elemental conductors, considering those found on the DFT convex hull within the Materials Project [30] or for which examples of experimental synthesis are available. After filtering candidates we perform density‐functional theory calculations and construct a Wannier tight‐binding model for each compound. Step II (center): Tight‐binding models are used to construct nanowires and transmission is computed via the non‐equilibrium Greens function (NEGF) formalism as a function of thickness along an axis perpendicular to the transmission direction as indicated in (i). In the geometry shown, transmission is computed along [100], $T_{a}$, arising from the Fermi arcs on the (010) surface (example spectral density shown in lower left) as the transverse width of the nanowire along the [010] direction, $W_{b}$, decreases moving from (i) to (iii). (The vertical height of the nanowire along [001] is fixed.) The zero‐thickness intercept is obtained by linearly fitting the transmission as a function of the transverse width to obtain the surface transmission $T_{s}$, which is equivalent to $T_{a}\left(W_{b}\to 0\right)$, for the given geometry (bottom right, center panel). Step III (right) Higher‐fidelity simulations and targeted property evaluations are performed to optimize candidate materials against additional interconnect‐application constraints. Based on these refined criteria, the most promising candidates are identified and recommended for experimental validation.

The first stage in our workflow involves a virtual screening of publicly available inorganic structure databases (Figure 1). We limit our search primarily to binary and elemental topological conductors including a small number of experimentally realized ternaries that are computationally tractable. This constraint in structure is motivated by the high memory demands associated with the final step of our workflow—nanowire transmission calculations—which remain a computational bottleneck. The virtual screening process aims to curate a focused dataset of promising compounds for further investigation, thereby reducing the dimensionality of the candidate space [39].

In practice, candidate identification is carried out through a combination of manual searches of online databases, including the Topological Materials Database [40], Topological Materials Arsenal [41], and Materiae [42], as well literature reviews. We prioritize compounds that have either been experimentally synthesized or exhibit evidence of nontrivial bulk topology in prior computational studies. The full list of selected compounds is provided in Table S1 (Supporting Information), identified by Materials Project ID [30]. While this list may not encompass all viable topological conductors, it serves as a practical starting point for deploying our discovery workflow. For compounds that have not been experimentally realized, we require that they lie on the convex hull, which is readily accessible via the Materials Project [30] (v2023.11.1). Additionally, we limit our current screening to non‐magnetic systems, deferring the inclusion of ferromagnetic and antiferromagnetic candidates for future work. All necessary files to reproduce the data generated in this study are available at Ref. [43]. A breakdown of the space group distribution and elemental composition within the dataset is provided in Figure S1 (Supporting Information). Last, we also include current interconnect candidates ${\mathrm{PtCoO}}_{2}$ and elemental Mo, Ir, and Cu to provide a benchmark for the workflow.

### 2.2 Electronic Structure Simulations

The next stage in our computational workflow involves constructing a Wannier tight‐binding model for each compound using the conventional unit cell as the structural basis [44, 45]. We deliberately choose the conventional unit cell over the primitive unit cell to ensure compatibility with downstream nanowire transmission simulations, which are performed along the principal crystallographic axes of the conventional unit cell. This choice facilitates a direct mapping between bulk electronic structure and transport directionality, enabling consistent and physically meaningful comparisons across different materials. We perform density functional theory (DFT) computations with spin‐orbit coupling (SOC) using the Quantum Espresso software package [46, 47].

### 2.3 Nanowire Surface Transmission

We next construct a nanowire of dimension (L×W×H) conventional unit cells using the Kwant software package [29]. With this geometry, the transmission T is always simulated along L to identify contributions due to the surface perpendicular to $W_{i}$. Since we consider surface orientations for each principal axes, i=a,b,andc, we append a subscript to the dimension to denote the corresponding crystal orientation. The magnitude of individual lattice parameters are denoted $a_{0},b_{0},\mathrm{and}c_{0}$ for clarity. For example, $\left(L_{a}\times W_{b}\times H_{c}\right)$ describes the nanowire shown in the center panel of Figure 1, for which transmission along the [100] direction is due to states on the (010) surface in a laboratory frame.

To isolate the contribution of surface states to transmission, we employ a procedure in which leads are attached to the compound under investigation, followed by a series of simulations where the size of the nanowire is progressively reduced in a direction transverse to the transmission axis as described below. In the example presented in Figure 1 Step II(i), we begin with an isolated nanowire of dimensions, $\left(L_{a}\times W_{b}\times H_{c}\right)$ = (16×8×5). Leads, formed from the same real‐space tight‐binding model, and extending semi‐infinitely along the [100] direction are then attached. The setup appears as shown in Figure 1 under step II(i), where the scattering region is shown in blue, the leads are shown in light‐red, and red indicates the region in which the scattering region and leads overlap. We then compute the transmission, normalized by the height of the scattering region ($H_{c}$). For each configuration, we also vary the electron chemical potential between [−0.2eV,0.2eV] in increments of 0.1eV when computing transmission as the position of the Fermi energy $E_{F}$ may be unknown due to unintentional defects.

In the next step, we decrease the number of unit cells along $W_{b}$ and recompute the transmission for each value of the chemical potential, recognizing that as $W_{b}\to 0$ the total transmission is linear with respect $W_{b}$ as

(1) $$T\left(W_{i}\right)=T_{S}+W_{i}\;g_{\mathrm{bulk}},$$

where T is the total transmission, $T_{S}$ is the transmission due to the surface perpendicular to the i crystal axis and $W_{i}\;g_{\mathrm{bulk}}$ gives the bulk transmission as shown in the bottom right of Step II in Figure 1. Provided that the thickness $W_{b}$ is greater than the critical dimension below which quantum confinement completely gaps out the bulk states, then each time $W_{b}$ is reduced, the number of bulk conducting channels should decrease in a linear manner, while conducting channels on the surface perpendicular to $W_{b}$ remain unaffected. The zero‐thickness limit of the linear fit to $T(W_{i})$ gives the contribution from the surface states to the transmission. For additional analysis of quantum confinement effects and quality of the linear fit see Discussion IV (Supporting Information). This process is repeated for all permutations of transmission direction and surface orientation. The results are then plotted and inspected to identify optimal compounds and transmission directions.

### 2.4 High‐Fidelity Simulations

After the initial screening, materials displaying promising surface transmission are further refined by creating a Wannier tight‐binding model using hydrogenic orbitals, excluding f‐electrons if present. We ensure that the refined Wannier models precisely match the DFT band structure along the high‐symmetry path in the Brillouin zone within 1 eV of the Fermi energy and then repeat the computations of surface transmission to ensure accuracy. Detailed quantitative analysis of this approach is provided in the Discussion III (Supporting Information).

### 2.5 Machine Learning Model

We use data generated from the surface transmission workflow to train a a random forest regression model that predicts transmission across the broader chemical space of binary compounds. Each data point used to train the machine learning model corresponds to a compound labeled by its maximal surface transmission value, $T_{S}$, at the Fermi energy when considering all transmission directions and surface combinations; yielding a single data point per compound. To represent each compound, we employ Magpie descriptors [48], accessed via the MatMiner package [49], which encode a wide range of compositional and elemental properties. Additionally, we use the topogivity index [50] as a feature, which assigns a scalar value to each element, averaged across constituent atoms in a compound, with higher averages indicating a greater likelihood of nontrivial ground state topology. The feature captures elemental contributions such as spin‐orbit coupling strength and reflects the tendency of certain elements to favor topologically nontrivial phases. To identify the most important features and reduce dimensionality, we apply SHAP (SHapley Additive exPlanations) analysis using the Python SHAP library [51]. This interpretability framework allows us to extract physical insights from the trained model, which is implemented using the scikit‐learn library [52]. The capability to generate models that accurately capture nanowire transmission as a function of Fermi energy and wire direction/surface combinations is an objective for future work.

## 3 Results and Discussion

### 3.1 Maximum Surface Transmission Compounds

**FIGURE 2:**
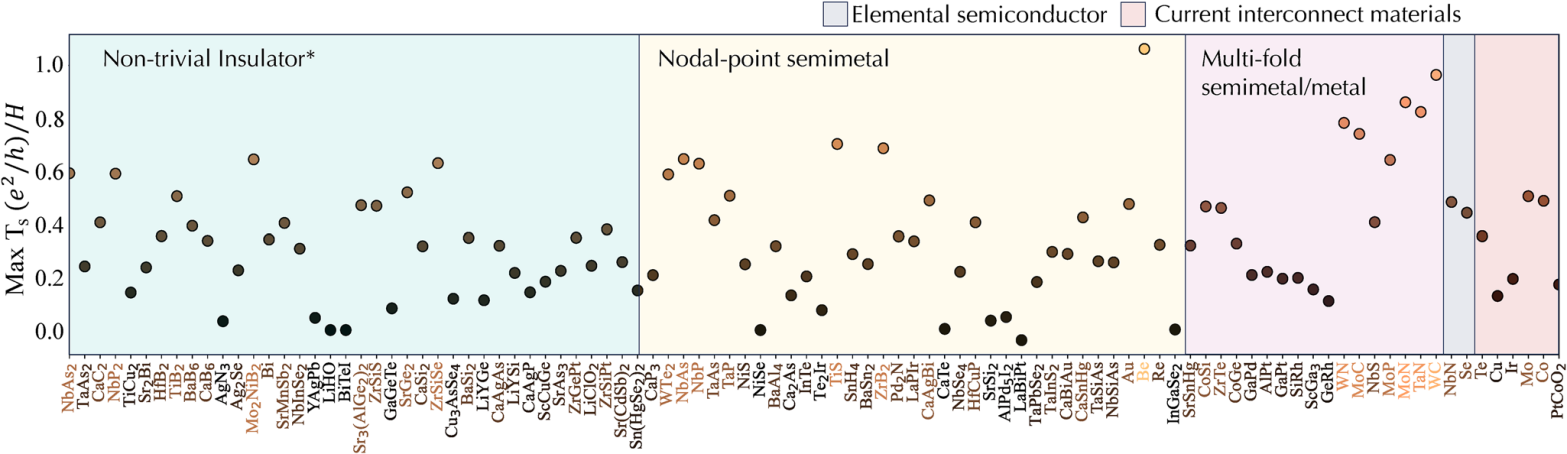
Maximum calculated nanowire surface transmission values. The maximum $T_{s}$ values obtained at the Fermi energy for the compounds screened via the process in Figure 1, Step II. Materials sorted by their topological quantum chemistry classification. The general term, nontrivial ${\mathrm{insulator}}^{*}$ is used to group materials with occupied topological bands. An asterisk is included to emphasize that these systems need not, and in most cases do not, have a direct band gap at the Fermi level.

**FIGURE 3:**
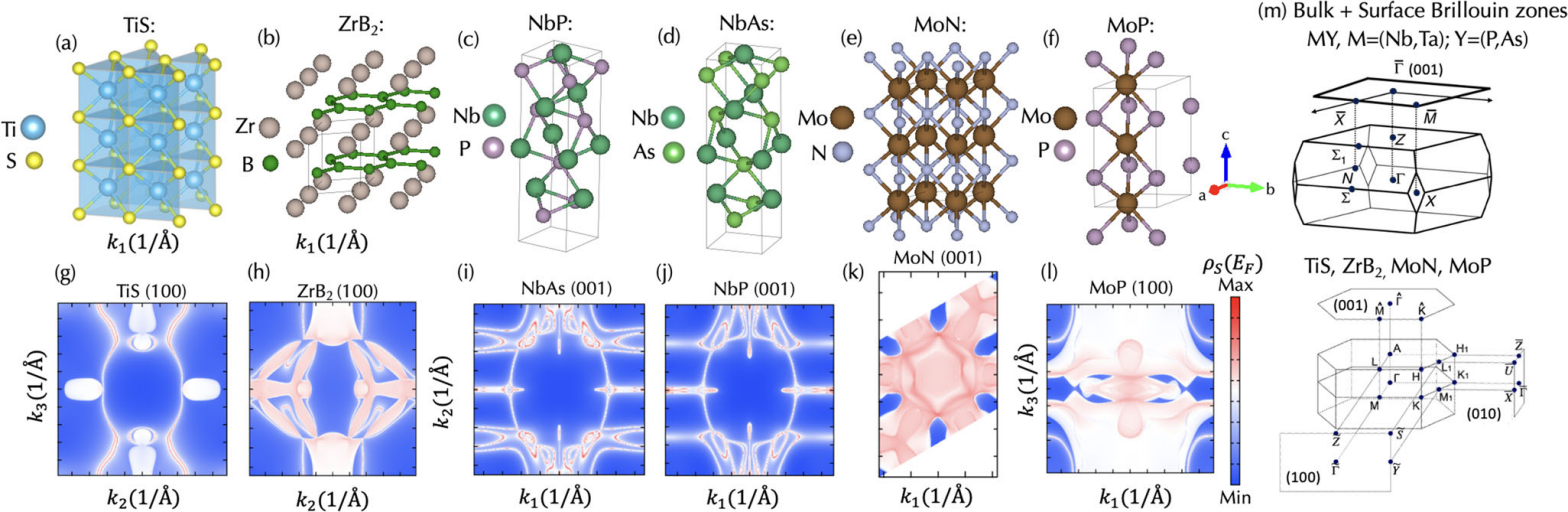
Crystal structure and spectral density dispersions for promising topological semimetals. Constant energy surface spectral density at the Fermi energy centered about $\bar{\Gamma }$ for TiS, ${\mathrm{ZrB}}_{2}$, and MoN, identified in this work, compared with topological interconnect candidates NbPn, where Pn=(P, As), and MoP reported to support enhanced surface transmission. Spectral density figures labeled by surface for which transmission is maximal. Colorbar represents the magnitude of the surface spectral density at the $E_{F}$ (red, maximal; blue, minimal).

The maximal transmission direction/surface combination at the Fermi energy for each screened binary and elemental compound is shown in Figure 2. Compounds are grouped according to their topological classification via analysis of elementary band representations [27, 53, 54, 55]. The general term nontrivial insulator is used to specify a broad group containing all systems supporting occupied bands with nontrivial topology, but following the formalism of Ref. [27], an asterisk is included to emphasize that these systems need not, and in most cases do not, have a direct band gap at the Fermi level. Additionally, we separate nodal‐point semimetals, which support crossings between two bands, from multi‐fold semimetals, which support crossings between N>2 bands at high‐symmetry locations in the Brillouin zone. From this data we immediately find that Cu, the current industry standard for interconnect applications, has minimal surface transmission. We also identify two previously known but unidentified promising interconnect candidates, TiS, ${\mathrm{ZrB}}_{2}$ (Figure 2), in addition to known monopnictide *M*Pn TSMs, where M=(Nb,Ta) and Pn=(P,As) [56, 57]. Additionally, we find that mononitrides MoN, WN, and TaN are promising candidates from the family of topological triple point metals [11] for which MoP has been identified previously as an optimal interconnect [12, 13]. Monosulfide TiS exhibits $P\bar{6}m2$ symmetry (space group 187) with ${\mathrm{TiS}}_{6}$ triangular prismatic coordination. The trigonal prisms are edge and face sharing within the a−b plane and along the c axes of the hexagonal structure (Figure 3). Nominally, the electronic structure is characterized by a singlet state with electrons from ${\mathrm{Ti}}^{2+}$ ($d^{2}$ electronic configuration) imposed by the $D_{3h}$ crystal field and is obtained by filling the lowest energy $a_{1}^{\prime }$ symmetry orbital with $d(z^{2})$ character (Figure S3, Supporting Information). The fully occupied S 3p orbitals are found below the Ti 3d states. Moderate mixing occurs with the $e^{\prime }$ orbitals with $d(x^{2}-y^{2})$ and d(xy) character owing to π‐bonding interactions [58] and flattening of the ${\mathrm{TiS}}_{6}$ trigonal pyramids [59]. Thus, the low energy electronic structure comprises a mixture of the $a_{1}^{\prime }$ and $e^{\prime }$ orbitals, making it a self‐doped topological semimetal.

We further calculate the resistivity scaling coefficient, $\rho_{0}\lambda$, where $\rho_{0}$ is the bulk resistivity and accessible from first‐principles [38]. In a trivial metal, the system with the lowest value of $\rho_{0}\lambda$ is expected to support the lowest nanowire resistivity. For details see Discussion VII (Supporting Information). This descriptor has been widely used for identifying promising trivial conductors for interconnect applications [1], and is $37\times 10^{-16}\;\Omega \;m^{2}$ and $41\times 10^{-16}\;\Omega \;m^{2}$ for the [100] and [001] transport directions of TiS. This value is computed accounting for spin‐orbit coupling and is comparable to the best‐of‐class anisotropic conductors such as delafossite ${\mathrm{PtCoO}}_{2}$ ($\rho_{0}\lambda =13.3\times 10^{-16}\;\Omega \;m^{2}$ [60]). To the best of our knowledge, no synthesis or transport measurements have been reported for TiS; moreover, the compound is thermodynamically stable, as it appears on the Ti–S convex hull reported by the Materials Project [30]. Furthermore, TiS is unlikely to oxidize upon contact with ${\mathrm{SiO}}_{2}$, which we use as a simplified proxy for the low‐k dielectric SICOH. The formation of a tie‐line between TiS and ${\mathrm{SiO}}_{2}$ suggests that the compound can exist in stable equilibrium with the dielectric and is unlikely to undergo chemical reactions; however, oxidation in the presence of $O_{2(g)}$ could favor oxysulfide formation.

${\mathrm{ZrB}}_{2}$ crystallizes in the hexagonal ${\mathrm{AlB}}_{2}$ structure type with P6/mmm symmetry (space group 191), featuring hexagonal layers of Zr atoms interleaved between borophene sheets. Although the nominal electronic configuration of ${\mathrm{Zr}}^{4+}$ ($d^{0}$) and $sp^{2}$‐hybridized B orbitals for its constituents suggests an insulating state, delocalized Zr–B dpπ bonding interactions result in a semimetallic character. This leads to slight doping of the Zr 4d states and several band crossings near $E_{F}$, as evident in the band dispersions (Figure S4, Supporting Information). Previous studies have classified ${\mathrm{ZrB}}_{2}$ as a nodal‐line semimetal [20, 61], and have demonstrated low resistivity in the thin‐film limit [62, 63], consistent with our nanowire transmission analysis. Here we calculate $\rho_{0}\lambda =12\times 10^{-16}\;\Omega \;m^{2}$ and $\rho_{0}\lambda =11\times 10^{-16}\;\Omega \;m^{2}$ for the [100] and [001] transport directions of ${\mathrm{ZrB}}_{2}$. These values are reduced with respect to TiS, a reflection of the greater Fermi surface area due to additional bulk bands near the Fermi energy as illustrated in the Supporting Information [64]. Similar to TiS, ${\mathrm{ZrB}}_{2}$ lies on the convex hull and is in phase equilibrium with ${\mathrm{SiO}}_{2}$, indicating its chemical stability and compatibility with dielectric environments.

The AN mononitrides where A=(Mo,Ta,W) exhibit $P\bar{6}m2$ symmetry with $AN_{6}$ triangular prismatic coordination. The trigonal prisms share edges and faces within the a−b plane and stack along the c axis of the hexagonal structure. Nominally, the electronic structure is characterized by $A^{3+}$ transition metals within a $D_{3h}$ crystal field. Strong hybridization occurs between the N 2p states and 4d (5d) states of Mo (Ta,W), leading to a complex low‐energy electronic band structure dominated by d states (Figure S2, Supporting Information).

Prior work has studied this family of materials and identified it as hosting a topological triple point near the Fermi energy [11] with ultra‐high conductivity shown for MoP [12], which falls in this family and has been proposed as an emerging interconnect material [13]. We calculate $\rho_{0}\lambda =2.8\times 10^{-16}\;\Omega \;m^{2}$ and $\rho_{0}\lambda =2.1\times 10^{-16}\;\Omega \;m^{2}$ for the [100] and [001] transport directions of MoN. These values reflect the large conductivity offered by the large Fermi surface area as a topological metal (Discussion II, Supporting Information).

Figure 3 compares the surface spectral density of TiS, ${\mathrm{ZrB}}_{2}$, and MoN with that of MoP and the *M*Pn compounds at the Fermi energy. The analyzed surfaces correspond to the (100) orientation for TiS, ${\mathrm{ZrB}}_{2}$, and MoP; the (001) orientation for MoN and the *M*Pn compounds. A common feature for all the TSMs is the presence of a network of Fermi arcs beginning and terminating at the projection of the bulk band crossing locations; in contrast to the Dirac cone surface states of topological insulators. This is notable as the open‐line character of Fermi‐arcs is expected to enhance protection against disorder and perturbations, e.g., surface roughness, that may arise during the synthesis of physical nanowires [34, 65, 66].

**FIGURE 4:**
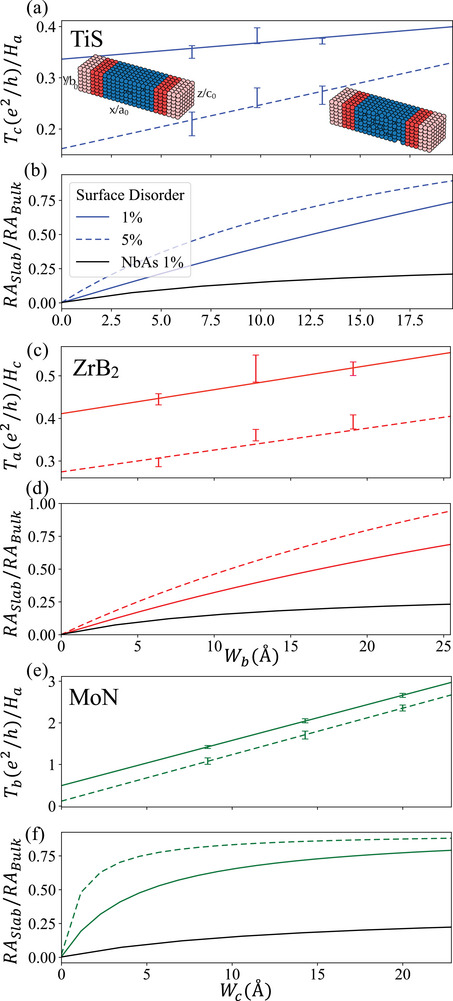
Surface disorder effects on transmission and resistance‐area product scaling. Nanowire surface transmission as a function of surface vacancy density in (a) TiS, (c) ${\mathrm{ZrB}}_{2}$, and (e) MoN at the Fermi energy. In both systems we utilize the geometry which yielded maximal surface transmission in the initial screening. Ratio of slab‐to‐bulk resistance‐area product for (b) TiS, (d) ${\mathrm{ZrB}}_{2}$, and (f) MoN as a function of the slab surface roughness compared to NbAs with 1% surface vacancy density utilizing the geometry which yielded maximal surface transmission. Inset of (a) displays example of nanowire with unit cells removed from the surface to simulate disorder. Example configurations show 1% and 10% surface vacancy density for clarity.

To test the robustness of the surface transmission against disorder, we simulate surface roughness in our nanowire transmission calculations for TiS, ${\mathrm{ZrB}}_{2}$, and MoN by randomly removing 1% and 5% of the surface unit cells prior to evaluating the transmission at the Fermi energy (Figure 4). Here we use nanowire geometries that produced maximal $T_{S}$ in our initial screening. We repeat this simulation across at least 100 random configurations per vacancy density, and results are averaged to assess the statistical reliability. We focus on surface vacancies as the effect of bulk vacancies on surface transmission in a topological semimetal was studied previously; it was found to cause minimal deviation from the pristine case [2, 24, 26]. We expect the protocol implemented in this work to capture weak disorder effects and surface roughness that produces height‐variations up to a single unit cell. However, we do not expect to capture the effects of grain boundaries, height variations beyond a unit cell, and other more complex surface morphologies that require dedicated studies. We further refer the reader to Ref. [2] for an alternative analysis of surface defect lines in TSM NbAs.

Rather than comparing absolute transmission values, we focus on the relative decrease in surface transmission. If the surface conduction channels were not topologically protected, surface disorder would induce localization, driving $T_{s}\to 0$; however, as shown in Figure 4a,c,e, this behavior is not observed, indicating that $T_{S}>0$ arises from topologically protected states. Upon increasing the surface vacancy density from 1% to 5%, we find a reduction in $T_{S}$ of 48%, 65%, and 75% for TiS, ${\mathrm{ZrB}}_{2}$, and MoN respectively. In contrast, similar calculations for NbAs show a reduction of $T_{S}$ by 51% (see Figure S5, Supporting Information), suggesting that the extended surface states in TiS may offer greater protection to surface roughness in the ultra thin limit and 0 K limit, where no surface and bulk phonon scattering is absent.

Next, we examine the resistance‐area scaling as a function of nanowire thickness [24], to better understand at which dimensions the surface states dominate transmission over the bulk. The slab resistance area, $RA_{Slab}=A/\left(T_{S}+W\;g_{\mathrm{bulk}}\right)$, where the denominator follows Equation 1. Upon substituting A=WH, we extract $RA_{\mathrm{bulk}}$ by taking the limit W→∞. The normalized RA product then can be approximated as ${\left(RA\right)}_{\mathrm{slab}}/{\left(RA\right)}_{\mathrm{bulk}}\approx {\left(1+\alpha /W\right)}^{-1}$, where α describes how much of the conductance is due to surface states versus bulk states. For a trivial conductor like copper, the ratio of these products is approximately unity (α→0) [24], and hence independent of slab thickness, because bulk states dominate the conduction. In our analysis, we additionally include surface roughness into the assessment using the data from Figure 4.

Figure 4b,d,f shows the ratio of the slab‐to‐bulk RA products for the nanowire with surface roughness, corresponding to 1% and 5% surface vacancy densities for TiS, ${\mathrm{ZrB}}_{2}$ and MoN respectively. As expected, surface roughness has the effect of increasing the RA product; however for TiS the rate at which it approaches the bulk limit appears reduced relative to ${\mathrm{ZrB}}_{2}$ and MoN. This is in part due to the increased Fermi surface area of ${\mathrm{ZrB}}_{2}$ and MoN (see Figure S2, Supporting Information). The greater number of bulk conducting channels reduces the ratio of surface to bulk conductance, α, despite the two materials exhibiting comparable surface transmission to TiS. Our numerical fits to the data give α=45, 10.2, and 8.03 in the pristine (zero roughness limit) for TiS, $\mathrm{Zr}B_{2}$ and MoN which are comparable to values simulated for pristine TaAs (α=28) and NbAs (α=60) [24]. This indicates that TiS could support enhanced stability to surface roughness/defects. It also suggests that TiS may suffer less from scattering between topological surface states and bulk states. As a near perfect semimetal, surface states dominate conductance in the thin film limit for NbAs, elevating α. This suggests methods to engineer the band structure and shift trivial bulk states away from the Fermi energy [67] may be beneficial for future optimization of compounds displaying high surface transmission.

### 3.2 Multi‐Variable Optimization

The outcome of the employed workflow is a dataset comprising zero‐temperature nanowire transmission values for all surfaces orthogonal to principal lattice vectors within the conventional unit cell, as a function of Fermi energy. While this data is vital for identifying promising interconnect candidates, it must be evaluated along with other practical considerations. These include crystallographic orientation and microstructure, self‐limiting oxidation behavior, finite‐temperature effects (e.g., bulk and surface phonon scattering and thermal expansion), and long‐term reliability concerns like electromigration.

Together, these considerations necessitate a multi‐variable optimization framework, for which we formulate a simplified approach to assess and rank interconnect materials under realistic operating conditions by optimizing over a subset requirements (Table 1). Specifically, we consider the transmission direction/surface orientation, surface transmission value ($T_{S}$, electron chemical potential, i.e., doping level relative to the Fermi level $\Delta E_{F}$), and surface energy (γ), which serves as a proxy for the ease of synthesizability on a given (hkl) surface. Although a Utopian composition seeks to simultaneously achieve ideal target values across all objectives, such a solution is typically unattainable in practice. Consequently, the Pareto front within this reduced parameter space enables a tractable yet physically meaningful optimization strategy, which can be combined with other parameters, for identifying viable interconnect candidates and delineating the inherent trade‐offs among competing objectives.

**TABLE 1:** Multiobjective design space. Representative subset of critical interconnect property objectives considered for optimization.

Objective	Variable	Target range
Transmission direction	[hkl]	Low index
Surface orientation	(hkl)	Low index
Surface transmission	$T_{S}$	maximum
Doping level away from $E_{F}$	$\Delta E_{F}$	∼0
Surface energy	$\gamma_{hkl}$	Small

Surface energies represent the only missing objective values in the surface transmission workflow previously described. Thus, we computed the surface energies for all (hkl) surfaces as $\gamma_{hkl}=\left(E_{\mathrm{slab}}^{hkl}-E_{\mathrm{bulk}}^{hkl}\times n_{\mathrm{slab}}\right)/\left(2\;A_{\mathrm{slab}}\right)$, where $E_{\mathrm{slab}}^{hkl}$ is the total energy of a slab exposing the (hkl) surface and $E_{\mathrm{bulk}}^{hkl}$ is the per atom total energy of the bulk unit cell oriented along the same direction, $n_{\mathrm{slab}}$ is the number of atoms in the slab, and A is the surface area of the slab [68]. For each material, we construct a slab consisting of five unit cells and insert 20 Å of vacuum to expose the surfaces. All surface energy calculations are carried out using the CHGNet machine learned interatomic potential (v0.3.0, [69]) for consistency. Prior work has shown that CHGNet achieves a root mean square error (RMSE) of 0.03 eV/Å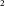 in predicting surface energies [70]. This error can influence ranking of interconnect candidates but remains sufficiently small to ensure strong candidates are not discarded in error. A comparison of the DFT and CHGNet computed surface energy for TiS and ${\mathrm{ZrB}}_{2}$ is available in Discussion IX (Supporting Information).

**FIGURE 5:**
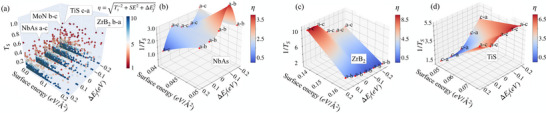
Pareto frontier candidates in the interconnect design space. (a) Scatter plot of all 3000 screened compound‐surface‐direction combinations, with each point labeled by its maximal surface transmission ($T_{S}$), surface energy, and Fermi level shift ($\Delta E_{F}$). Points are colored by $\eta ={\left(\Delta E_{F}^{2}+\gamma^{2}+R_{S}^{2}\right)}^{1/2}$, with $R_{S}=T_{S}^{-1}$, where lower values indicate optimal interconnect candidates. Selected data points are labeled by transmission direction and surface orientation. An interactive version of this figure is available online at Ref. [43]. Pareto subsurface for (b) NbAs, (c) TiS, and (d) ${\mathrm{ZrB}}_{2}$ showing interpolated trends in the $\Delta E_{F}-\gamma$ plane as a function of doping.

Figure 5a presents 3000 evaluated objective values within a 3D representation, where each coordinate x,y,z is given by the surface energy, doping level, and surface transmission for a compound ($\gamma_{hkl},\Delta E_{F},T_{S}$). Within this design space, we aim to identify an optimal interconnect candidate that satisfies the following criteria: (i) Requires no doping, i.e., the maximum $T_{S}$ occurs at the Fermi energy such that $\Delta E_{F}=0$, (ii) Exhibits minimal surface energy, with Cu serving as a reference exhibiting experimental surface energies of 0.08 eV/Å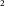 [71], which is close to our simulated value of ∼0.06 eV/Å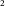), and (iii) maximal surface transmission, corresponding to minimal surface resistance $R_{S}=T_{S}^{-1}$. We combine these objectives into a figure‐of‐merit, η, which quantifies the Euclidean distance of the ideal point in the 3D design space: $\eta ={\left(\Delta E_{F}^{2}+\gamma^{2}+R_{S}^{2}\right)}^{1/2}$. Minimizing η thus recasts the problem of identifying an optimal interconnect candidate into a tractable multi‐objective optimization task.

Among the optimal compounds identified in Figure 1 by examining $T_{S}$ alone, we find that the known Weyl semimetal NbAs and our identified compounds TiS, ${\mathrm{ZrB}}_{2}$, and MoN appear on the Pareto front (Figure 5a). Other promising compounds including CoSi, NbP, and TaP fall among these compounds, represented as dark red points. They can be seen by accessing the interactive version of this figure at https://mtd.mccormick.northwestern.edu/interconnect‐database. In addition to examining the Pareto front, we further examine the subspace manifold for a given compound as shown for NbAs, TiS, and ${\mathrm{ZrB}}_{2}$ (Figure 5b–d). Here we directly plot $R_{S}$, γ, and $\Delta E_{F}$ and to evaluate the anisotropy with doping away from the Fermi level, we construct an interpolating function connecting data points in the $\Delta E_{F}-\gamma$ plane and color this surface according to the value of η. We note that the Pareto designs are not being interpolated from the constrained design space given that $\gamma_{hkl}$ is a nonlinear function of h,k,l.

For NbAs (Figure 5b) we find that the (001) surface supports a lower surface energy, making it more likely to be experimentally accessible. In addition, nanowire transmission due to surface states on the (001) surface is also maximal. Optimization of NbAs then reduces to tracking the Pareto Front produced by the interpolated surface as a function of doping. In contrast, the design manifold for ${\mathrm{ZrB}}_{2}$ (Figure 5c) shows that the highest $T_{S}$ is along [100] or [001] for the (010) and (100) surfaces, respectively, however, the (001) has lowest surface energy, which makes it most accessible. On both surfaces we find that doping below the Fermi energy can enhance surface transmission. Figure 5d shows that transmission due to surface states on the (100) surface is maximal for TiS. Advantageously, the (100) surface also supports a minimal surface energy. We further note that surface transmission is maximal at $E_{F}$ and remains high when doping below it but not above.The tradeoff among accessible surface orientations and optimal transmission direction shows the complexity in finding an interconnect compound that satisfies all design objectives and informing subsequent manufacturing, i.e., whether growth in trenches or blanket films followed by pattering would be pursued.

### 3.3 Chemical Features for Compound Discovery

**FIGURE 6:**
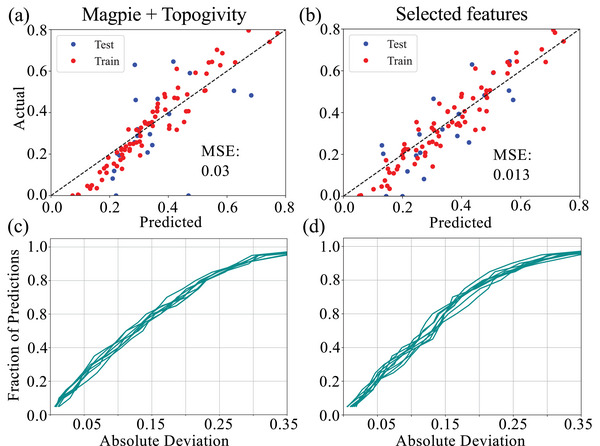
Machine learning surface transmission. (a) Results of random forest model trained to predict surface transmission using all Magpie [48] features as well as the topogivity index of a given compound [50]. (b) Refined random forest model trained to predict maximal surface transmission using only most important features, topogivity, mode Mendeleev number of the constituent elements and space group number. (c,d) Regression error characteristic (REC) curve for model trained (c) using all Magpie features and topogivity and (d) only most important features. Each colored line represents the median REC curve from one of 10 random seeds. For each seed, a fivefold cross‐validation is carried out.

This work has significantly expanded the number of materials for which surface transmission has been screened and provided a workflow that can be automated without sacrificing accuracy. A benefit of such progress is the ability to apply data mining strategies and create a machine‐learning network to rapidly explore larger areas of material space, including virtual materials databases such as Gnome [72] to identify optimal interconnect candidates. Using a tree‐based model with a random 80%/20% train/test split, we obtained reasonable performance of our model with a mean‐squared‐error (MSE) of 0.03 using all possible Magpie features and the topogivity index of the compound (Figure 6a). By examining feature importance, we find the most influential descriptors are the Mendeleev numbers of the constituent atoms, the topogivity index, space group, and the number of d electrons in the valence shell. These features are intuitive as that provide insight into the topological nature of the compound. For example, the space group symmetry is relevant to the presence of non‐trivial electronic band topology as non‐magnetic Weyl semimetals require lifting of inversion symmetry. Retraining the model using only these key features further improves the performance as shown in Figure 6b. Despite the relatively small dataset, the model demonstrates reasonable accuracy, suggesting that our approach can be extended to efficiently down select broad compositions spaces for high‐throughput screening in future interconnect searches.

## 4 Summary and Outlook

In this work,, we have proposed and implemented a workflow in an effort to identify optimal topological conductors for use as interconnects in next‐generation integrated circuits. Our proposed workflow relies on utilization of Wannier tight‐binding models which replicate the electronic structure of the bulk material precisely, however by working in a Wannier basis it is possible to exploit sparse matrix methods to access large system sizes in computation of transmission via the NEGF formalism. We have identified TiS, ${\mathrm{ZrB}}_{2}$, and AN (A=Mo,Ta,W) to be added to the list of potentially optimal interconnect candidates which already includes CoSi, and *M*Pn, M=(Nb,Ta) and Pn=(P,As). We have further investigated the degree to which the computed surface transmission is robust to surface roughness. Our findings suggest that a maximal value of surface transmission is a high‐priority metric and can be correlated with a large number of chiral Fermi arcs as found in prior works. However, our screening also brings to light the impact of additional bulk bands in the vicinity of the Fermi energy. No existing candidate is an ideal TSM with a point‐like Fermi surface so the impact of additional bulk bands which intersect the Fermi energy is important to consider. While these bands do not impact the surface transmission, they decrease two other key metrics, α and $\rho_{0}\lambda$ through an increase in the bulk conductance.

While a decreased value of $\rho_{0}\lambda$ is beneficial for a trivial interconnect candidate due to lower bulk resistance, a decreased value of α is viewed negatively for a topological interconnect candidate as it suggests surface states may not remain the dominant conducting channel for finite thickness films. This situation is particularly relevant for topological metals like MoN. While topological metals are identified as interconnect candidates by the surface transmission values computed in this work, the computed value of $\rho_{0}\lambda$ indicates it is the most important metric as the bulk will dominate nanowire transmission. This situation underscores the need to study the multivariable Pareto frontier and leverage the protection against localization offered to bulk states as well as surface states by nontrivial topology. A detailed protocol for direct prediction of nanowire resistivity at realistic dimensions, which requires incorporation of all metrics presented here, is the subject of a future work. In addition, crystallographic orientation is critical for maximizing $T_{S}$, given its strong anisotropy. Our simulations suggest robustness to surface roughness and disorder, supporting feasibility under realistic fabrication conditions; however, experimental validation is essential to assess long‐term reliability.

Future work to extend this protocol to further ternary compounds will benefit from refinement of the nanowire transmission computations, primarily through efficient parallelization of the non‐equilibrium Greens function formalism. Additionally the potential to utilize foundational machine learning networks for generation of the Hamiltonian, such as those presented in Refs. [73, 74], may provide an avenue for efficiently screening a large number of materials. Enhancing computational efficiency is critical for enabling finite‐temperature transport calculations. At present, the computational cost of these simulations exceeds available resources due to the requirement of first‐principles phonon mode calculations for large slabs or nanowires, followed by electron–phonon transport evaluations using the EPW package [75, 76]. Magnetic semimetals also present a promising class of material which has been reserved for future study. While a number of magnetic TSMs have been experimentally synthesized [77, 78, 79, 80, 81, 82, 83], the impact of thermal fluctuations and defects on the magnetic ordering and Fermi arc states must be carefully studied.

Beyond intrinsic properties evaluated in this work, practical integration of the proposed compounds into interconnect technologies requires consideration of process compatibility (with back‐end‐of‐line (BEOL) temperatures), orientation control for maximizing transmission, electromigration reliability, diffusion barrier properties, liner/wetting layer and adhesion layer. State‐of‐the‐art interconnects use Cu embedded in low‐K dielectrics like SiCOH, with multilayer stacks including TaN barriers and Co wetting layers. Emerging materials such as MoN, TiS and ${\mathrm{ZrB}}_{2}$ may reduce or eliminate the need for these layers due to superior diffusion resistance (see Discussion VIII, Supporting Information). For example, binary MoP exhibits enhanced electromigration resistance [13], potentially simplifying BEOL integration. Future first‐principles studies on the compounds identified in this work should prioritize evaluating atomic diffusion barriers, as these are critical for assessing electromigration reliability under operational conditions. Achieving the correct phase and desired crystallographic orientation is essential for optimal performance and must be considered alongside the availability of reactive ion etching, wet etch, and chemical mechanical polishing chemistries. Area‐selective deposition of topological metals offers potential for contact formation beyond the damascene process, where oxidation effects should be considered.

## 5 Methods

We perform density functional theory (DFT) computations with spin‐orbit coupling (SOC) using the Quantum Espresso software package [46, 47], and fully relativistic norm‐conserving pseudopotentials from the PseudoDojo library [84] along with a maximal plane‐wave energy cutoff of 60 Ry. The Brillouin zone was sampled using a 9 × 9 × 9 Monkhorst–Pack k‐point grid [85]. This grid is sufficient for convergence when considering the smallest unit cell considered in this work. The choice of a uniform grid is made to prioritize the creation of high‐quality Wannier tight‐binding models rather than maximize computationally efficiency.

For the initial screening, the Wannier tight‐binding models are constructed using the selected columns of the density matrix (SCDM) protocol put forth in Ref. [45], allowing this process to be fully automated. Once constructed, the Wannier tight‐binding models are inspected for accuracy, determined by comparison of the band structure for the tight‐binding model and ab initio data within 2 eV of the Fermi energy and analysis of the Wannier center spread. We require the maximum Wannier center spread to be below $1.5\times |v_{max}|$ where $v_{max}$ is the largest magnitude lattice vector. The Wannier bulk band structure must also yield a mean average error, $\Delta E_{MAE}<0.05eV$ when compared to the DFT band structure with 2eV of the Fermi energy. If these conditions are met the second stage of the workflow is initiated, estimation of surface transmission.

## Conflicts of Interest

The authors declare no conflict of interest.

## Supporting information




**Supporting File**: advs73619‐sup‐0001‐SuppMat.pdf.

## Data Availability

The data that support the findings of this study are openly available in Dryad at https://doi.org/10.5061/dryad.12jm63zb7, reference number 64.
