# Supplementary material for: Accelerated Discovery of Topological Conductors for Nanoscale Interconnects
Source: Adv Sci (Weinh). 2026 Jan 14;13(10):e20535. doi: 10.1002/advs.202520535 (PMC12915232; doi:10.1002/advs.202520535)

SUPPLEMENTARY INFORMATION

Accelerated Discovery of Topological Conductors for Nanoscale Interconnects

Alexander C. Tyner,[1,2] William Rogers,[3] Po-Hsin Shih,[4] Yi-Hsin Tu,[5]
Gengchiau Liang,[5] Hsin Lin,[6] Ching-Tzu Chen,[4] and James M. Rondinelli[7]

[1]*NORDITA, KTH Royal Institute of Technology and Stockholm University, Stockholm, Sweden*
[2]*Department of Physics, University of Connecticut, Storrs, CT, USA*
[3]*Graduate Program in Applied Physics, Northwestern University, Evanston, IL, 60208, USA*
[4]*IBM Thomas J. Watson Research Center, Yorktown Heights, NY, USA*
[5]*Industry Academia Innovation School, National Yang Ming Chiao Tung University, Hsinchu, Taiwan*
[6]*Institute of Physics, Academia Sinica, Taipei, Taiwan*
[7]*Department of Materials Science and Engineering, Northwestern University, Evanston, IL, USA*

(Dated: December 9, 2025)

CONTENTS

I. Dataset Details 2

II. Surface and bulk electronic structure of TiS, ZrB_2, NbAs, MoN and MoP 3

III. Dependence of surface transmission on Wannier basis choice 4

IV. Assessing the quality of linear transmission scaling 6

V. Robustness of topological surface states to impurities and disorder 7

VI. Effect of surface vacancies on NbAs 8

VII. Computation of $\rho\lambda$ product 9

VIII. Computation of electromigration barrier 9

IX. Assessing surface energies from CHGNet 10

X. Summary of results 11

I. DATASET DETAILS

We restrict this work to mainly binary and elemental topological conductors with selected ternaries for purposes of computational efficiency, primarily due to the high memory demands of the nanowire transmission computation via non-equilibrium Greens function which remains a computational bottleneck. We reiterate that compounds that have either been experimentally synthesized or exhibit evidence of non-trivial bulk topology in prior computational studies are prioritized.

Supplementary Fig. 1 provides information regarding the breakdown of the dataset in terms of elemental composition and space group. A full list of the candidate materials is available in section X and online at https://doi.org/10.5061/dryad.12jm63zb7.

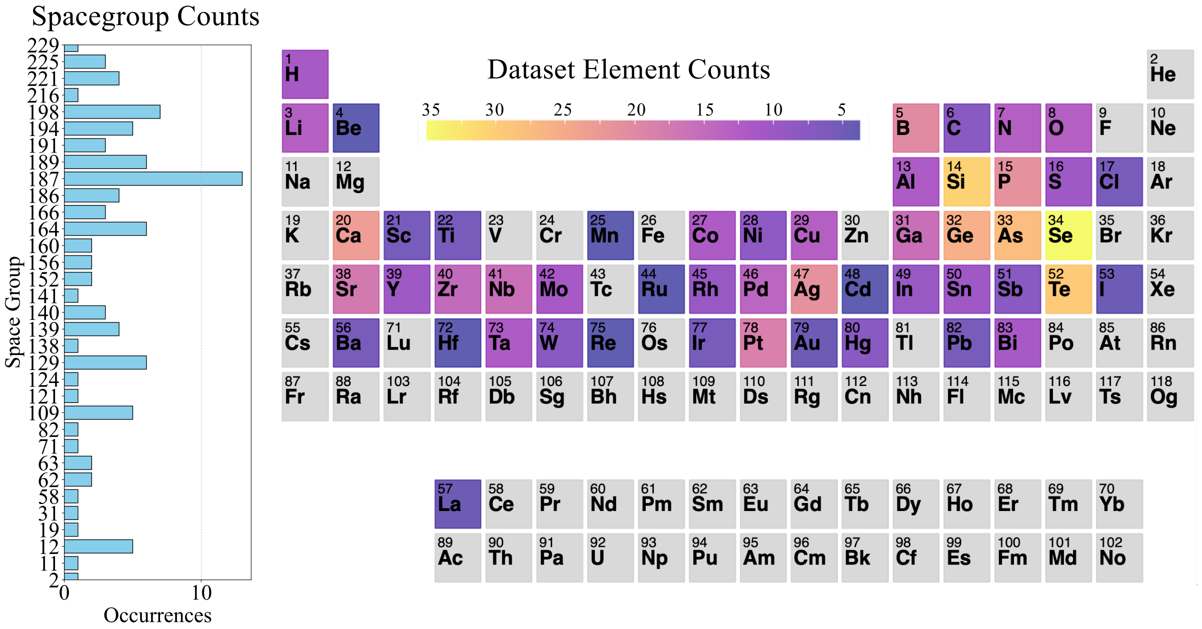

Supplementary Figure 1 | Dataset chemistry and space group diversity. Occurrence of each space-group and element among the dataset of candidate interconnects explored in this work.

II. SURFACE AND BULK ELECTRONIC STRUCTURE OF TIS, ZrB_2, NbAs, MoN AND MoP

TiS, ZrB_2 and MoN are identified as promising interconnect candidates and compared to MoP and NbAs topological materials which have been proposed as interconnect candidates in prior works. Here we provide the bulk band structure (Supplementary Fig. 2a-e), the surface spectral density (Supplementary Fig. 2f-j), and the bulk Fermi surface (Supplementary Fig. 2k-o) allowing for a direct comparison among the three compounds.

We emphasize that although all compounds support Fermi arcs, as detailed in Fig. (3) of the main body, both TiS, ZrB_2, MoN and MoP exhibit a greater number of bulk bands that intersect with the Fermi energy when compared to NbAs. This leads to a larger Fermi surface as seen in Supplementary Fig. 2k-o, and contributes to the decreased value of α relative to NbAs.

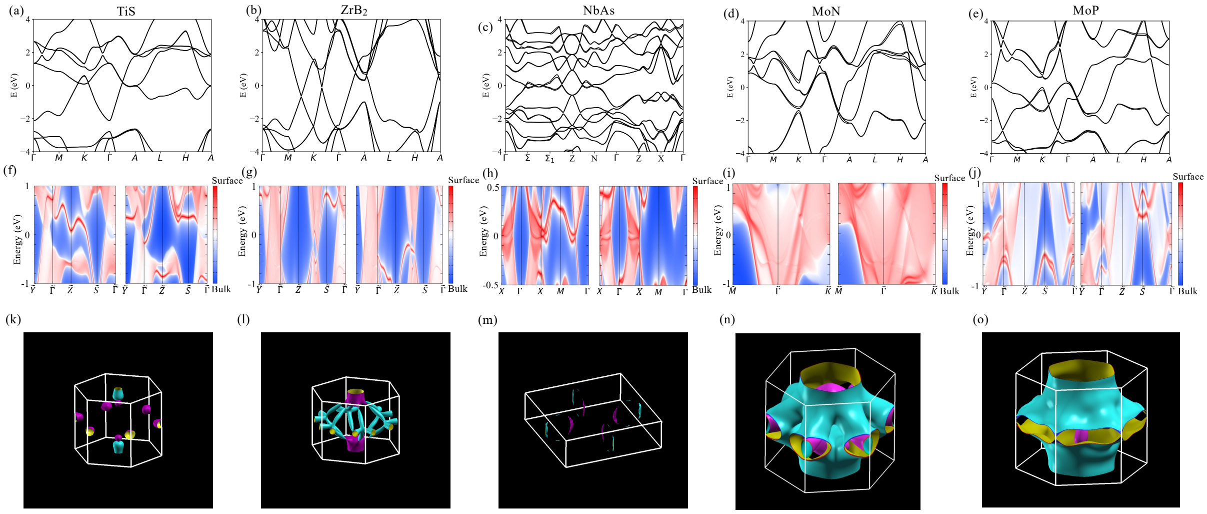

Supplementary Figure 2 | Electronic properties of select interconnect compounds. (a)-(e) Bulk band structure of TiS, ZrB_2, NbAs, MoN and MoP respectively along high-symmetry paths in the Brillouin zone. (f)-(g) (100) surface spectral density along a high-symmetry path on the surface for TiS and ZrB_2 respectively. The spectral density for the top and bottom surfaces of the slab are shown on the left and right sides respectively. (h)-(i) (001) surface spectral density along a high-symmetry path on the surface for NbAs and MoN respectively. The spectral density for the top and bottom surfaces of the slab are shown on the left and right sides respectively. (j) (100) surface spectral density along a high-symmetry path on the surface for MoP. (k)-(o) Bulk Fermi surface for TiS, ZrB_2, NbAs, MoN and MoP respectively. Different colors are assigned to contributions from distinct bands.

III. DEPENDENCE OF SURFACE TRANSMISSION ON WANNIER BASIS CHOICE

The nanowire transmission computations employed in this work require the electronic structure to be transformed into a Wannier basis from the plane-wave basis utilized by Quantum Espresso via the Wannier90 software package [?]. The Wannierization process requires selecting a set of orbitals for each atom which constitute to the basis for the Wannier tight-binding model. One option for creation of high-quality tight-binding models is to consider all possible orbitals. While effective for production of high-quality Wannier tight-binding models, this approach increases the computational expense of the subsequent transmission computations by incorporating occupied bands far below the Fermi energy; increasing the degrees of freedom for the sparse matrix representing the electronic structure of a single unit cell. A computationally advantageous approach is to consider only a subset of orbitals which dominate the density of states near the Fermi energy. This leads to Wannier tight-binding models with considerably less degrees of freedom, limiting the computational expense of subsequent transmission computations.

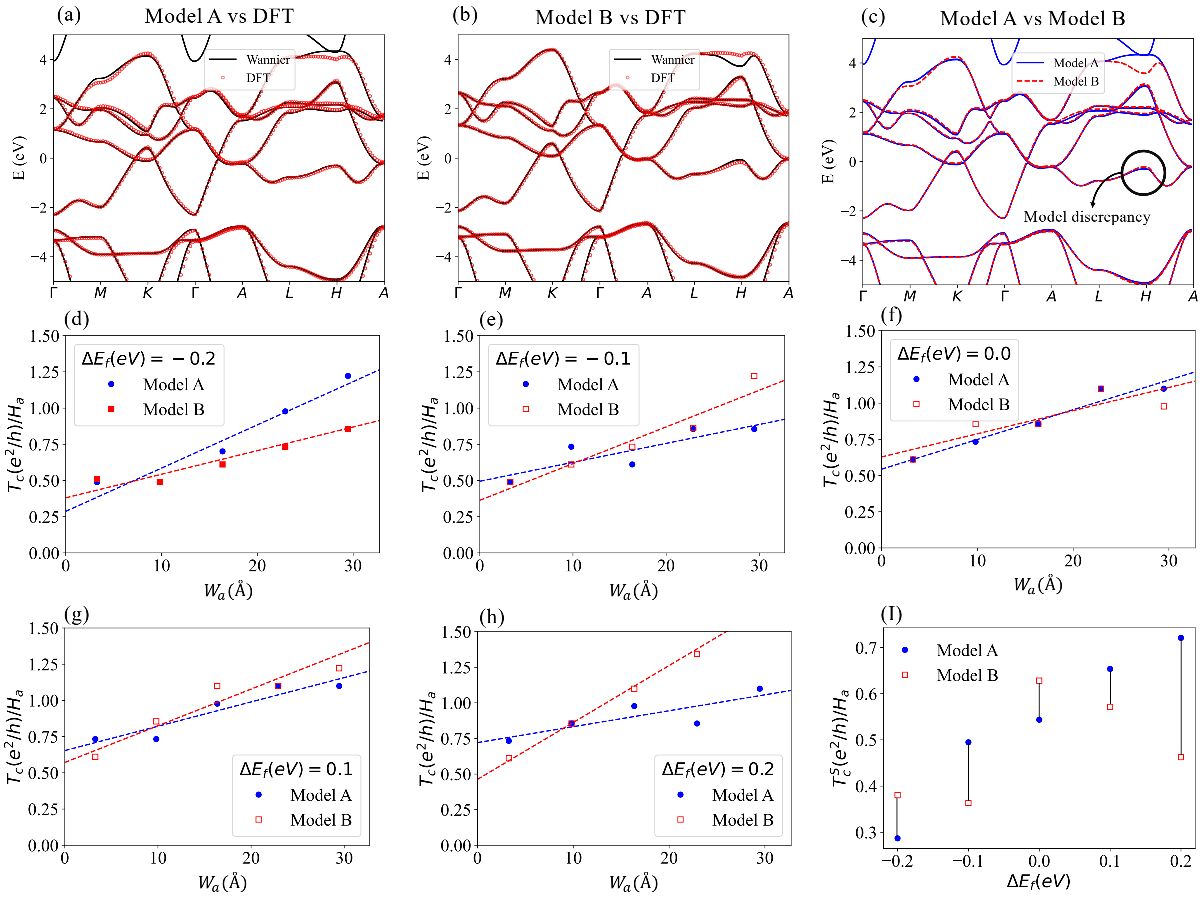

Supplementary Figure 3 | Wannier basis sensitivity analysis: TiS. (a)-(b) Bulk band structure of Wannier tight-binding model using (a) only d-orbitals for Ti and p-orbitals for S and (b) s, p, d-orbitals for Ti and s, p-orbitals for S, compared to density functional theory band structure along a high-symmetry path in the Brillouin zone. (c) Comparison of the bulk band structure between the two Wannier tight-binding models. (d)-(h) Nanowire transmission as a function of thickness for the two tight-binding models varying the doping between $\Delta E_f = -0.2$ and $\Delta E_f = 0.2$. (I) Extracted value of surface transmission for both Wannier tight-binding models as a function of doping.

In this work, we first prioritize accuracy of the tight-binding model, as measured by faithful reproduction of the DFT band structure within 1 eV of the Fermi energy. The secondary goal is to use as few orbitals as possible to accomplish this, limiting the computational expense of the nanowire transmission calculations. To understand how this approach can influence the results of computing nanowire transmission we consider first the case of TiS, constructing two Wannier tight-binding models. The first, denoted model A, incorporates s, p, and d orbitals for Ti and s and p orbitals for S, the second, denoted model B, incorporates only d orbitals for Ti and p orbitals for S. The resulting band structure of the two models compared to the DFT band structure and each other is shown in Supplementary Fig. 3a-c. Both structures reproduce the DFT band structure with high accuracy, the main discrepancy is marked by a black circle in Supplementary Fig. 3c.

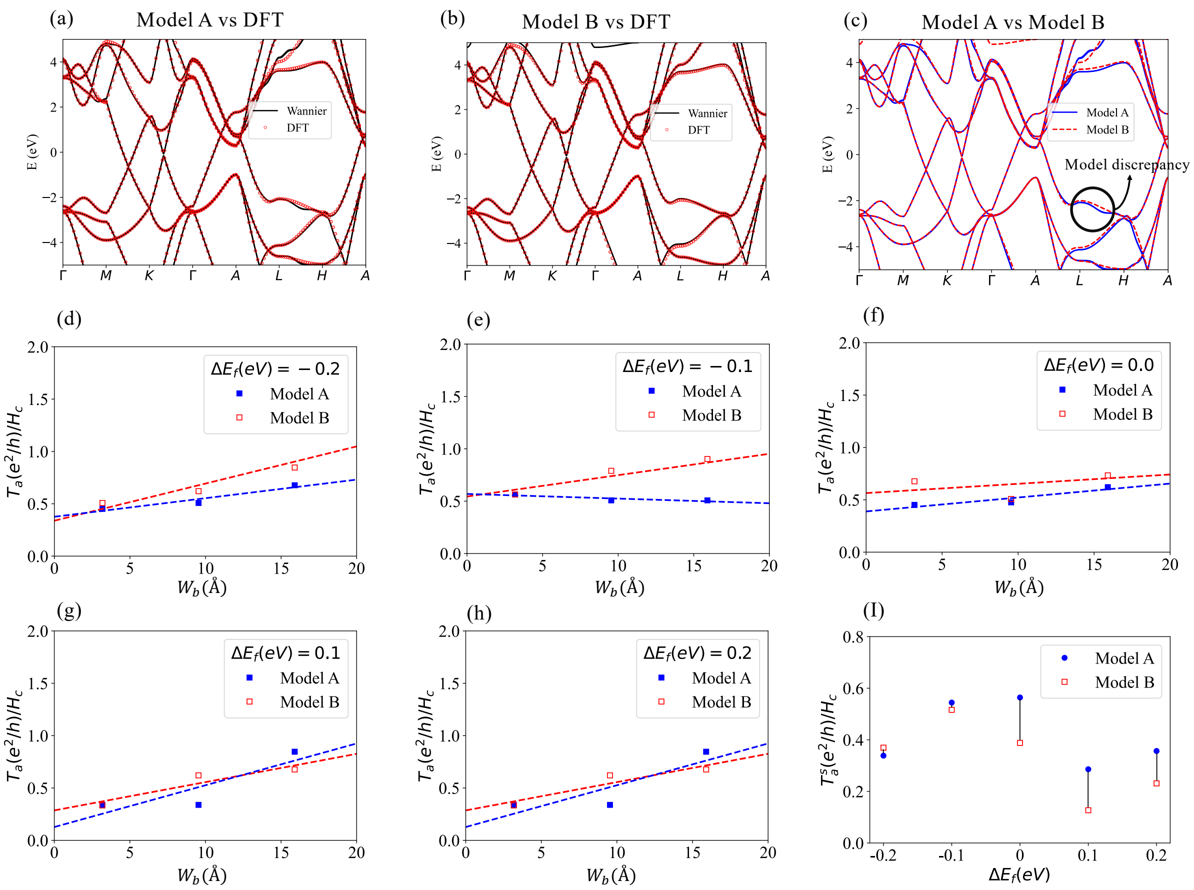

Supplementary Figure 4 | Wannier basis sensitivity analysis: ZrB$_2$. (a)-(b) Bulk band structure of Wannier tight-binding model using (a) only d-orbitals for Zr and p-orbitals for B and (b) s, p, d-orbitals for Zr and s, p-orbitals for B, compared to density functional theory band structure along a high-symmetry path in the Brillouin zone. (c) Comparison of the bulk band structure between the two Wannier tight-binding models. (d)-(h) Nanowire transmission as a function of thickness for the two tight-binding models varying the doping between $\Delta E_f = -0.2$ and $\Delta E_f = 0.2$. (I) Extracted value of surface transmission for both Wannier tight-binding models as a function of doping.

It is now important to understand how the basis choice impacts the resulting values of nanowire transmission. To do so, we compute transmission along the [001] direction due to states on the (010) surface. We select this transmissions direction for comparison as it was found to support maximal surface transmission in the main body. The results for Model A and Model B as a function of doping are shown in Supplementary Fig. 3d-h with the extracted values of the surface transmission as a function of doping compared in Supplementary Fig. 3I. We note that the extracted values of the surface transmission are within 15% for doping of 0.1 eV. However, when considering a doping of 0.2 eV above the Fermi energy, the discrepancy between the two models increases. This is not surprising as utilizing a smaller subset of orbitals requires the Wannier90 software package to fit conduction bands which may support a partial orbital density of states due to orbitals not included in the provided basis. Nevertheless, it is important to observe that the maximum value of transmission measured within 0.1 eV of the Fermi energy is nearly identical between Model A and Model B, occurring at a doping of 0.1 eV and at the Fermi energy, respectively.

This analysis is repeated for ZrB$_2$ where Model A considers contribution of Zr d-orbitals and B p-orbitals while Model B considers s, p, d-orbitals of Zr and s, p-orbitals of B. The resulting band structures of the tight-binding model are compared to the DFT band structure and each other in Supplementary Fig. 4(a)-(c). Slight discrepancies in the band structure of the two models is visible both above and below the Fermi energy between the L and H point. The resulting nanowire transmission is compared between the models in Supplementary Fig. 4(d)-(i). Once again the discrepancy remains smallest below the Fermi energy however the extracted surface transmission remains comparable throughout the range of doping values sampled.

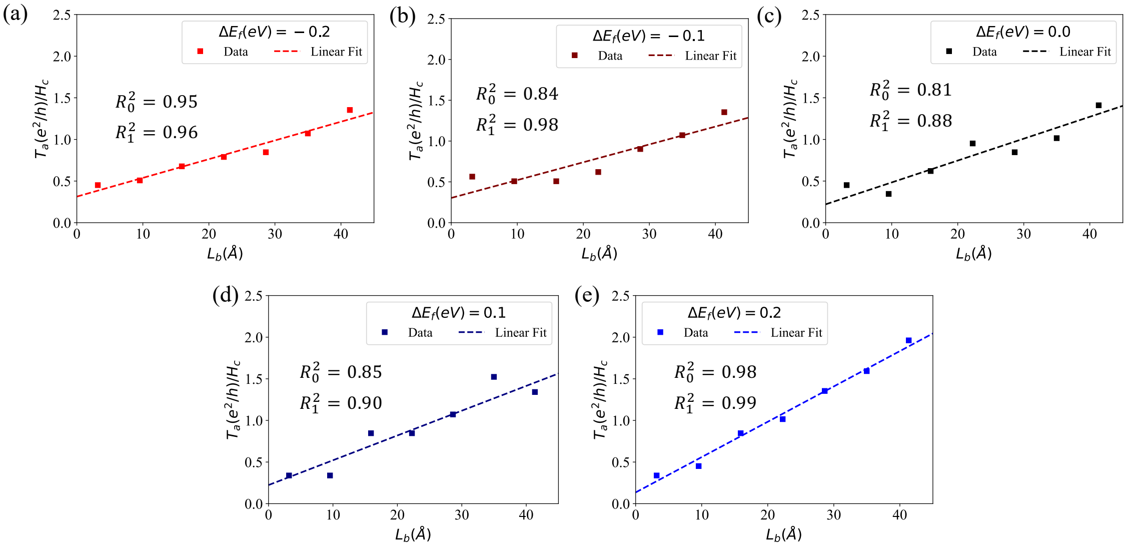

Supplementary Figure 5 | Assessing linear thickness scaling in TiS: The quality of a linear fit to transmission in TiS nanowires as a function of thickness at varying values of the Fermi energy is shown. The displayed linear fit considers all data points. Two values of R^2 are provided: R_0^2 assesses the quality of the linear fit considering all data points; R_1^2 assesses the quality of a linear fit after discarding the two data points at minimal thickness.

IV. ASSESSING THE QUALITY OF LINEAR TRANSMISSION SCALING

In this work surface transmission is extracted by performing a linear fit to the nanowire transmission as a function of wire thickness. In the ideal case, as the thickness of the wire is reduced the number of bulk conduction channels undergoes a corresponding linear reduction. However, reducing the thickness does not affect the nanowire surface of interest. As a result, a non-zero y-intercept upon performing a linear fitting of the nanowire transmission as a function of thickness should yield the surface transmission.

In this protocol it is important to consider the possibility that if the nanowire is not of a minimal thickness that quantum confinement effects can cause a deviation of this linear scaling, introducing error into the extracted value of surface transmission. In this section we have performed a more computationally expensive study of two promising interconnect candidates, TiS and ZrB_2, extending the maximum system size thickness to 13 unit cells and lowering the minimum thickness to a single unit cell. This is in contrast to the protocol utilized in this work where thickness is varied between 6 and 8 unit cells.

The results are shown for TiS and ZrB_2 in Fig. (5) and Fig. (6) respectively. In each case we list two R^2 values to judge the quality of the linear fit. The first, R_0^2, corresponds to the R^2 value upon fitting all data points. The second, R_1^2 corresponds to the R^2 value upon removing the first two data points, corresponding to one and three units cells of thickness. We note that fitting all the data points already returns a generally high R^2 value. However, there are examples where we see deviation from the linear thickness at the ultra-thin limit. A clear example of this is at $\Delta E_f = -0.1eV$ for ZrB_2, Fig. (6)(b), where the transmission is nearly constant between one and three unit cells. This may be a sign of the quantum confinement effects suggested by the reviewer. Importantly, such effects would be avoided in our work given the choice to use a minimum thickness of 6 unit cells.

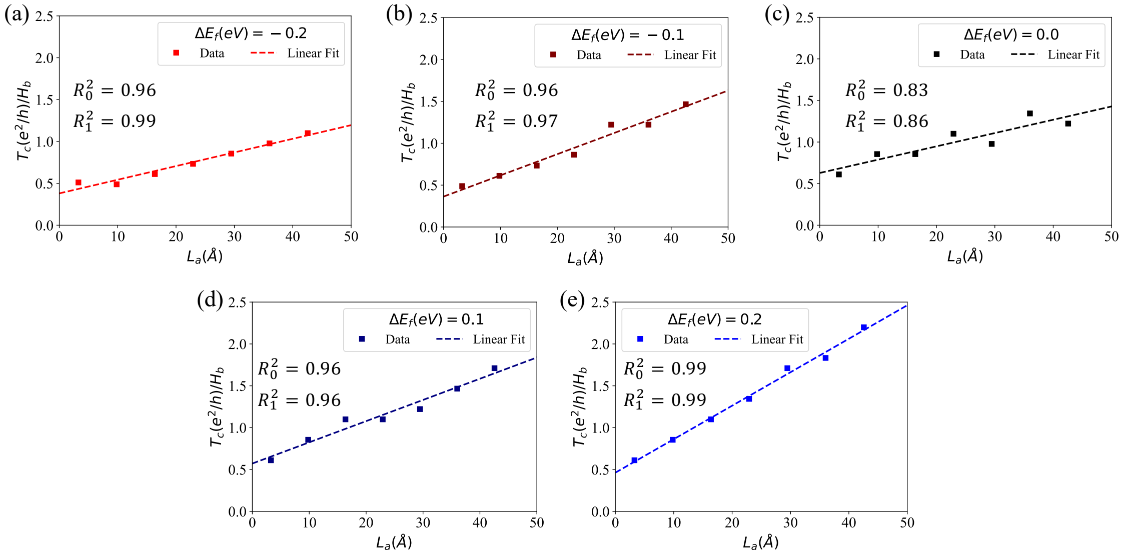

Supplementary Figure 6 | Assessing linear thickness scaling in ZrB_2: The quality of a linear fit to transmission in ZrB_2 nanowires as a function of thickness at varying values of the Fermi energy is shown. The displayed linear fit considers all data points. Two values of R^2 are provided: R_0^2 assesses the quality of the linear fir considering all data points; R_1^2 asses the quality of a linear fit after discarding the two data points at minimal thickness.

V. ROBUSTNESS OF TOPOLOGICAL SURFACE STATES TO IMPURITIES AND DISORDER

In the main body the utility of topological surface states as conducting channels for next-generation interconnects is explored. Such topological surface states are often referred to as "protected" and considered immune from back-scattering in the context of idealized low-energy models[**? ?**]. This is not true in general. In particular, for symmetry protected topological materials, the surface states are not protected from disorder which breaks the underlying symmetry[**? ? ? ?**]. Furthermore, realistic materials can support multiple states which cross the Fermi energy at the same transverse momenta. Such states can hybridize, opening scattering channels[**? ?**].

However, even in the case of symmetry protected topological systems, topological surface states have been shown to be less-susceptible to localization[**? ? ? ?**]. Their existence as conducting surface channels in the clean system is also guaranteed by the bulk topological invariant. This combination continues to make them strong candidates for next-generation interconnects despite the realistic considerations.

VI. EFFECT OF SURFACE VACANCIES ON NbAs

In Fig. 4 of the main body the effect of disorder, in the form or random surface vacancies, on the surface transmission is explored. In particular we explore the relative change in surface transmission as a function of surface vacancy density for TiS and ZrB_2. The relative decrease in the surface transmission as a function of the vacancy density is compared to NbAs, a promising candidate interconnect TSM which has been previously studied in Ref. [**?**].

In this section, we detail computation of nanowire transmission as a function of thickness in the presence of surface vacancies for NbAs at $\Delta E_f = -0.1eV$ where surface transmission is maximal. The results are show in Fig. Supplementary Fig. 7a, detailing that as the surface vacancy density is increased from one to five percent, the extracted surface vacancy density is reduced by approximately 51%. We additionally compute the ratio of the slab and bulk resistance-area product as a function of the surface vacancy density and display the results in Supplementary Fig. 7b. The result for one percent disorder has been displayed in the main body along side this ratio for the optimal nanowire geometry of TiS, ZrB_2 and MoN for comparison. This ratio increases at a reduced rate as a function of system size relative to TiS, ZrB_2 and MoN, due in part to the increased size of the NbAs bulk Fermi surface, allowing the Fermi arc states to serve as the dominate conducting channels at the Fermi energy.

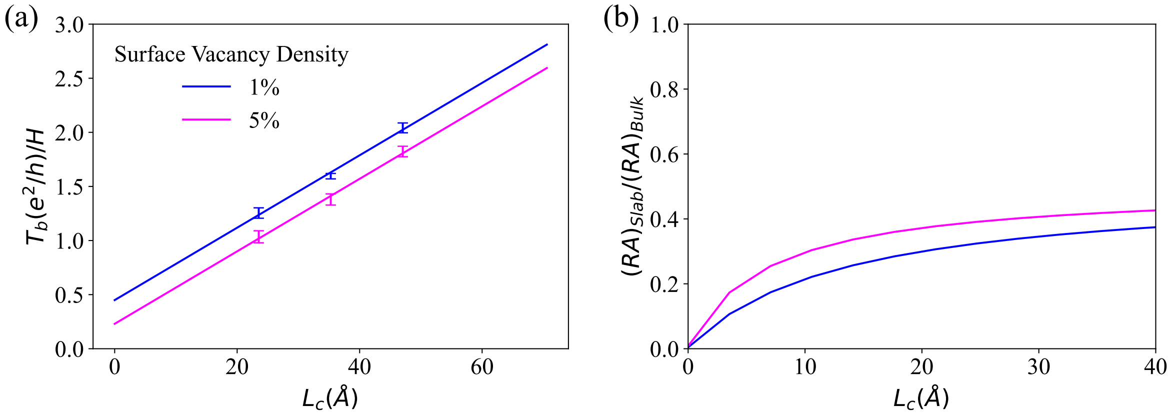

Supplementary Figure 7 | Disorder effects on transport proeprties of NbAs. (a) Nanowire surface transmission is extracted for 1% and 5% surface vacancy density in NbAs at $\Delta E_F = -0.1$ where surface transmission is maximal. (b) Utilizing the extracted value of surface and bulk transmission, corresponding to the intercept and slope of the linear fit to nanowire transmission as a function of system size respectively, the ratio of slab to bulk resistance area is plotted as a function of the slab surface roughness.

VII. COMPUTATION OF $\rho\lambda$ PRODUCT

The product of the bulk resistivity and mean-free path, $\rho\lambda$, is a value commonly used to identify optimal trivial conductors [?]. This quantity is computed for TiS ZrB_2 and MoN as,

$$\frac{1}{\rho\lambda} = \frac{e^2}{4\pi^3\hbar}\sum_n \iint_{S_F^n} \frac{v_{t,n}^2(\mathbf{k})}{\mathbf{v}_n^2(\mathbf{k})} dS, \tag{1}$$

where $\mathbf{v}_n(\mathbf{k}) = \frac{1}{\hbar}\nabla_k E_n(\mathbf{k})$ is the electron velocity of band n and S_F^n is the Fermi surface of band n. For cubic systems, this relation simplifies to, $\rho\lambda = 12\pi^3\hbar/(e^2 A_f)$ where A_f is the total Fermi surface area, indicating that an increased Fermi surface area lowers $\rho\lambda$.

We compute this quantity using a Γ centered $400 \times 400 \times 400$ grid of **k**-points. Visualizations of the Fermi surface for TiS, ZrB_2 and MoN are provided in Supplementary Fig. 2g-h. Immediately, we find an enhanced Fermi surface area of MoN and ZrB_2 relative to that of TiS, indicating a decreased value of the $\rho\lambda$ product.

We further note that spin-orbit coupling (SOC) is included in our computation to maintain a consistent level of theory across all computations. SOC is not included in prior works computing $\rho\lambda$, such as Refs. [? ? ?], and has the effect of generally increasing the Fermi surface area and lowering the value of $\rho\lambda$.

VIII. COMPUTATION OF ELECTROMIGRATION BARRIER

The electromigration barrier is computed using the Nudged Elastic Band (NEB) method. We consider a $4 \times 4 \times 4$ supercell with a nearest neighbor vacancy of the same species. As TiS, ZrB_2, MoN and MoP all support hexagonal unit cells, we consider vacancies along the c axis and a axis separately. The NEB path contains 11 points as the atom diffuses from the starting to ending vacancy site. We use Γ-point sampling due to the large size of the supercells and employ a plane-wave cutoff of 60 Ry.

The results are shown in Supplementary Fig. 8(a)-(d) along with the NEB computation for a $4 \times 4 \times 4$ supercell of Cu. In all cases, the electromigration barrier is higher than that for Cu vacancy diffusion.

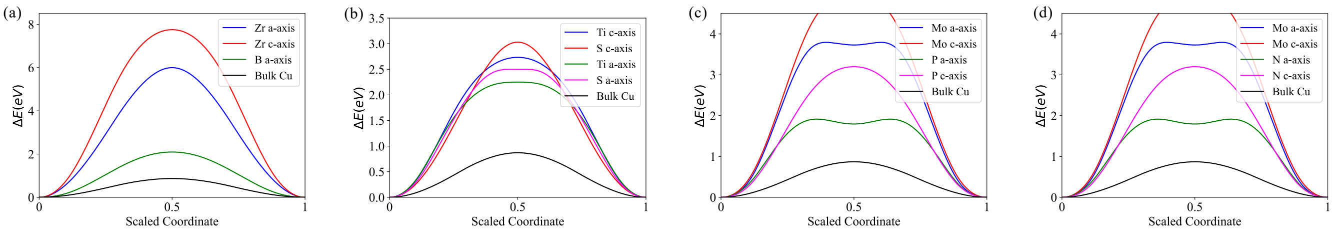

Supplementary Figure 8 | Electromigration barriers for select compounds. Computed electromigration barriers for diffusion between nearest neighbor vacancies of the same atomic species along the a and c axis for hexagonal systems ZrB_2, TiS, MoP, and MoN, respectively. In each system, we further compare to the electromigration barrier computed an in identical manner for bulk copper.

IX. ASSESSING SURFACE ENERGIES FROM CHGNET

In this work the foundational machine learned potential (MLP) CHGNet (v0.3.0) is utilized to compute the surface energy for all interconnect candidates. CHGNet is utilized as it provides computational efficiency with minimal loss in accuracy. The quality of CHGNet, as well as other MLPs has been investigated in Ref. [**?**], where it was shown that CHGNet achieves a root mean square error (RMSE) of $0.03 eV/\mathring{A}^2$. As stated in the main body, such error is sufficient to cause minor alterations in the Pareto frontier and impact rankings of interconnect candidates. However, this deviation is not of sufficient magnitude to classify in error a strong interconnect candidate suitable for further investigation as a weak candidate which should be discarded.

To verify further the use of CHGNet, we compute the (100) and (001) surface energy of TiS and ZrB_2 using QUantum Espresso. We have followed the same procedure utilized for determining the surface energy via CHGNet, namely relaxing a 6-layer oriented slab with $20\mathring{A}$ of vacuum. The atomic positions are relaxed until the forces are all below 0.01 eV/$\mathring{A}$ and in accordance with Ref. [**?**] using the Perdew-Berke-Ernzerhof (PBE) generalized gradient approximation (GGA) functional[**?**], and all calculations were spin-polarized with a plane wave cutoff energy of 80Ry. For the bulk and slab computations a **k**-point grid of $\{50/a, 50/b, 50/c\}$ and $\{50/a, 50/b, 1\}$ were implemented respectively. In this process we find the results shown in Tab. (I). These results demonstrate that CHGNet performs at an accuracy comparable with direct DFT computations.

In this table we note that the DFT and CHGNet computations exhibit a RMSE of 0.038 eV/($\mathring{A}^2$), this is in close agreement with the conclusions of Ref. [**?**].

TABLE I | Assessing CHGNet for determination of surface energy Comparison of DFT and CHGNet computed values of surface energy for interconnect candidates TiS and ZrB_2.

Compound/Surface	DFT (eV/$\mathring{A}^2$)	CHGNet (eV/$\mathring{A}^2$)
TiS/(001)	0.078	0.074
TiS/(100)	0.055	0.049
ZrB_2/(001)	0.193	0.142
ZrB_2/(100)	0.232	0.166

X. SUMMARY OF RESULTS

Compound: AgN_3
Materials Project ID: 571297

Lattice (conventional cell):

Parameter	Value	Unit
a	5.8273	Å
b	5.8273	Å
c	6.0257	Å
α (alpha)	90.0000	°
β (beta)	90.0000	°
γ (gamma)	90.0000	°

Crystal structure (conventional cell):

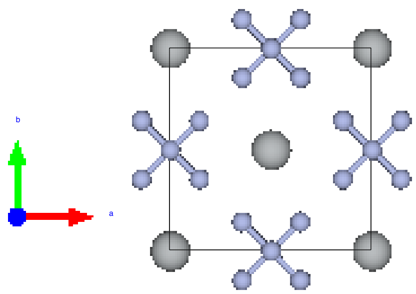

Nanowire Transmission:

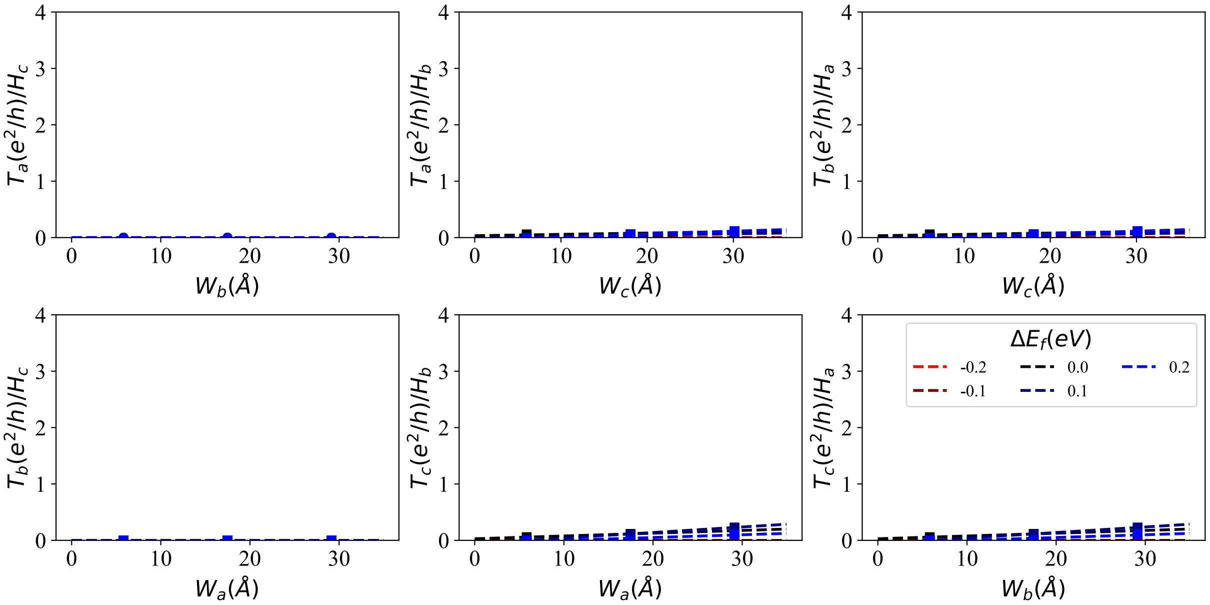

Compound: Ag_2Se
Materials Project ID: 568936

Lattice (conventional cell):

Parameter	Value	Unit
a	4.4975	Å
b	7.1133	Å
c	7.6707	Å
α (alpha)	90.0000	°
β (beta)	90.0000	°
γ (gamma)	90.0000	°

Crystal structure (conventional cell):

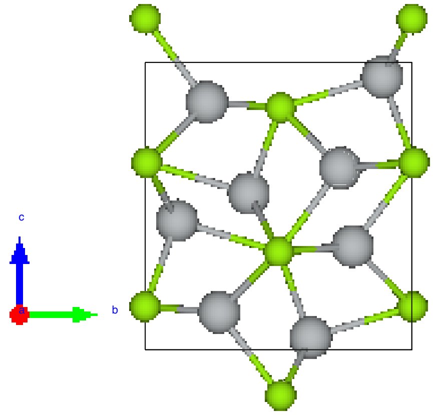

Nanowire Transmission:

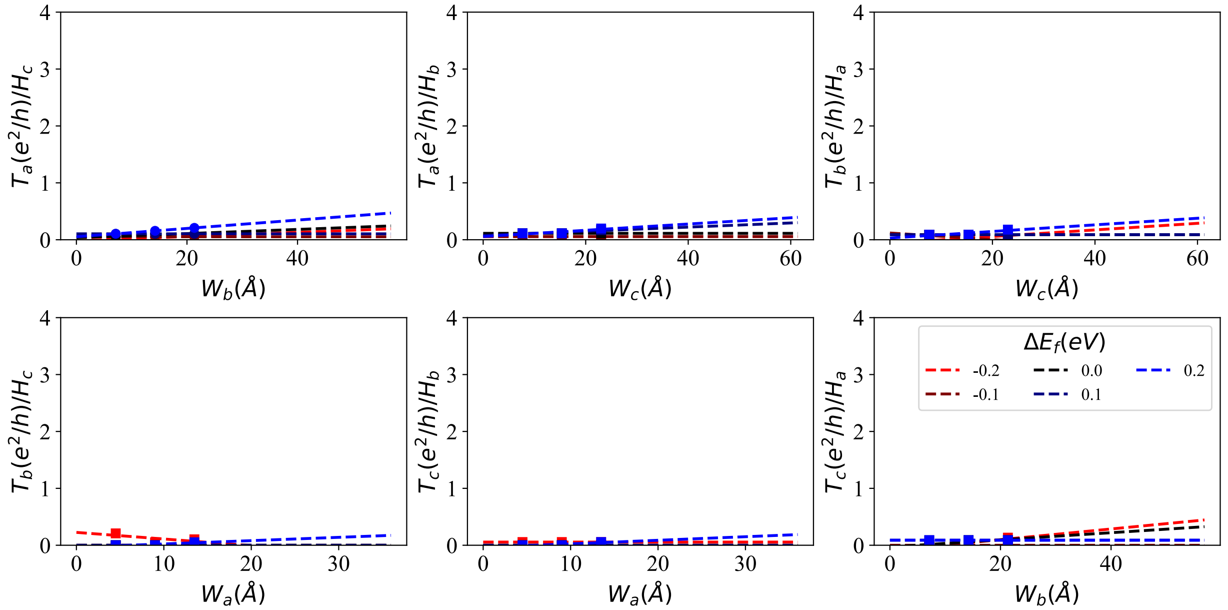

Compound: $AlPd_5I_2$
Materials Project ID: 27393

Lattice (conventional cell):

Parameter	Value	Unit
a	4.0783	Å
b	4.0783	Å
c	20.2662	Å
α (alpha)	90.0000	°
β (beta)	90.0000	°
γ (gamma)	90.0000	°

Crystal structure (conventional cell):

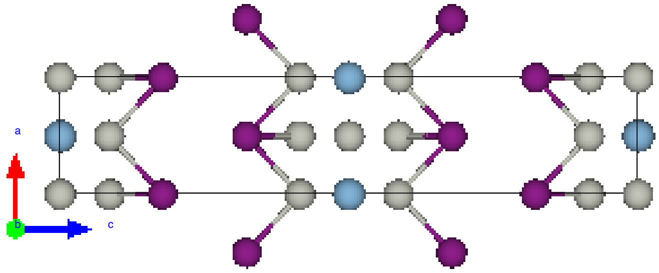

Nanowire Transmission:

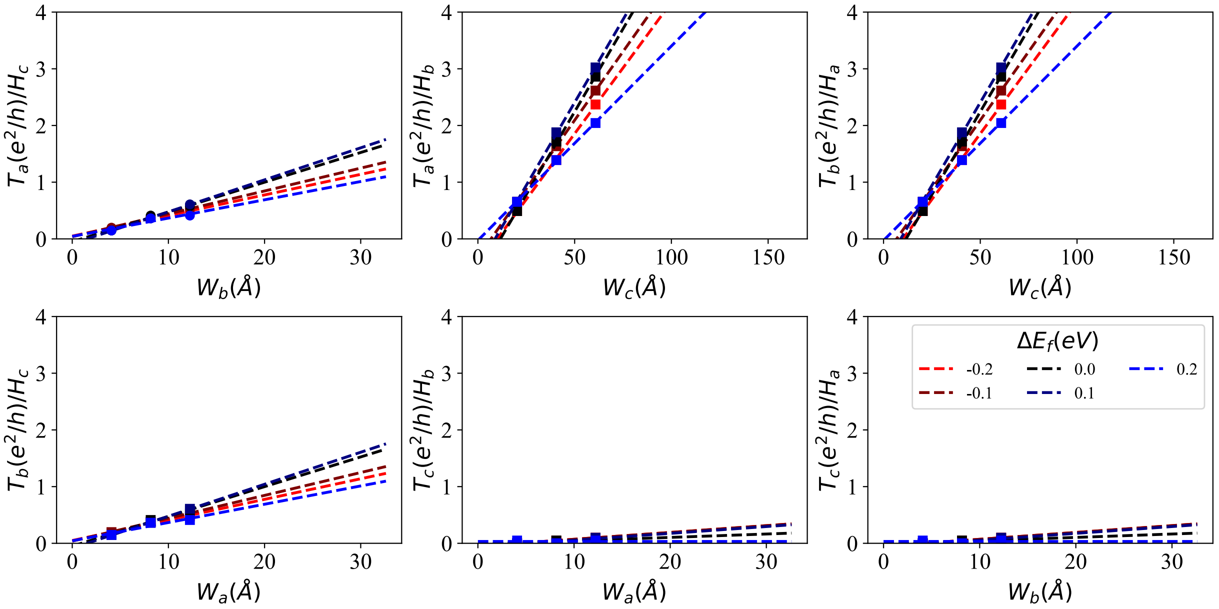

Compound: AlPt
Materials Project ID: 10904

Lattice (conventional cell):

Parameter	Value	Unit
a	4.9174	Å
b	4.9174	Å
c	4.9174	Å
α (alpha)	90.0000	°
β (beta)	90.0000	°
γ (gamma)	90.0000	°

Crystal structure (conventional cell):

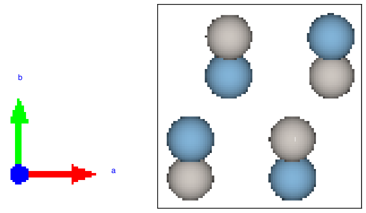

Nanowire Transmission:

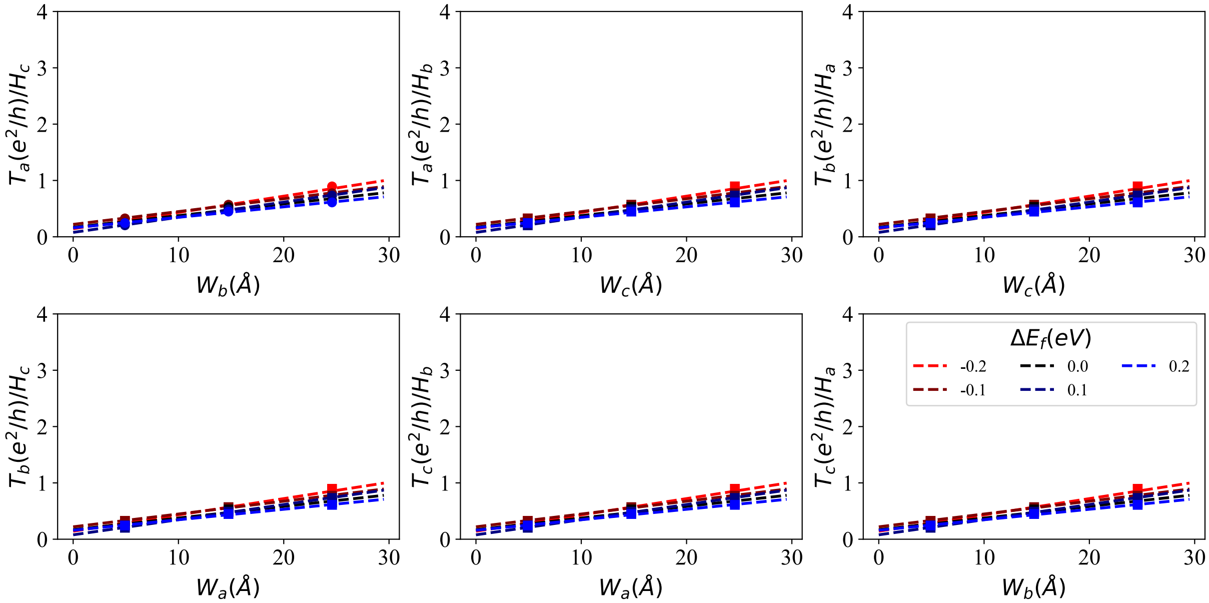

Compound: Au
Materials Project ID: 81

Lattice (conventional cell):

Parameter	Value	Unit
a	4.1713	Å
b	4.1713	Å
c	4.1713	Å
α (alpha)	90.0000	°
β (beta)	90.0000	°
γ (gamma)	90.0000	°

Crystal structure (conventional cell):

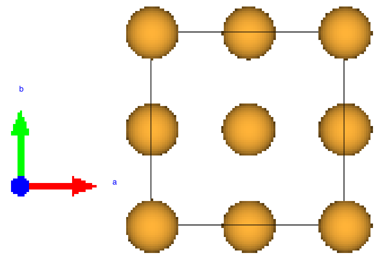

Nanowire Transmission:

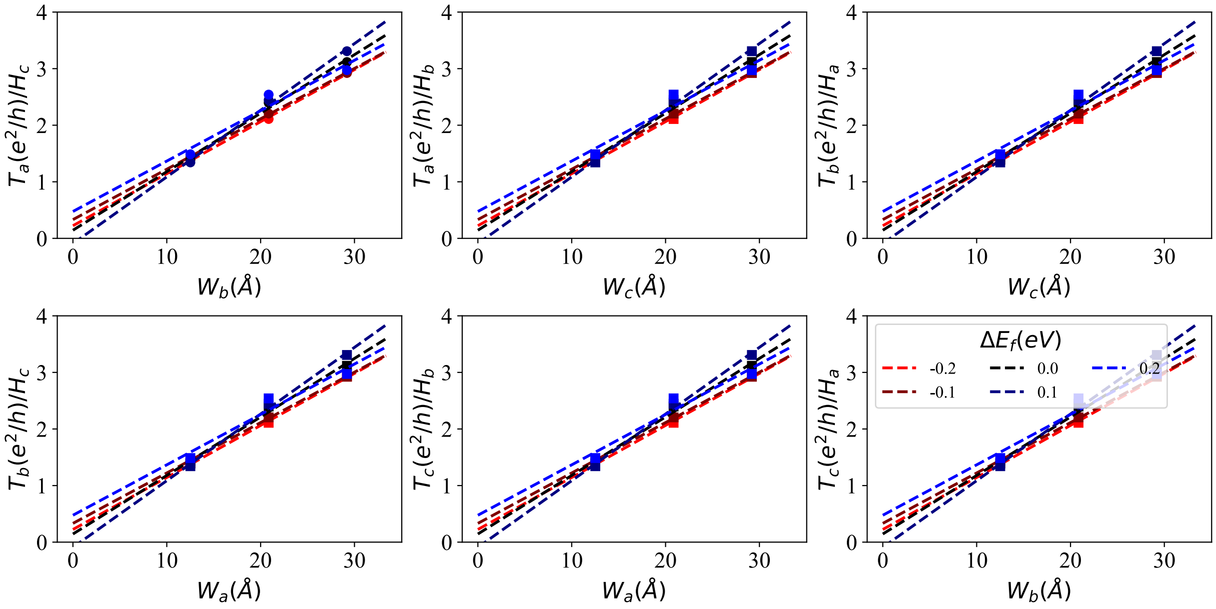

Compound: $BaAl_4$
Materials Project ID: 1903

Lattice (conventional cell):

Parameter	Value	Unit
a	4.5765	Å
b	4.5765	Å
c	11.3531	Å
α (alpha)	90.0000	°
β (beta)	90.0000	°
γ (gamma)	90.0000	°

Crystal structure (conventional cell):

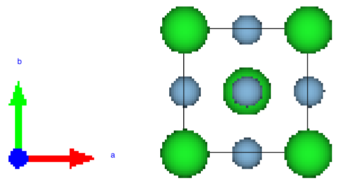

Nanowire Transmission:

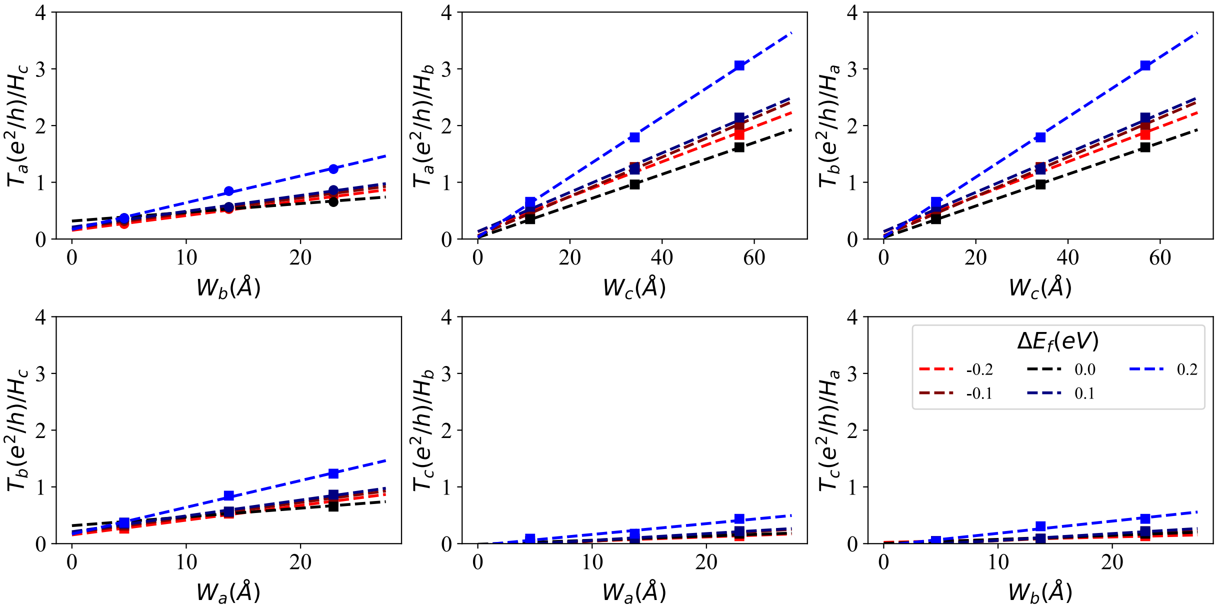

Compound: BaB_6
Materials Project ID: 954

Lattice (conventional cell):

Parameter	Value	Unit
a	4.2801	Å
b	4.2801	Å
c	4.2801	Å
α (alpha)	90.0000	°
β (beta)	90.0000	°
γ (gamma)	90.0000	°

Crystal structure (conventional cell):

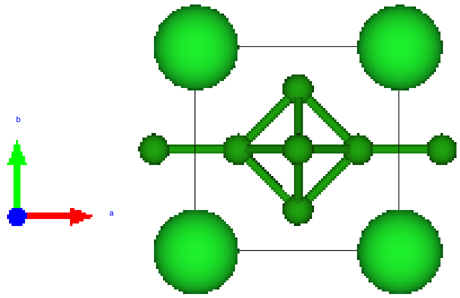

Nanowire Transmission:

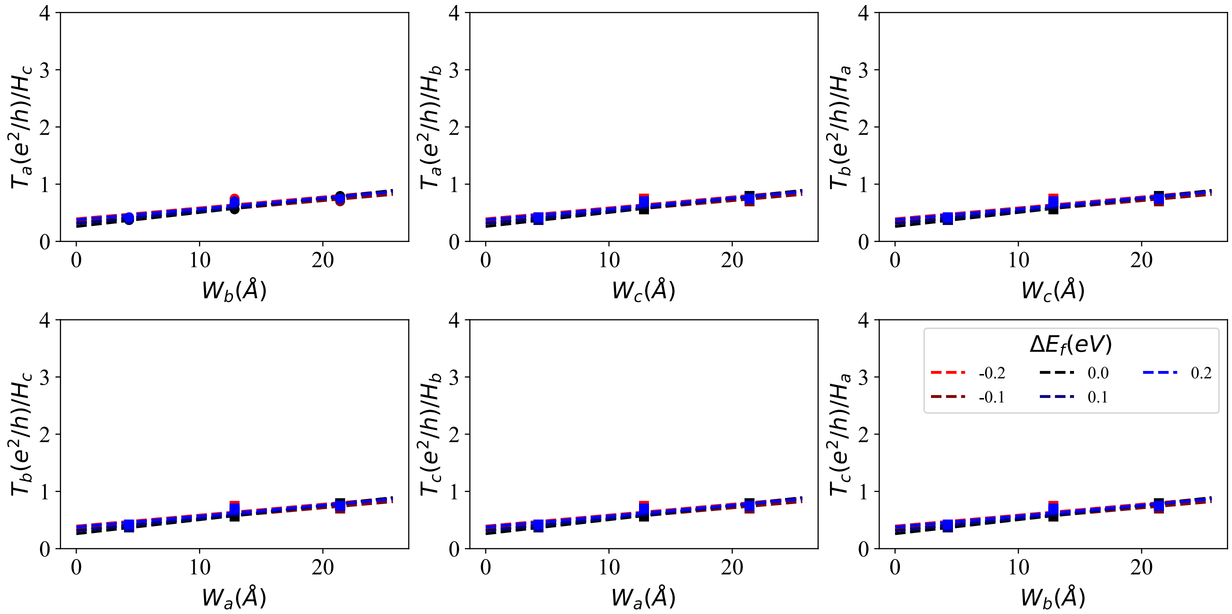

Compound: $BaSi_2$
Materials Project ID: 7655

Lattice (conventional cell):

Parameter	Value	Unit
a	4.0928	Å
b	4.0928	Å
c	5.3629	Å
α (alpha)	90.0000	°
β (beta)	90.0000	°
γ (gamma)	120.0000	°

Crystal structure (conventional cell):

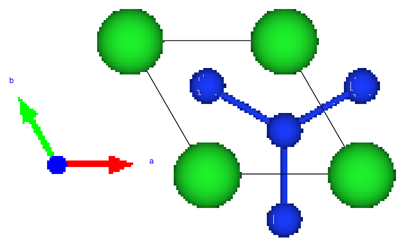

Nanowire Transmission:

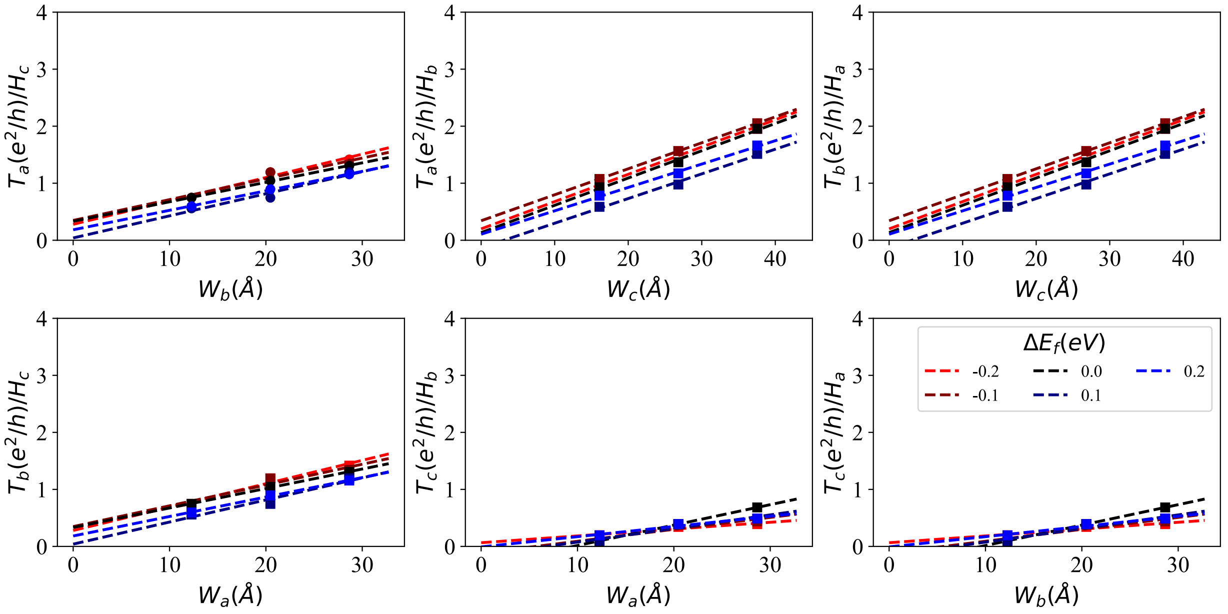

Compound: $BaSn_2$
Materials Project ID: 567510

Lattice (conventional cell):

Parameter	Value	Unit
a	4.7650	Å
b	4.7650	Å
c	5.6084	Å
α (alpha)	90.0000	°
β (beta)	90.0000	°
γ (gamma)	120.0000	°

Crystal structure (conventional cell):

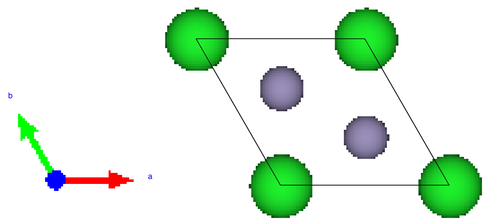

Nanowire Transmission:

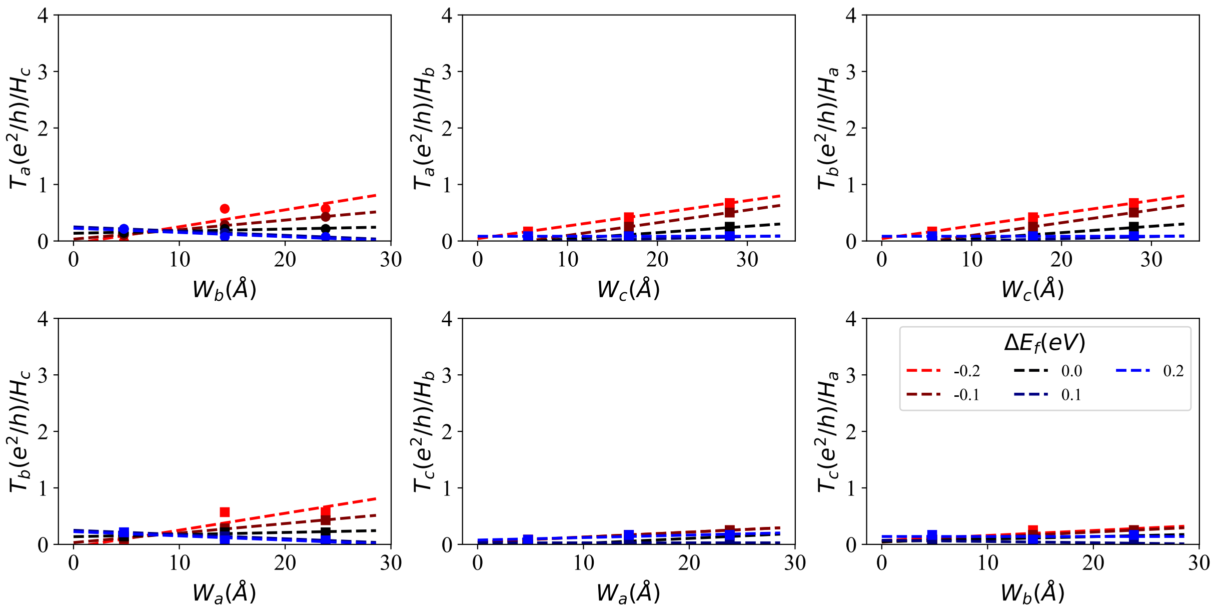

Compound: Be
Materials Project ID: 87

Lattice (conventional cell):

Parameter	Value	Unit
a	2.2598	Å
b	2.2598	Å
c	3.5699	Å
α (alpha)	90.0000	°
β (beta)	90.0000	°
γ (gamma)	120.0000	°

Crystal structure (conventional cell):

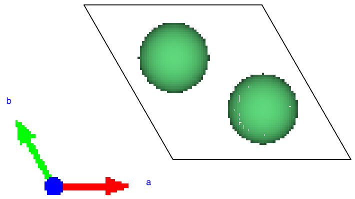

Nanowire Transmission:

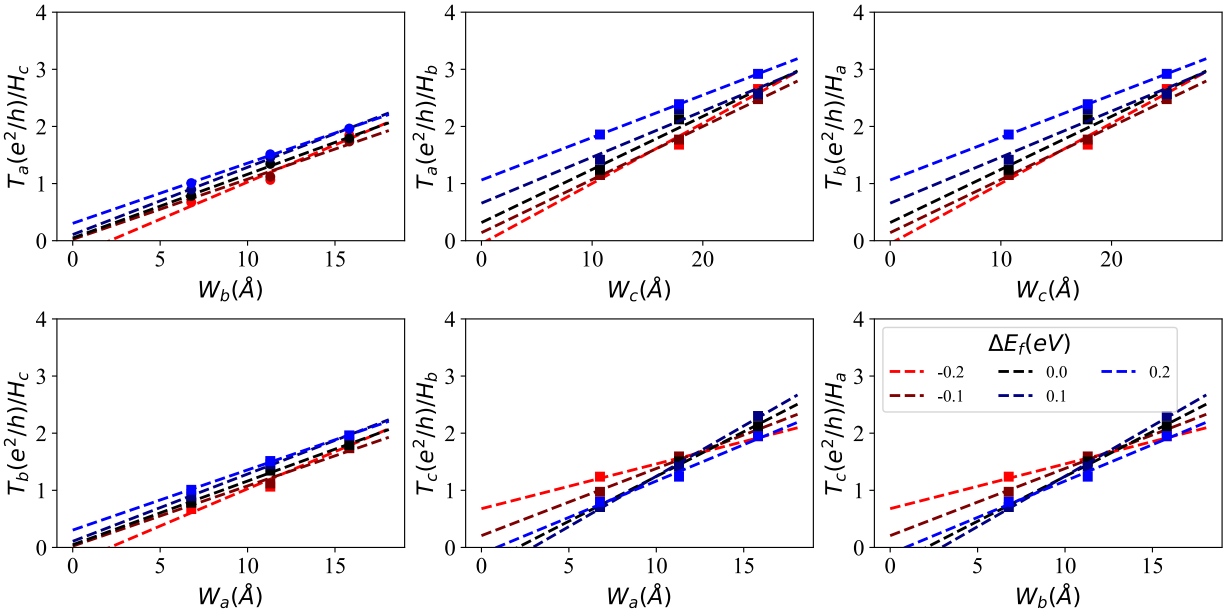

Compound: Bi
Materials Project ID: 23152

Lattice (conventional cell):

Parameter	Value	Unit
a	4.6096	Å
b	4.6096	Å
c	11.9755	Å
α (alpha)	90.0000	°
β (beta)	90.0000	°
γ (gamma)	120.0000	°

Crystal structure (conventional cell):

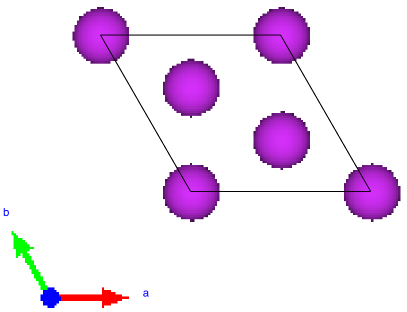

Nanowire Transmission:

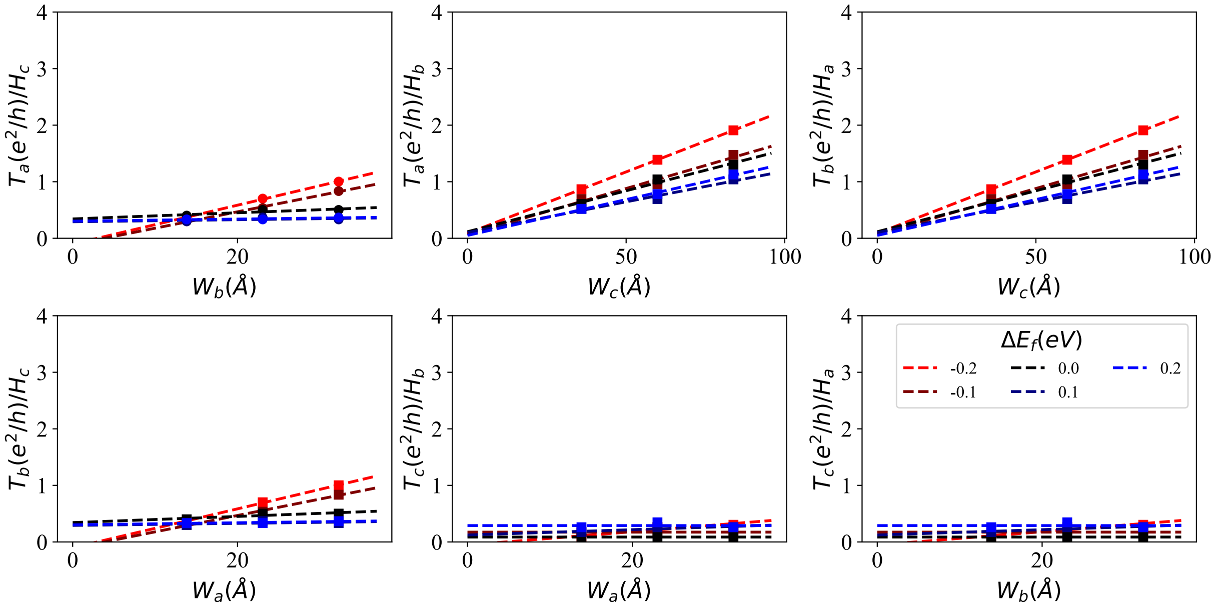

Compound: BiTeI
Materials Project ID: 22965

Lattice (conventional cell):

Parameter	Value	Unit
a	4.4252	Å
b	4.4252	Å
c	7.3781	Å
α (alpha)	90.0000	°
β (beta)	90.0000	°
γ (gamma)	120.0000	°

Crystal structure (conventional cell):

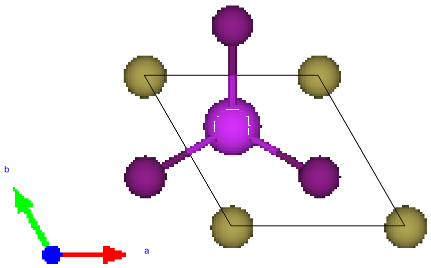

Nanowire Transmission:

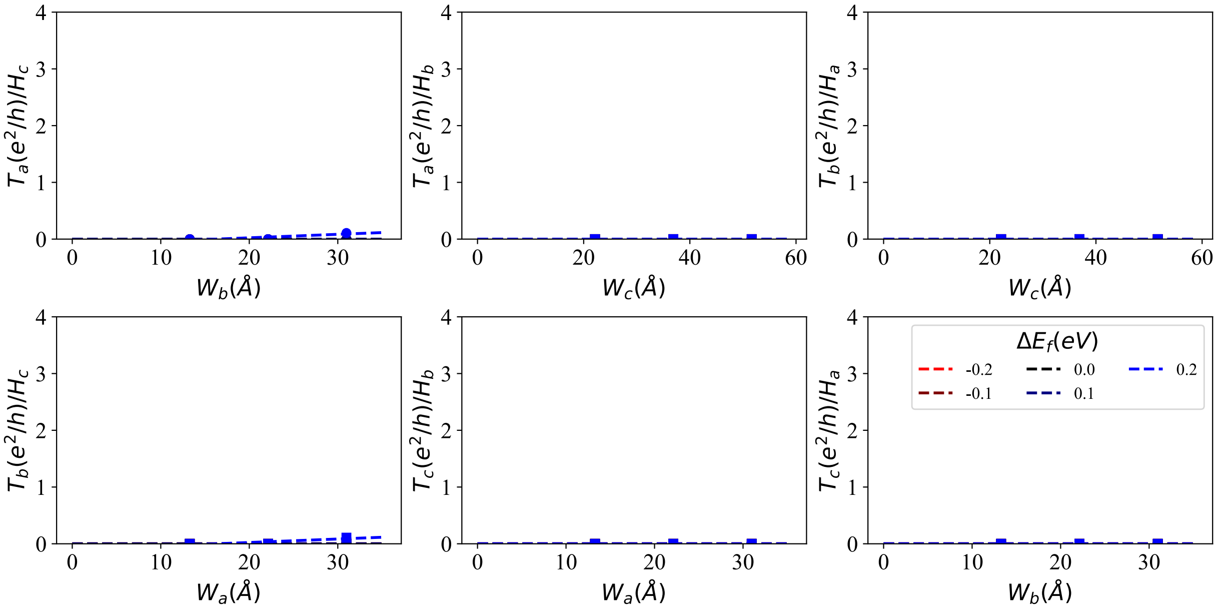

Compound: Ca_2As
Materials Project ID: 10106

Lattice (conventional cell):

Parameter	Value	Unit
a	4.5724	Å
b	4.5724	Å
c	15.8762	Å
α (alpha)	90.0000	°
β (beta)	90.0000	°
γ (gamma)	90.0000	°

Crystal structure (conventional cell):

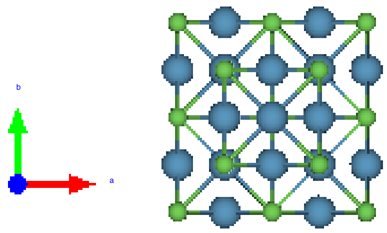

Nanowire Transmission:

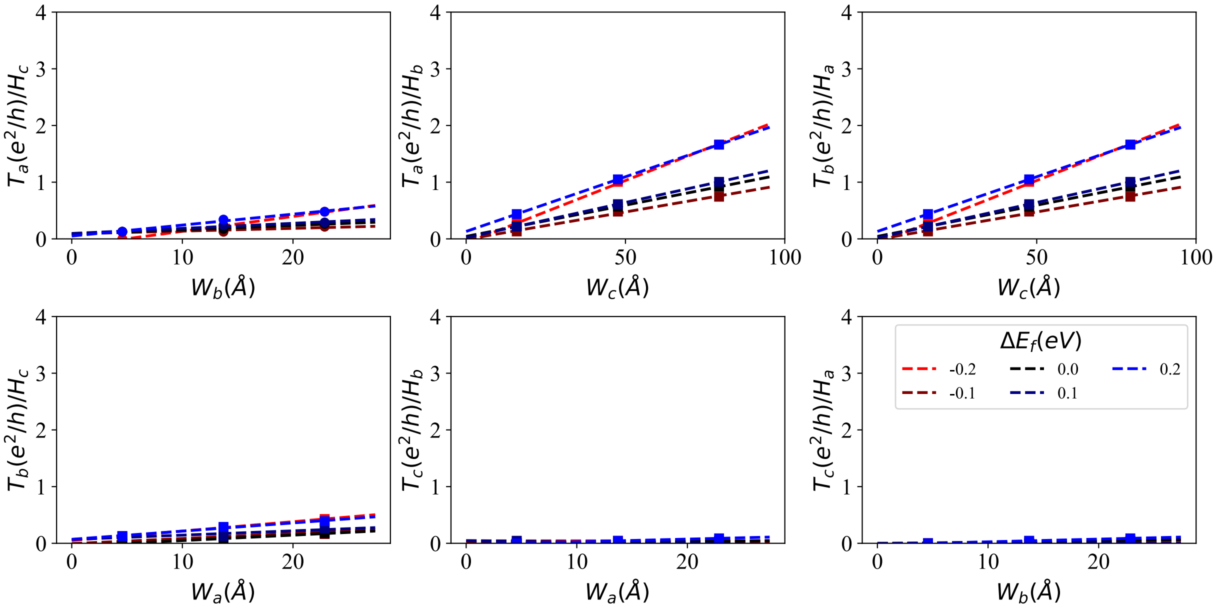

Compound: CaAgAs
Materials Project ID: 5615

Lattice (conventional cell):

Parameter	Value	Unit
a	7.2663	Å
b	7.2663	Å
c	4.3127	Å
α (alpha)	90.0000	°
β (beta)	90.0000	°
γ (gamma)	120.0000	°

Crystal structure (conventional cell):

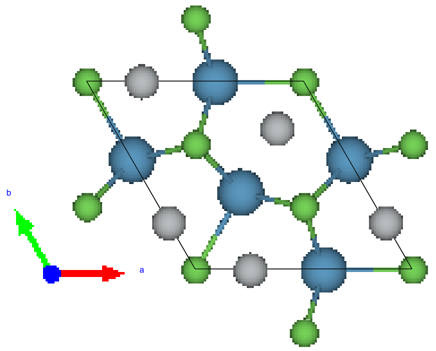

Nanowire Transmission:

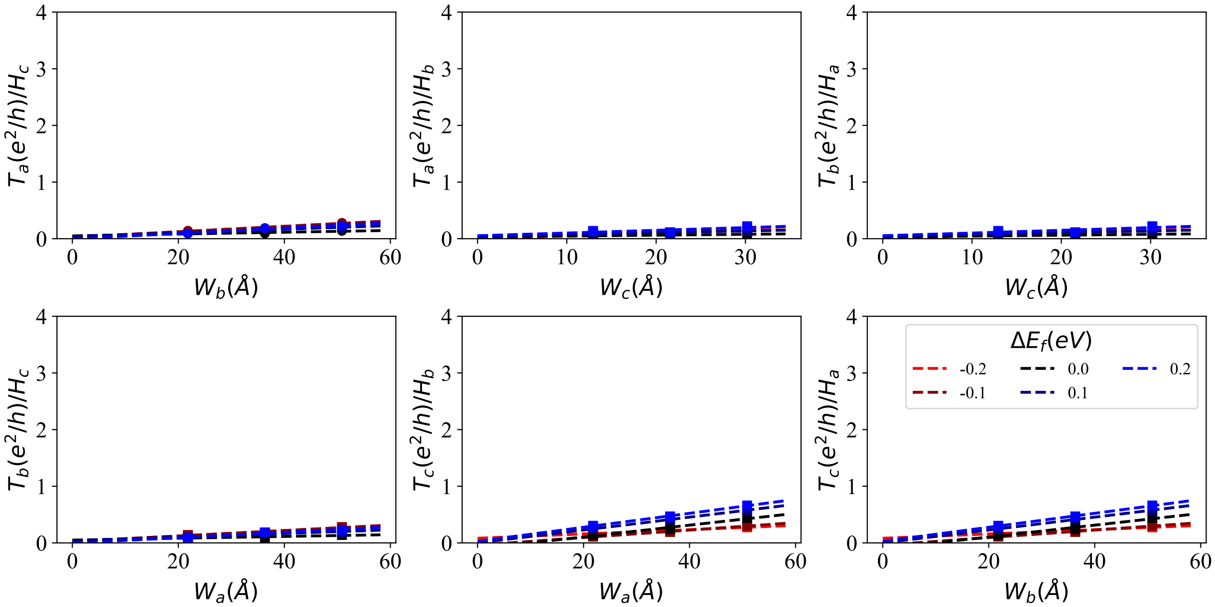

Compound: CaAgBi
Materials Project ID: 568664

Lattice (conventional cell):

Parameter	Value	Unit
a	4.9065	Å
b	4.9065	Å
c	7.8429	Å
α (alpha)	90.0000	°
β (beta)	90.0000	°
γ (gamma)	120.0000	°

Crystal structure (conventional cell):

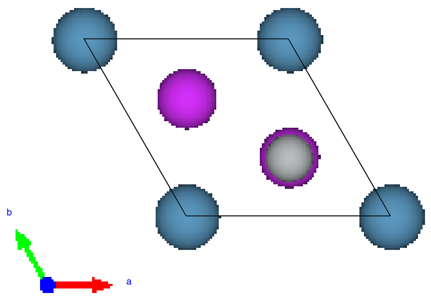

Nanowire Transmission:

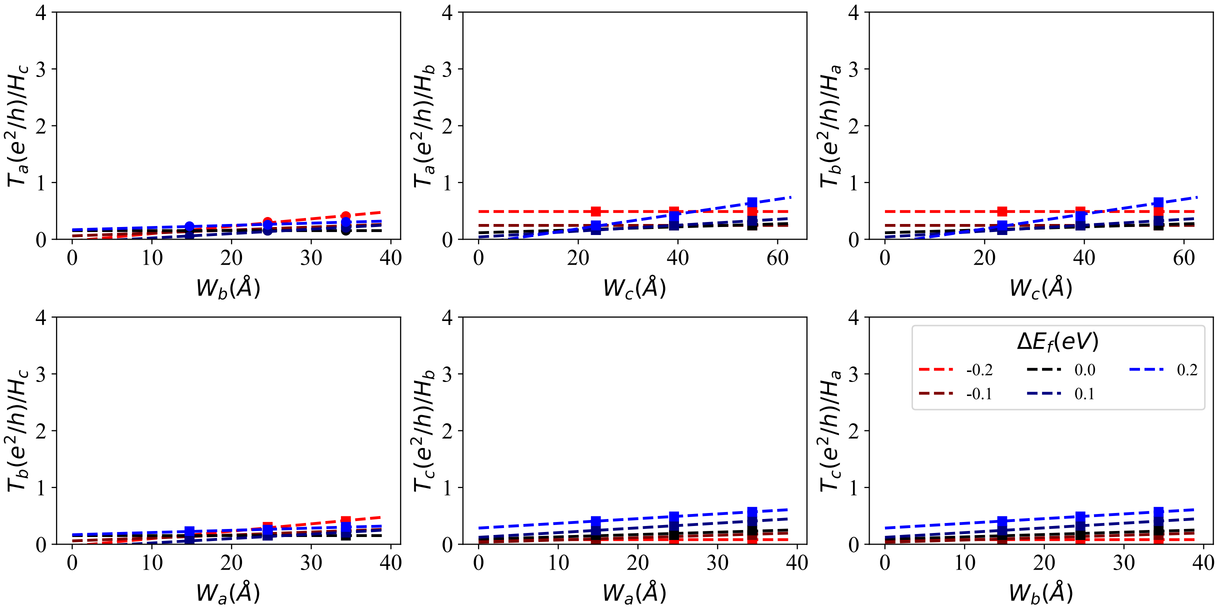

Compound: CaAgP
Materials Project ID: 12277

Lattice (conventional cell):

Parameter	Value	Unit
a	7.0768	Å
b	7.0768	Å
c	4.2030	Å
α (alpha)	90.0000	°
β (beta)	90.0000	°
γ (gamma)	120.0000	°

Crystal structure (conventional cell):

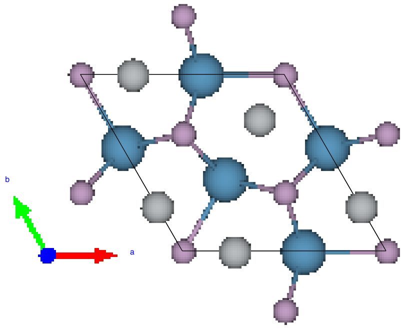

Nanowire Transmission:

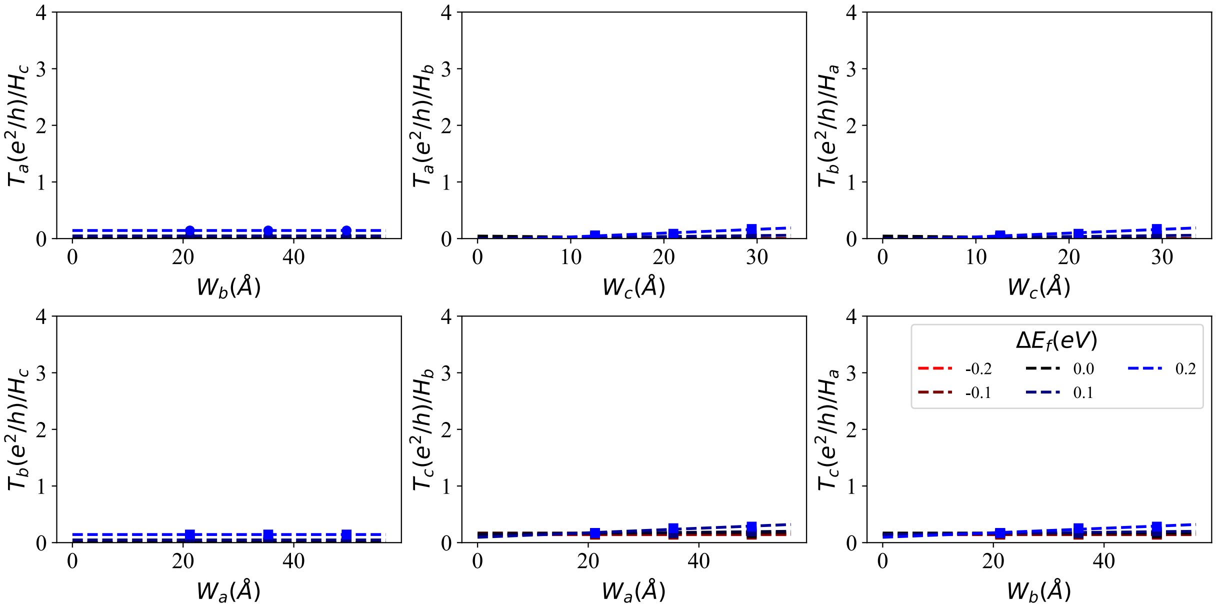

Compound: CaB_6
Materials Project ID: 865

Lattice (conventional cell):

Parameter	Value	Unit
a	4.1501	Å
b	4.1501	Å
c	4.1501	Å
α (alpha)	90.0000	°
β (beta)	90.0000	°
γ (gamma)	90.0000	°

Crystal structure (conventional cell):

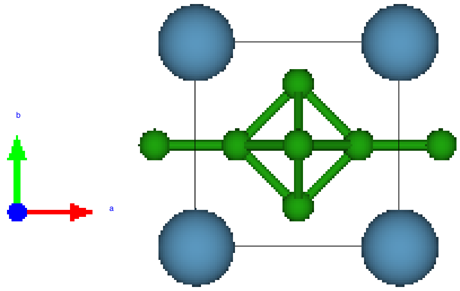

Nanowire Transmission:

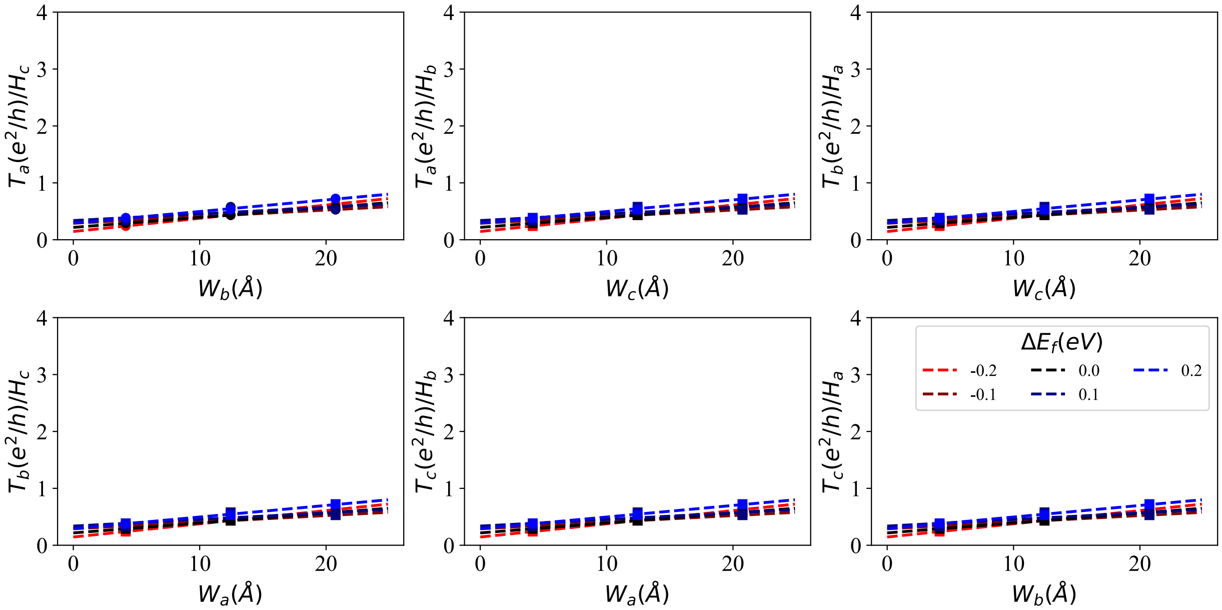

Compound: CaBiAu
Materials Project ID: 1018657

Lattice (conventional cell):

Parameter	Value	Unit
a	4.8612	Å
b	4.8612	Å
c	7.9224	Å
α (alpha)	90.0000	°
β (beta)	90.0000	°
γ (gamma)	120.0000	°

Crystal structure (conventional cell):

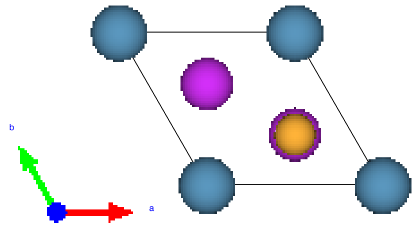

Nanowire Transmission:

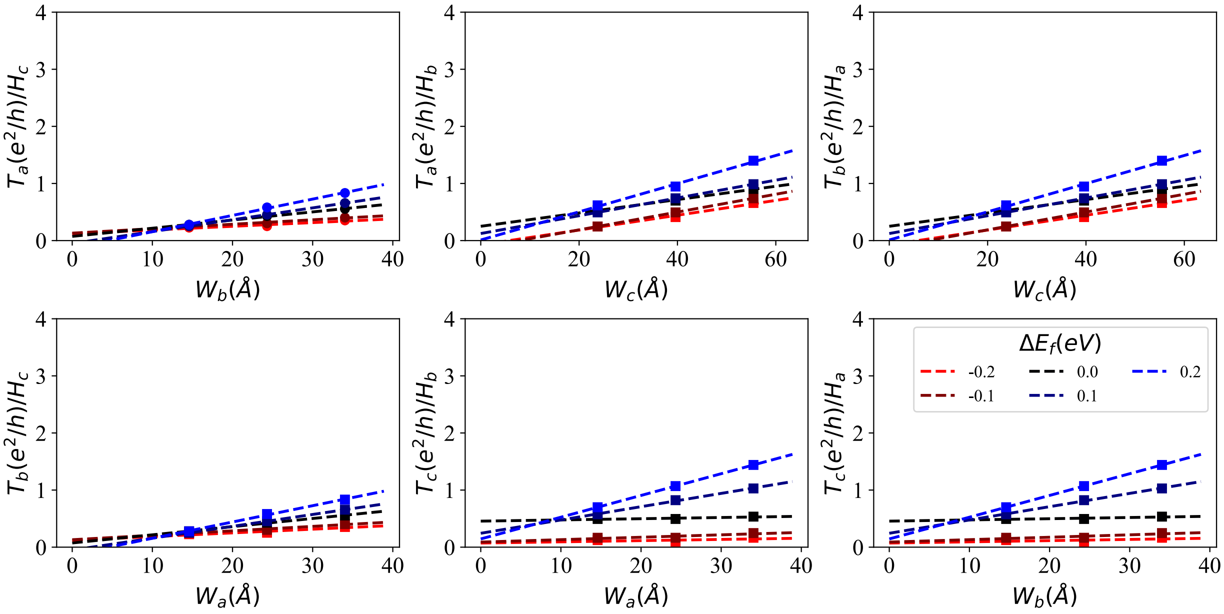

Compound: CaC_2
Materials Project ID: 1077545

Lattice (conventional cell):

Parameter	Value	Unit
a	3.7480	Å
b	8.7387	Å
c	4.7766	Å
α (alpha)	90.0000	°
β (beta)	90.0000	°
γ (gamma)	90.0000	°

Crystal structure (conventional cell):

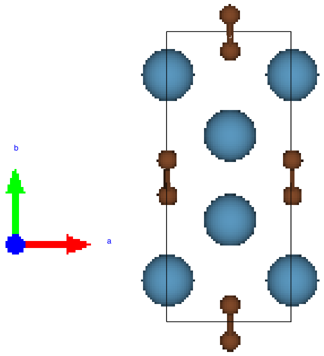

Nanowire Transmission:

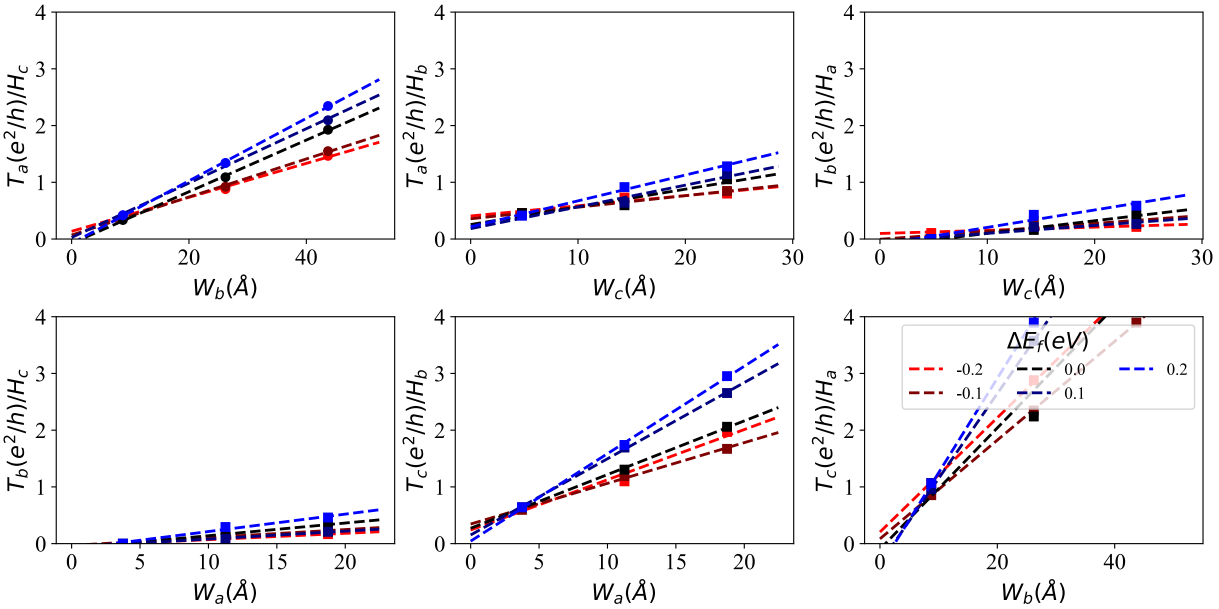

Compound: $CaSi_2$
Materials Project ID: 8372

Lattice (conventional cell):

Parameter	Value	Unit
a	3.8878	Å
b	3.8878	Å
c	4.9460	Å
α (alpha)	90.0000	°
β (beta)	90.0000	°
γ (gamma)	120.0000	°

Crystal structure (conventional cell):

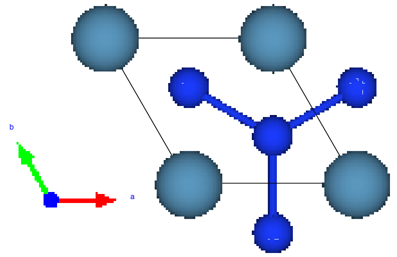

Nanowire Transmission:

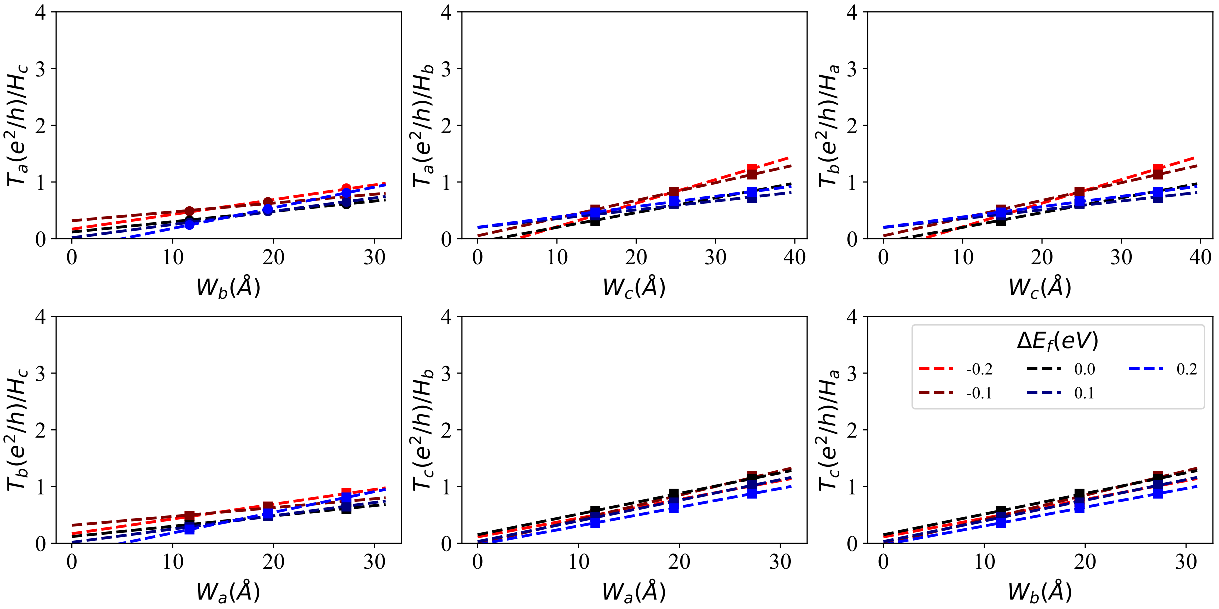

Compound: CaSnHg
Materials Project ID: 1019104

Lattice (conventional cell):

Parameter	Value	Unit
a	4.9042	Å
b	4.9042	Å
c	7.8216	Å
α (alpha)	90.0000	°
β (beta)	90.0000	°
γ (gamma)	120.0000	°

Crystal structure (conventional cell):

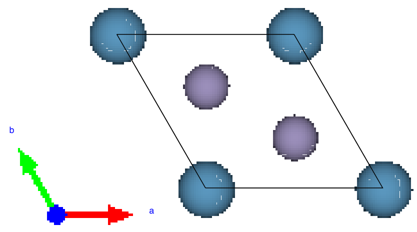

Nanowire Transmission:

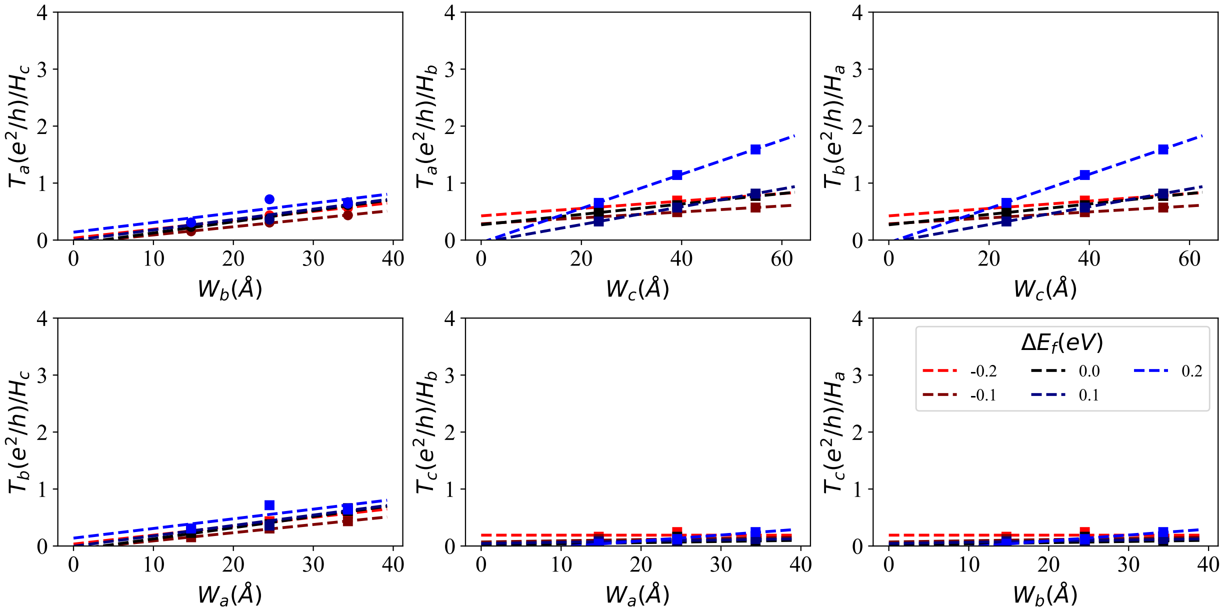

Compound: CaTe
Materials Project ID: 10684

Lattice (conventional cell):

Parameter	Value	Unit
a	3.9261	Å
b	3.9261	Å
c	3.9261	Å
α (alpha)	90.0000	°
β (beta)	90.0000	°
γ (gamma)	90.0000	°

Crystal structure (conventional cell):

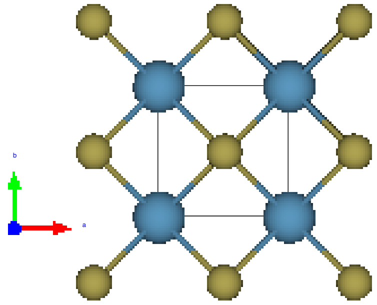

Nanowire Transmission:

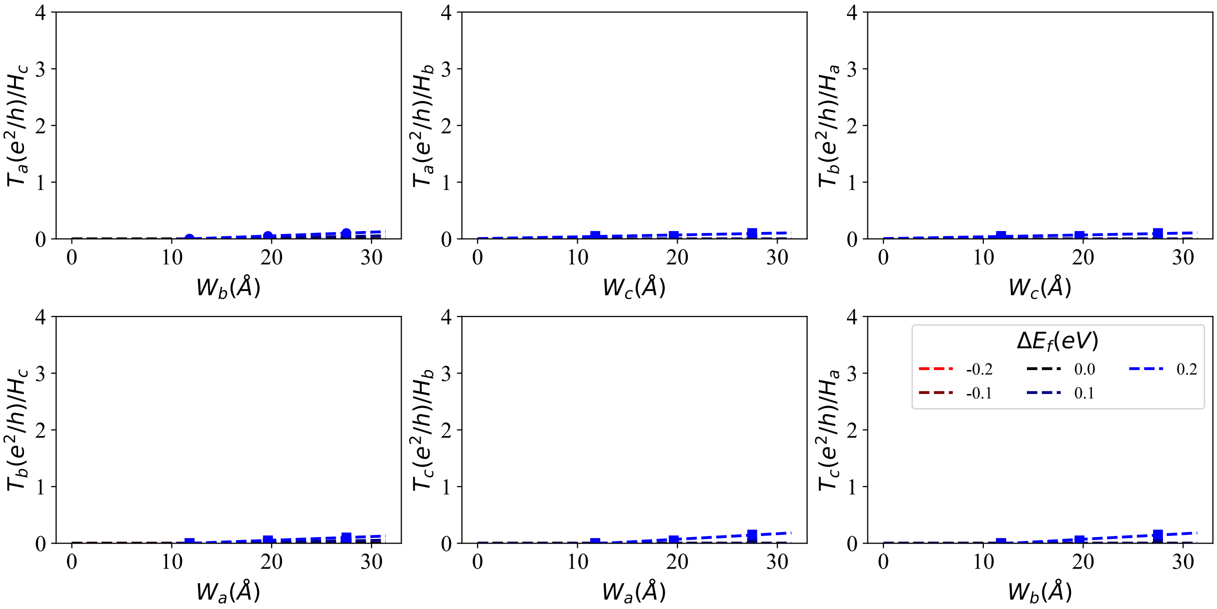

Compound: CaP_3
Materials Project ID: 9122

Lattice (conventional cell):

Parameter	Value	Unit
a	5.6095	Å
b	5.6390	Å
c	5.6946	Å
α (alpha)	70.0761	°
β (beta)	79.6272	°
γ (gamma)	74.6843	°

Crystal structure (conventional cell):

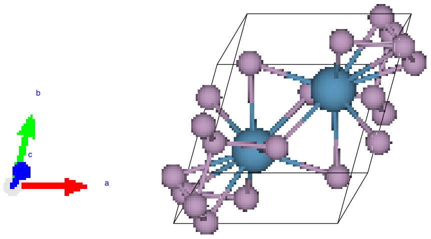

Nanowire Transmission:

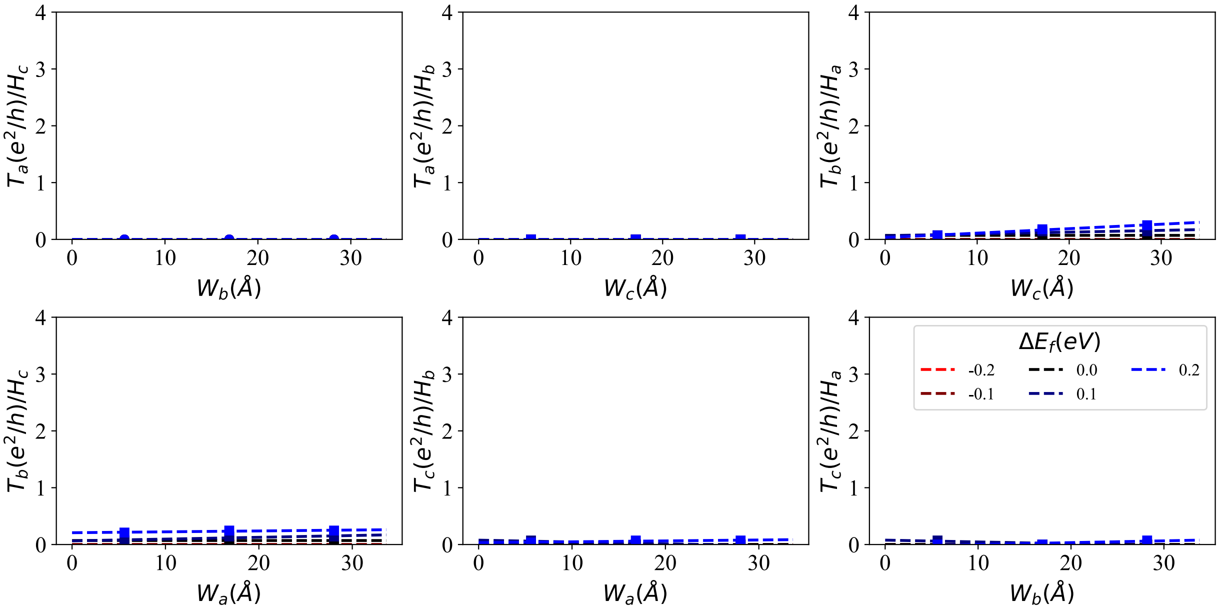

Compound: Co
Materials Project ID: 54

Lattice (conventional cell):

Parameter	Value	Unit
a	2.5008	Å
b	2.5008	Å
c	4.0333	Å
α (alpha)	90.0000	°
β (beta)	90.0000	°
γ (gamma)	120.0000	°

Crystal structure (conventional cell):

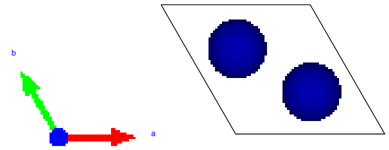

Nanowire Transmission:

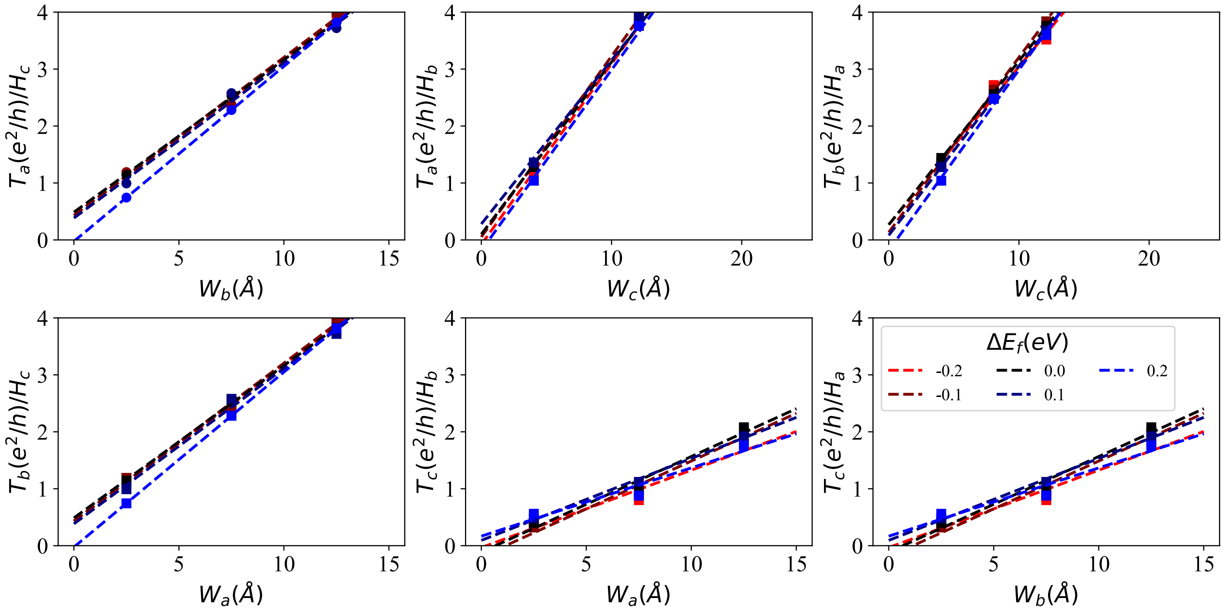

Compound: CoGe
Materials Project ID: 10692

Lattice (conventional cell):

Parameter	Value	Unit
a	4.6370	Å
b	4.6370	Å
c	4.6370	Å
α (alpha)	90.0000	°
β (beta)	90.0000	°
γ (gamma)	90.0000	°

Crystal structure (conventional cell):

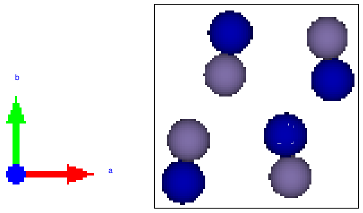

Nanowire Transmission:

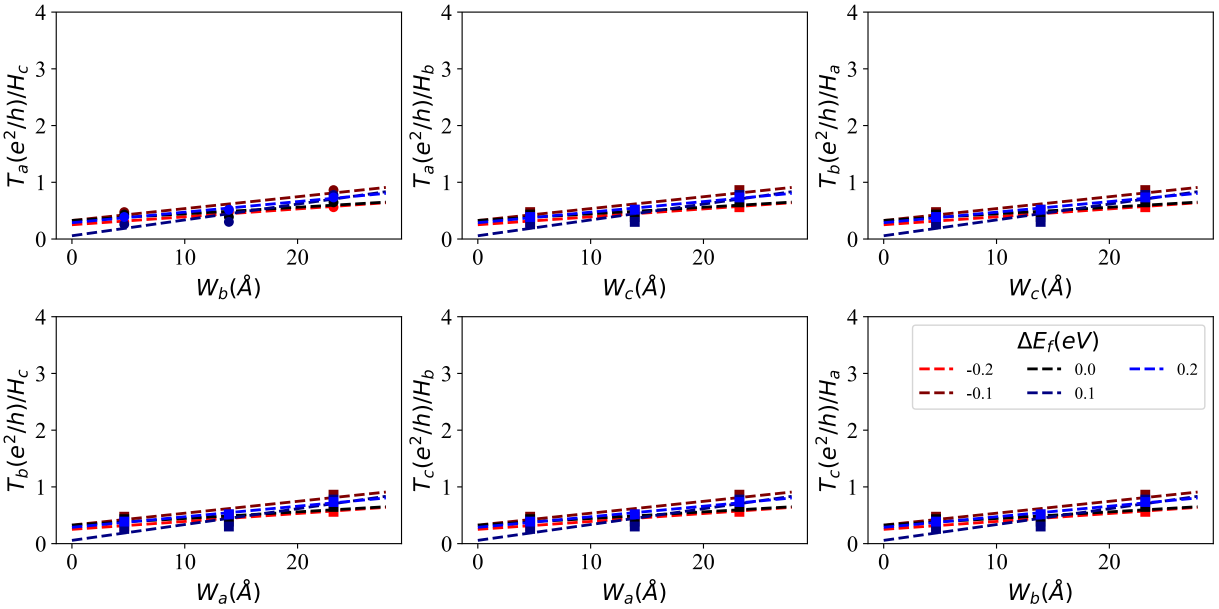

Compound: CoSi
Materials Project ID: 7577

Lattice (conventional cell):

Parameter	Value	Unit
a	4.4332	Å
b	4.4332	Å
c	4.4332	Å
α (alpha)	90.0000	°
β (beta)	90.0000	°
γ (gamma)	90.0000	°

Crystal structure (conventional cell):

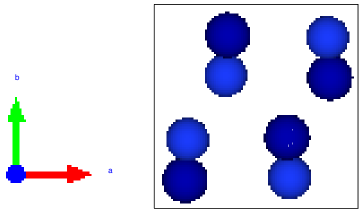

Nanowire Transmission:

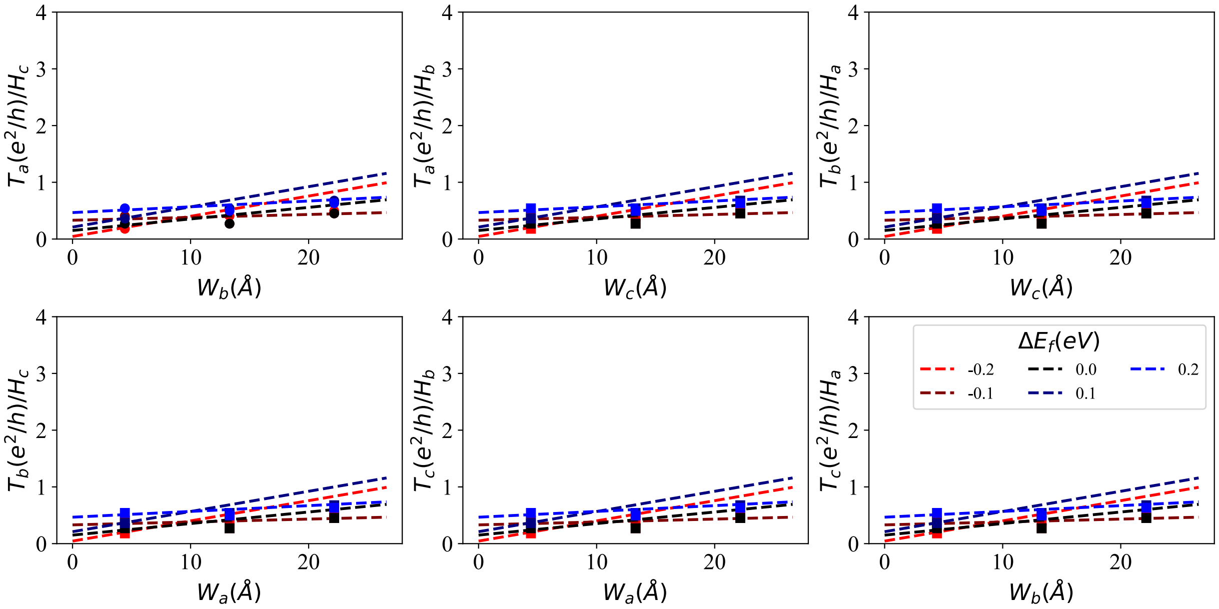

Compound: $CoPtO_2$
Materials Project ID: 19210

Lattice (conventional cell):

Parameter	Value	Unit
a	2.8847	Å
b	2.8847	Å
c	18.2033	Å
α (alpha)	90.0000	°
β (beta)	90.0000	°
γ (gamma)	120.0000	°

Crystal structure (conventional cell):

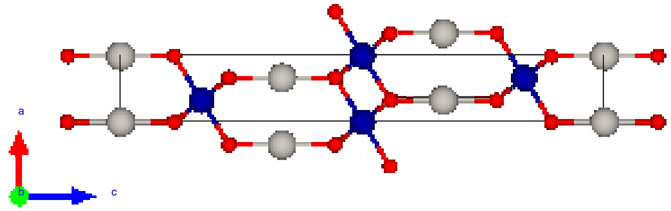

Nanowire Transmission:

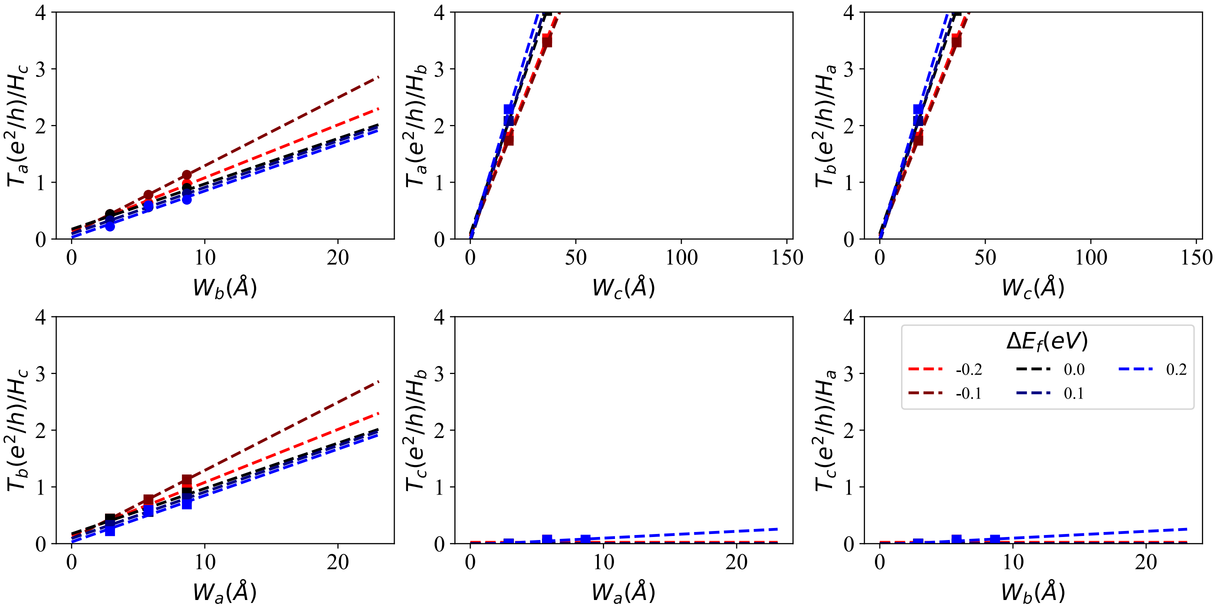

Compound: Cu
Materials Project ID: 30

Lattice (conventional cell):

Parameter	Value	Unit
a	3.6213	Å
b	3.6213	Å
c	3.6213	Å
α (alpha)	90.0000	°
β (beta)	90.0000	°
γ (gamma)	90.0000	°

Crystal structure (conventional cell):

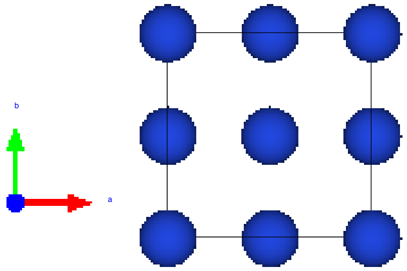

Nanowire Transmission:

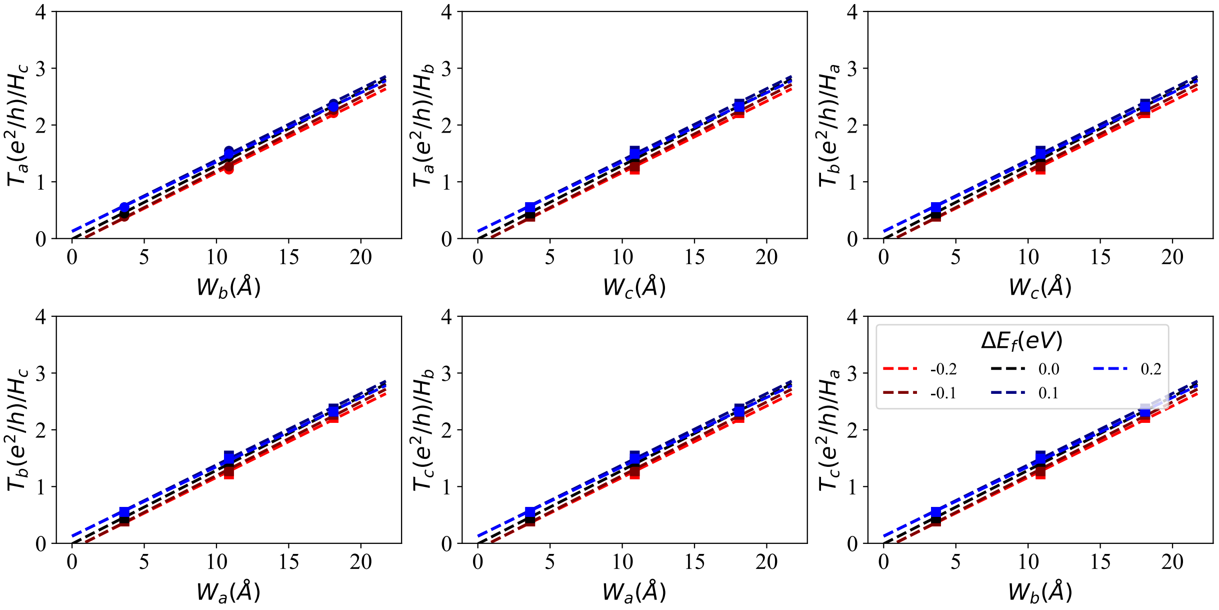

Compound: Cu_3AsSe_4
Materials Project ID: 675626

Lattice (conventional cell):

Parameter	Value	Unit
a	5.6212	Å
b	5.6212	Å
c	11.1400	Å
α (alpha)	90.0000	°
β (beta)	90.0000	°
γ (gamma)	90.0000	°

Crystal structure (conventional cell):

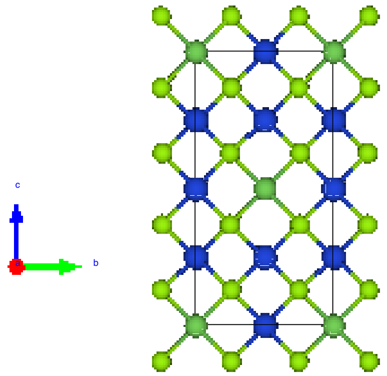

Nanowire Transmission:

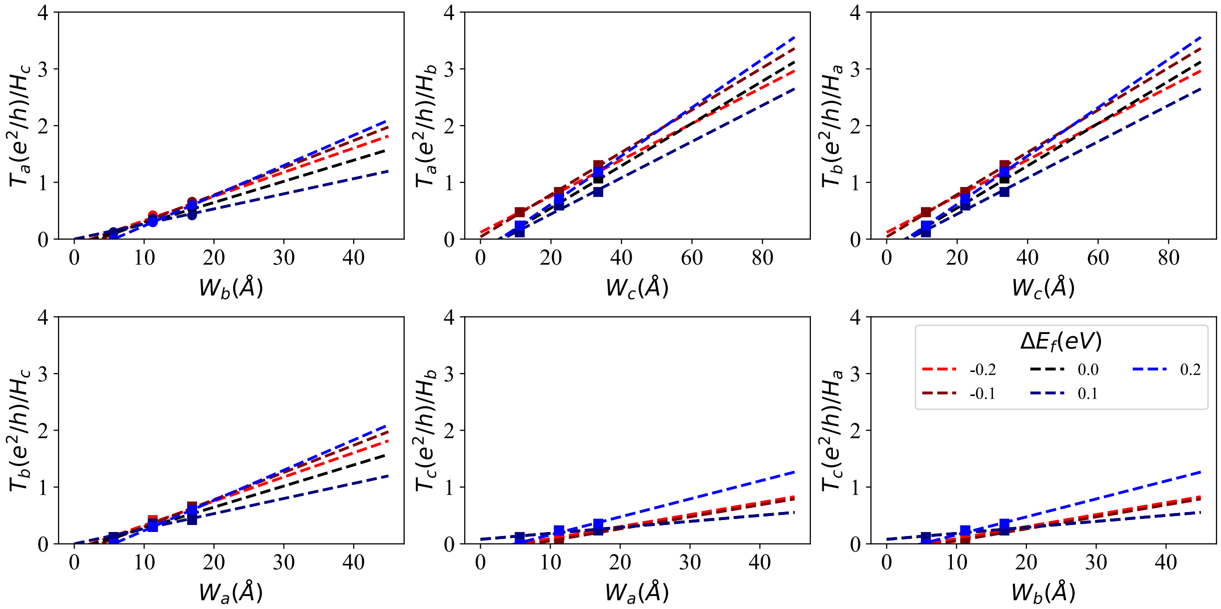

Compound: GaPd
Materials Project ID: 1078526

Lattice (conventional cell):

Parameter	Value	Unit
a	4.9634	Å
b	4.9634	Å
c	4.9634	Å
α (alpha)	90.0000	°
β (beta)	90.0000	°
γ (gamma)	90.0000	°

Crystal structure (conventional cell):

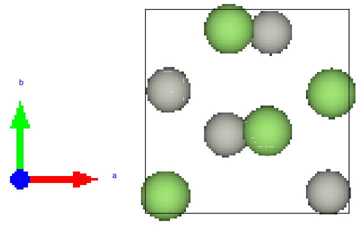

Nanowire Transmission:

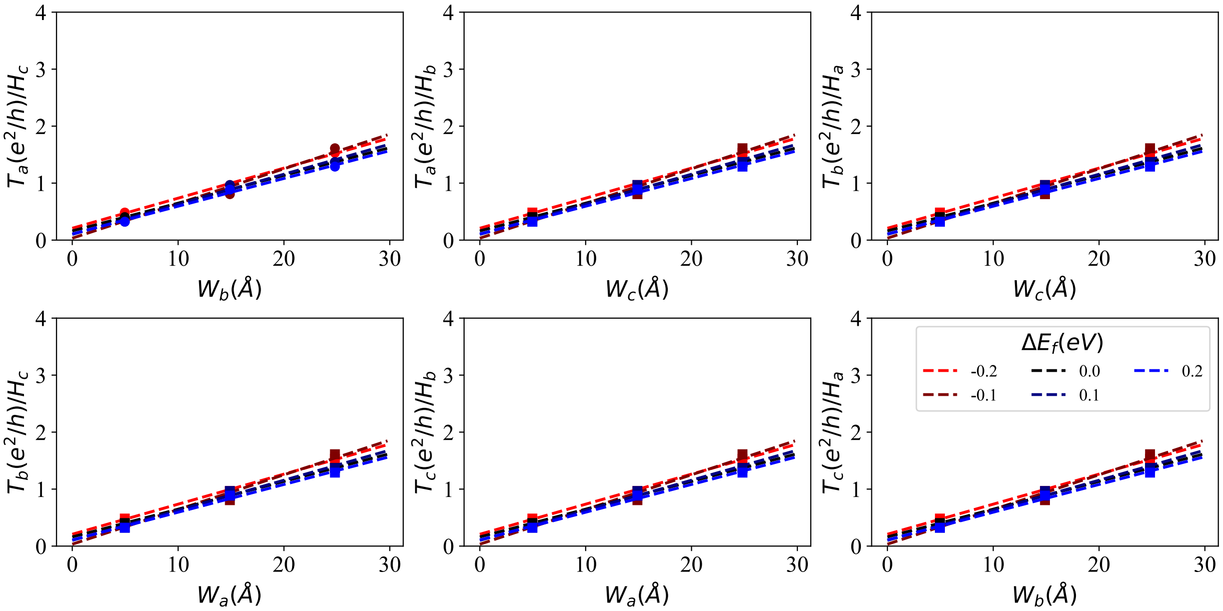

Compound: GaPt
Materials Project ID: 1025551

Lattice (conventional cell):

Parameter	Value	Unit
a	4.9729	Å
b	4.9729	Å
c	4.9729	Å
α (alpha)	90.0000	°
β (beta)	90.0000	°
γ (gamma)	90.0000	°

Crystal structure (conventional cell):

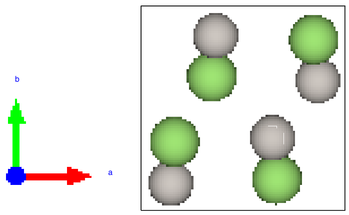

Nanowire Transmission:

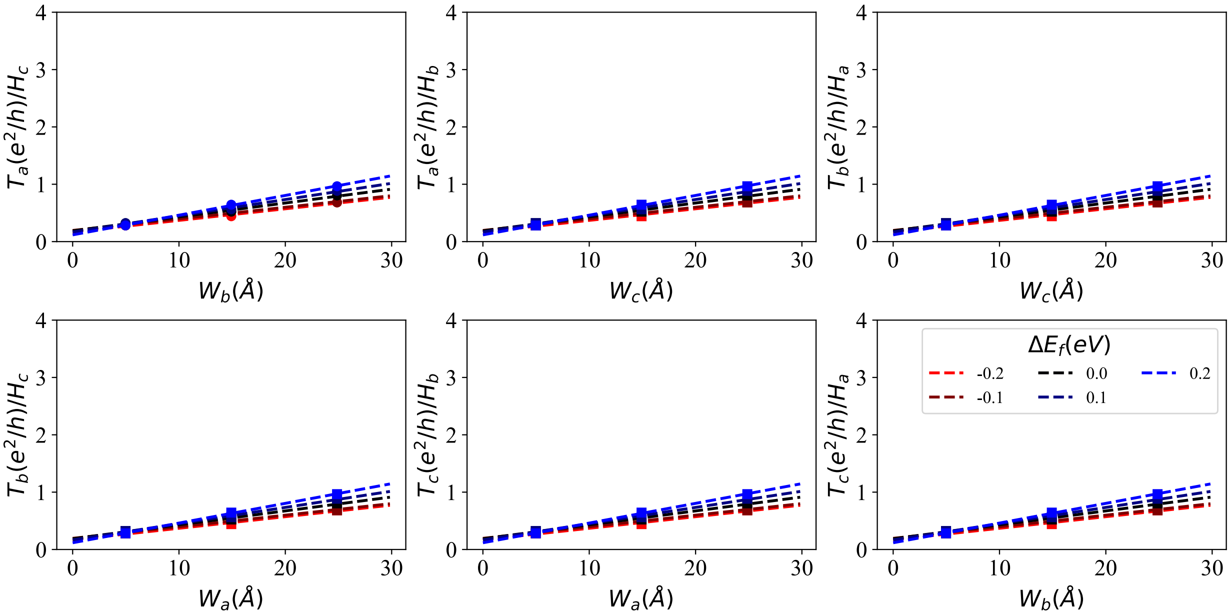

Compound: GaGeTe
Materials Project ID: 8211

Lattice (conventional cell):

Parameter	Value	Unit
a	4.1264	Å
b	4.1264	Å
c	36.5601	Å
α (alpha)	90.0000	°
β (beta)	90.0000	°
γ (gamma)	120.0000	°

Crystal structure (conventional cell):

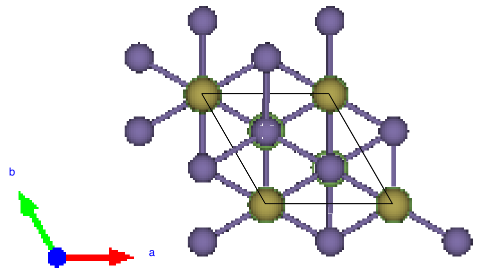

Nanowire Transmission:

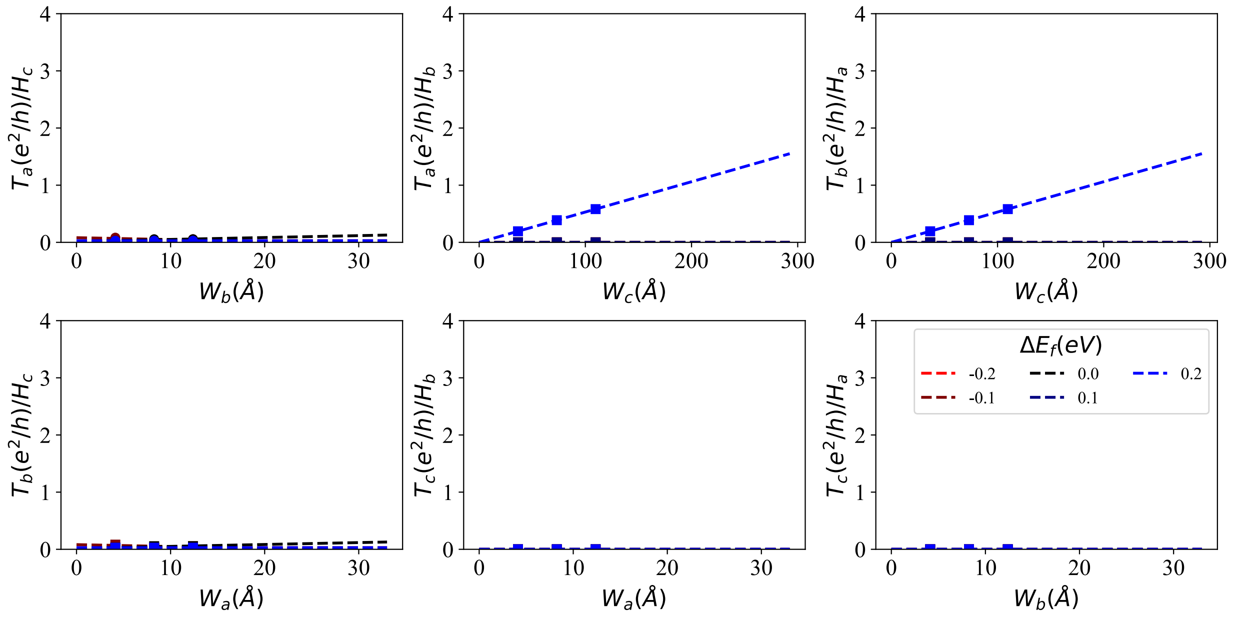

Compound: GeRh
Materials Project ID: 20866

Lattice (conventional cell):

Parameter	Value	Unit
a	4.8620	Å
b	4.8620	Å
c	4.8620	Å
α (alpha)	90.0000	°
β (beta)	90.0000	°
γ (gamma)	90.0000	°

Crystal structure (conventional cell):

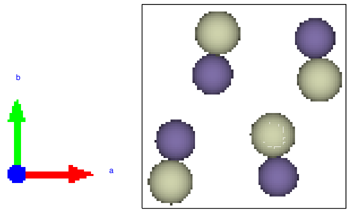

Nanowire Transmission:

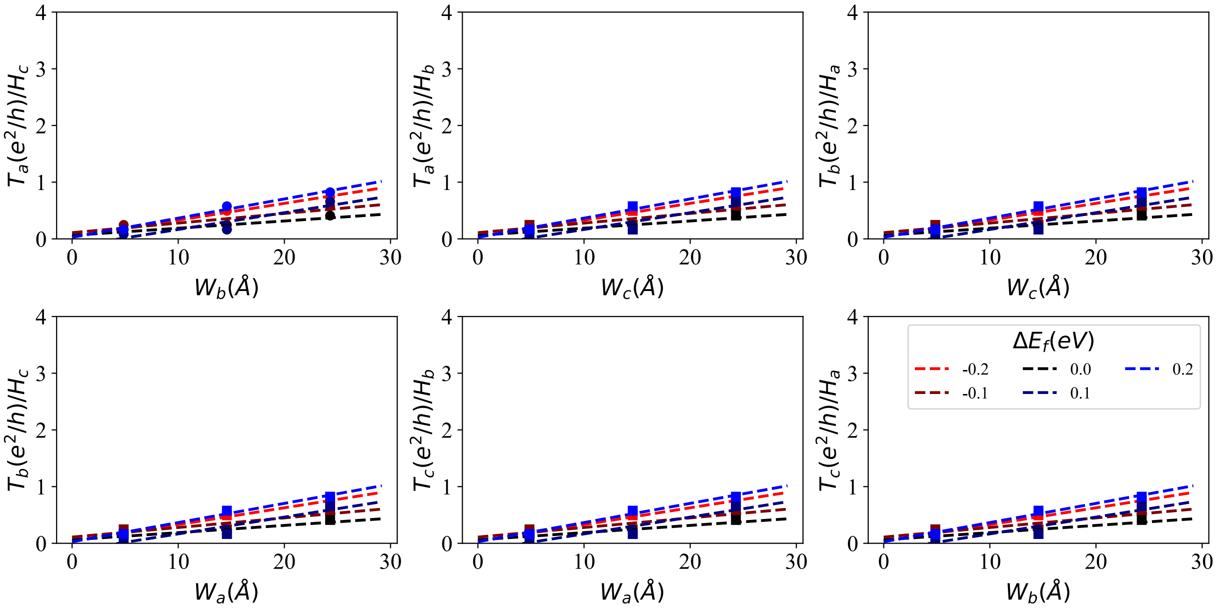

Compound: HfB_2
Materials Project ID: 1994

Lattice (conventional cell):

Parameter	Value	Unit
a	3.1487	Å
b	3.1487	Å
c	3.4799	Å
α (alpha)	90.0000	°
β (beta)	90.0000	°
γ (gamma)	120.0000	°

Crystal structure (conventional cell):

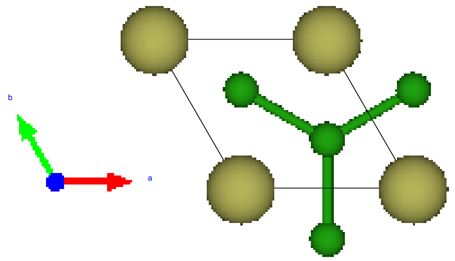

Nanowire Transmission:

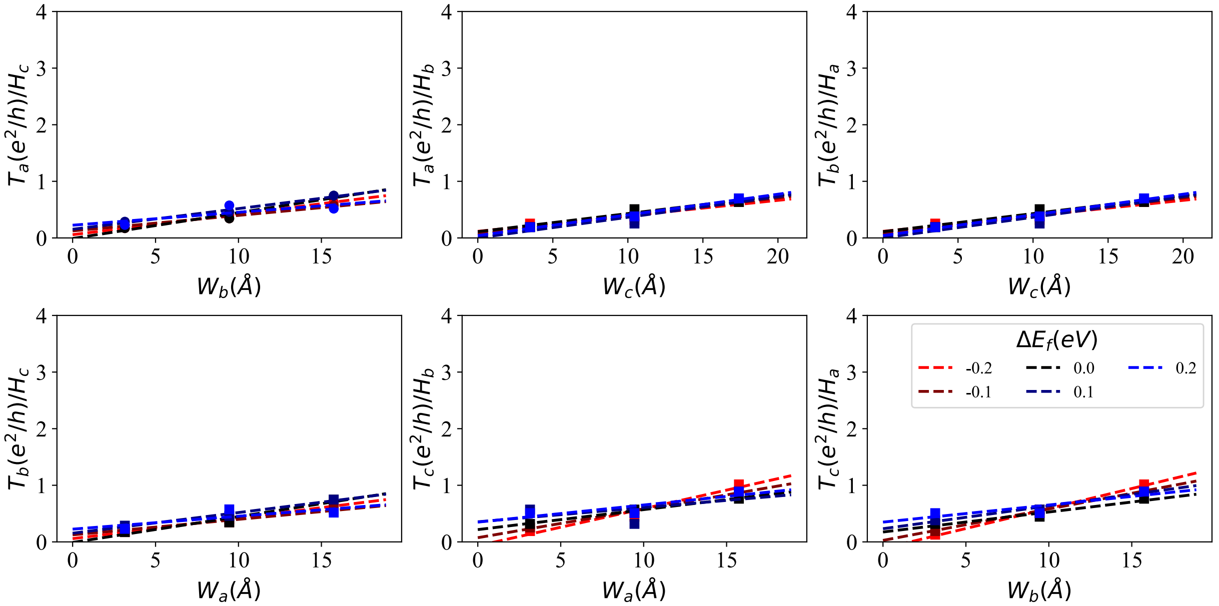

Compound: HfCuP
Materials Project ID: 569933

Lattice (conventional cell):

Parameter	Value	Unit
a	3.5872	Å
b	3.5872	Å
c	5.5801	Å
α (alpha)	90.0000	°
β (beta)	90.0000	°
γ (gamma)	120.0000	°

Crystal structure (conventional cell):

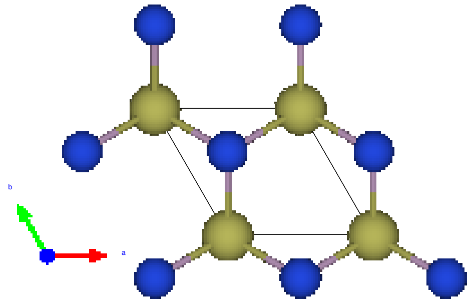

Nanowire Transmission:

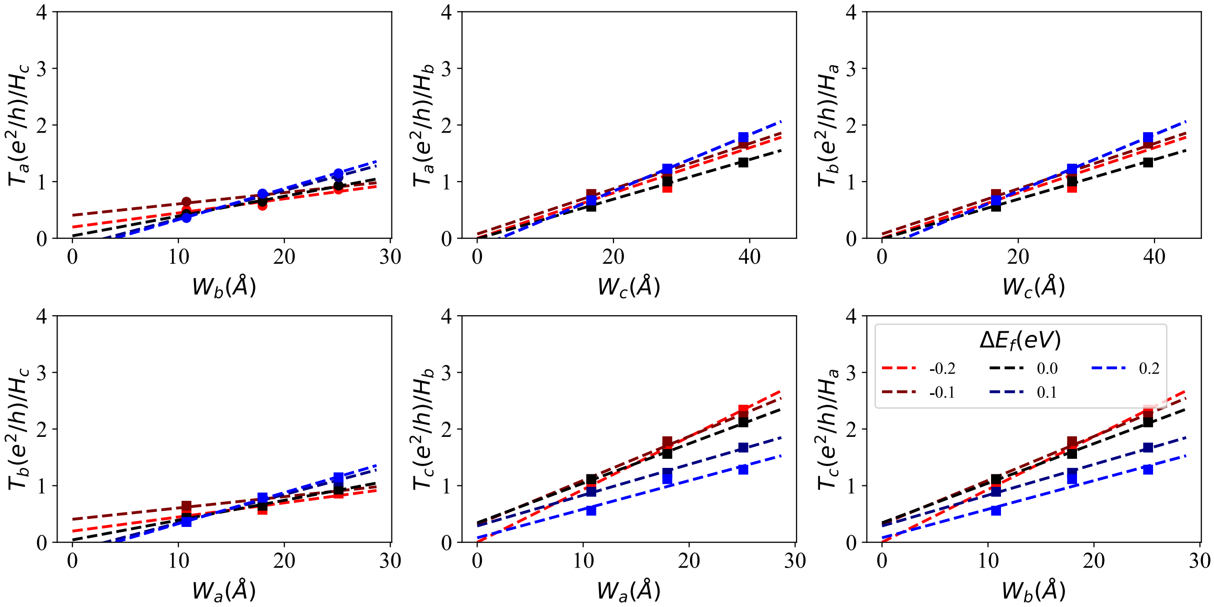

Compound: $InGaSe_2$
Materials Project ID: 1095059

Lattice (conventional cell):

Parameter	Value	Unit
a	8.1411	Å
b	8.1411	Å
c	6.4253	Å
α (alpha)	90.0000	°
β (beta)	90.0000	°
γ (gamma)	90.0000	°

Crystal structure (conventional cell):

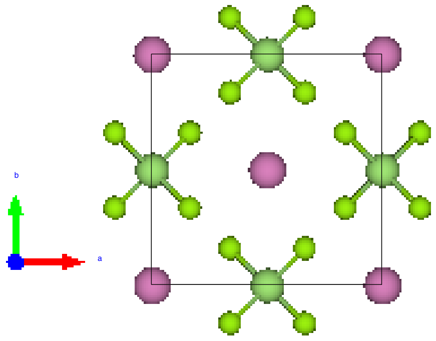

Nanowire Transmission:

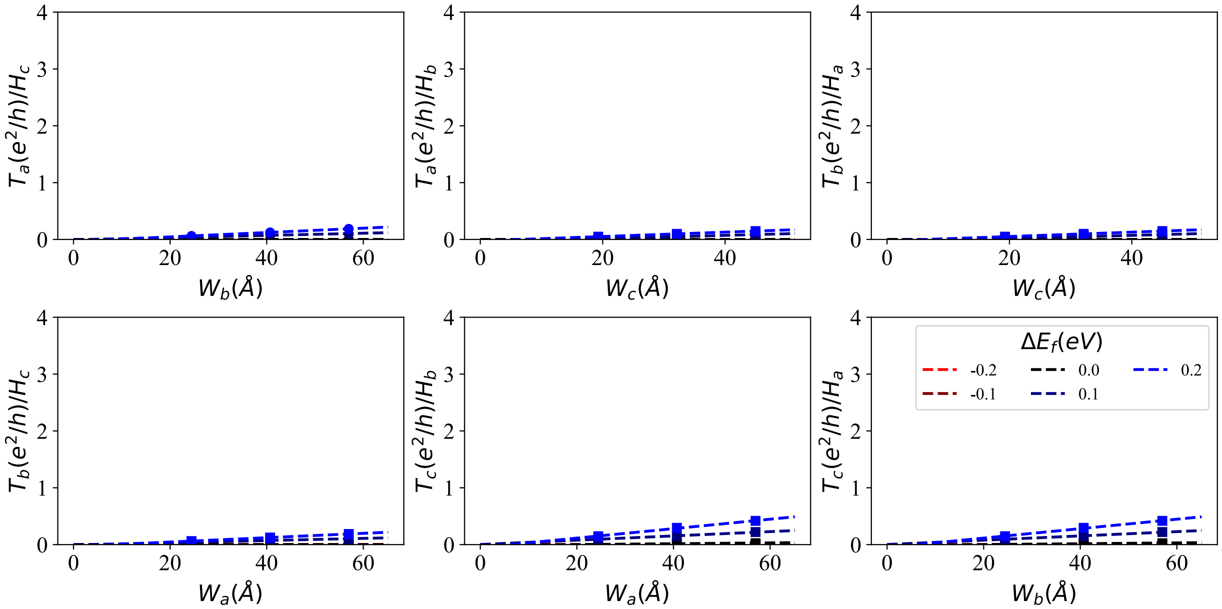

Compound: InTe
Materials Project ID: 20320

Lattice (conventional cell):

Parameter	Value	Unit
a	8.6274	Å
b	8.6274	Å
c	7.2267	Å
α (alpha)	90.0000	°
β (beta)	90.0000	°
γ (gamma)	90.0000	°

Crystal structure (conventional cell):

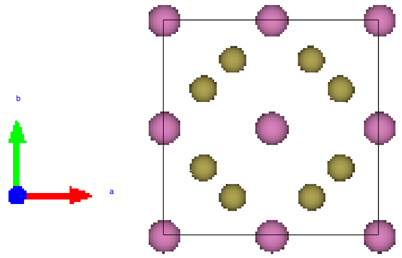

Nanowire Transmission:

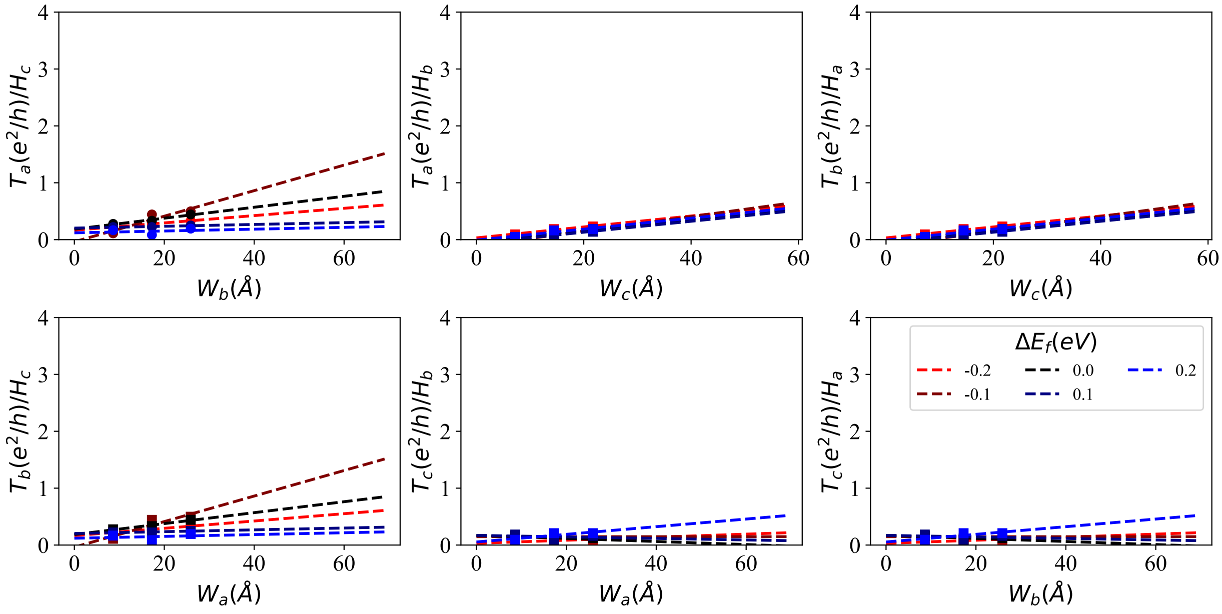

Compound: Ir
Materials Project ID: 101

Lattice (conventional cell):

Parameter	Value	Unit
a	3.8757	Å
b	3.8757	Å
c	3.8757	Å
α (alpha)	90.0000	°
β (beta)	90.0000	°
γ (gamma)	90.0000	°

Crystal structure (conventional cell):

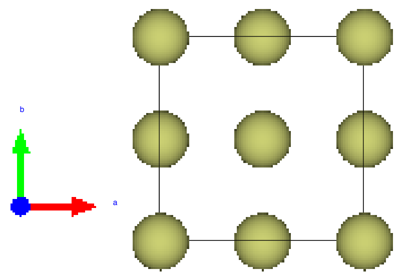

Nanowire Transmission:

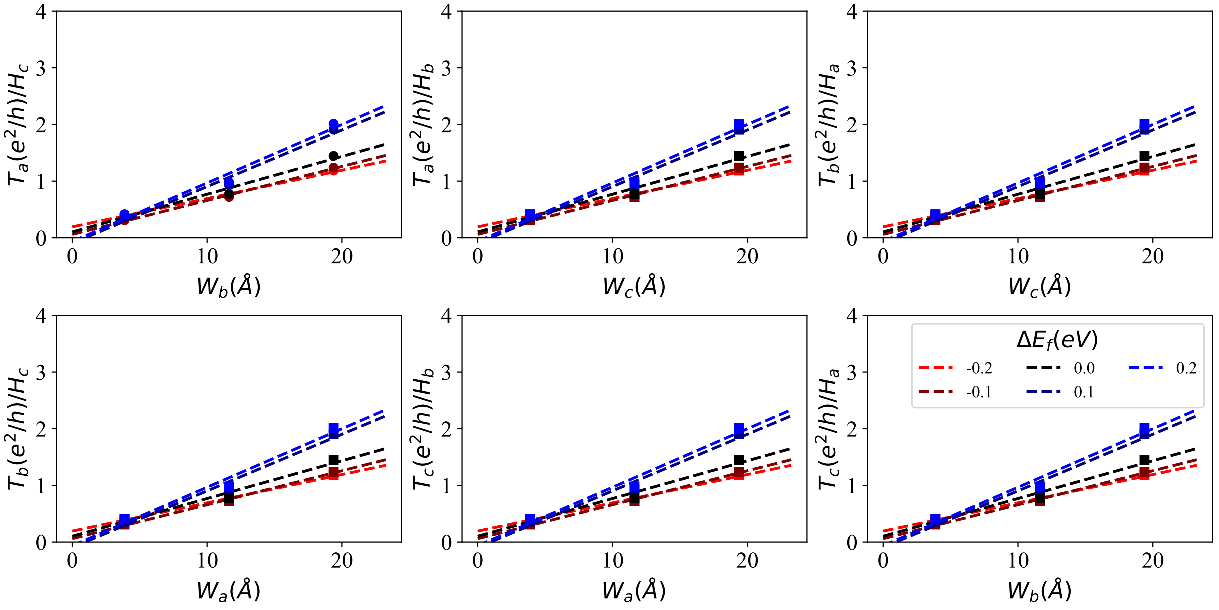

Compound: LaBiPt
Materials Project ID: 1018136

Lattice (conventional cell):

Parameter	Value	Unit
a	6.9689	Å
b	6.9689	Å
c	6.9689	Å
α (alpha)	90.0000	°
β (beta)	90.0000	°
γ (gamma)	90.0000	°

Crystal structure (conventional cell):

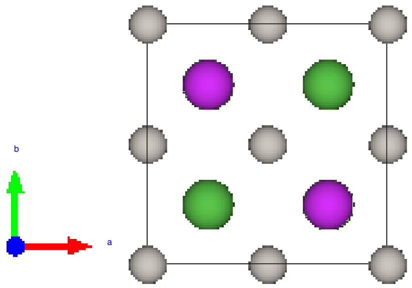

Nanowire Transmission:

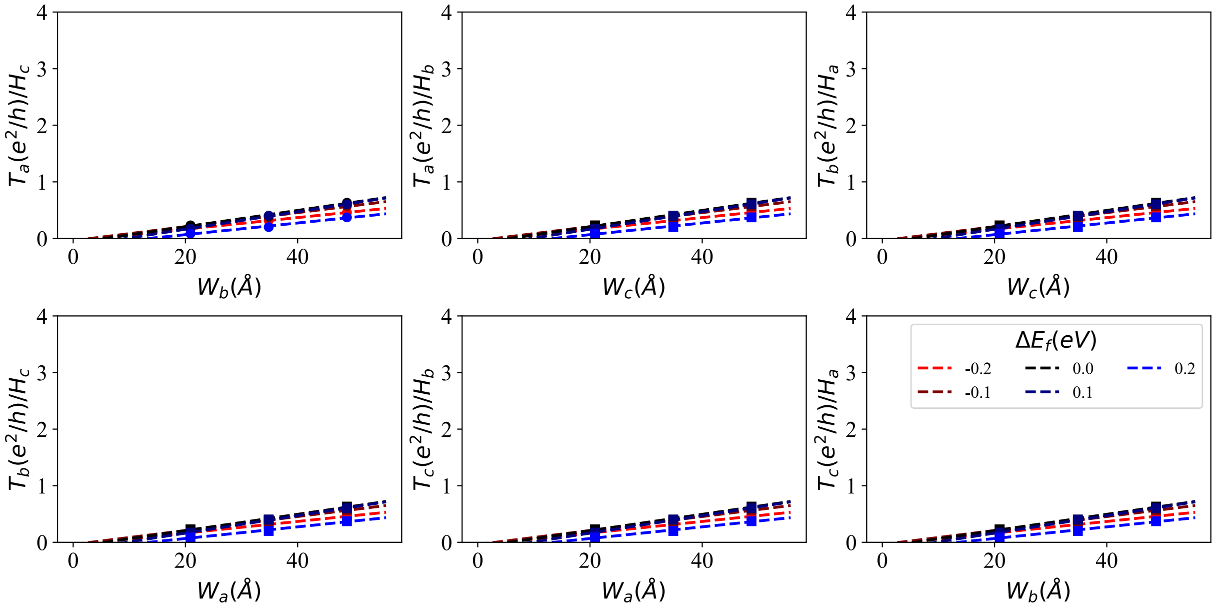

Compound: LaPIr
Materials Project ID: 12919

Lattice (conventional cell):

Parameter	Value	Unit
a	4.1709	Å
b	4.1709	Å
c	14.3855	Å
α (alpha)	90.0000	°
β (beta)	90.0000	°
γ (gamma)	90.0000	°

Crystal structure (conventional cell):

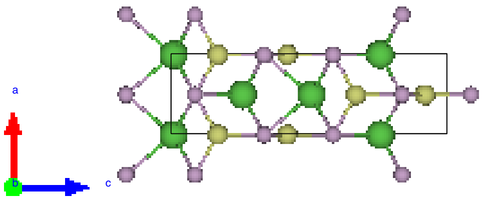

Nanowire Transmission:

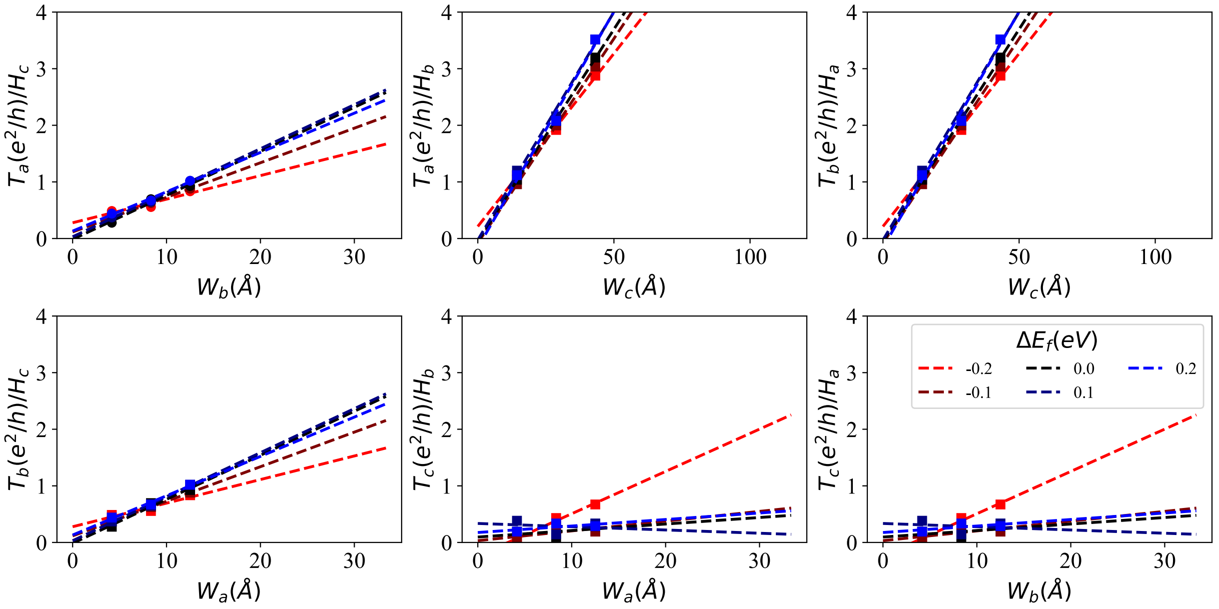

Compound: $LiClO_2$
Materials Project ID: 31367

Lattice (conventional cell):

Parameter	Value	Unit
a	4.8313	Å
b	4.8313	Å
c	10.6884	Å
α (alpha)	90.0000	°
β (beta)	90.0000	°
γ (gamma)	90.0000	°

Crystal structure (conventional cell):

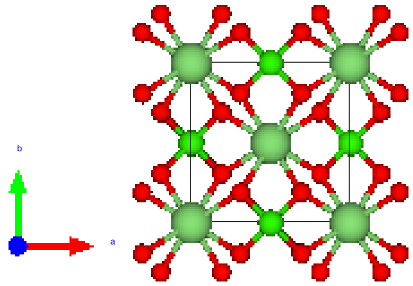

Nanowire Transmission:

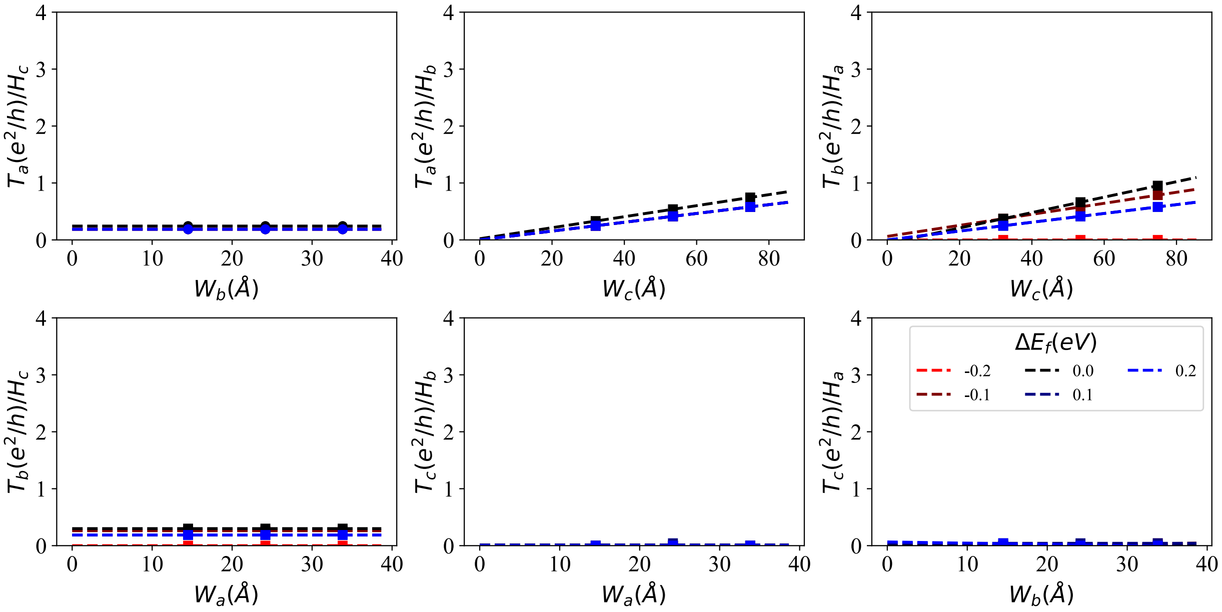

Compound: LiHO
Materials Project ID: 23856

Lattice (conventional cell):

Parameter	Value	Unit
a	3.5914	Å
b	3.5914	Å
c	4.4105	Å
α (alpha)	90.0000	°
β (beta)	90.0000	°
γ (gamma)	90.0000	°

Crystal structure (conventional cell):

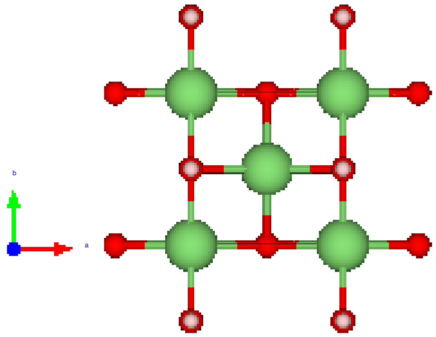

Nanowire Transmission:

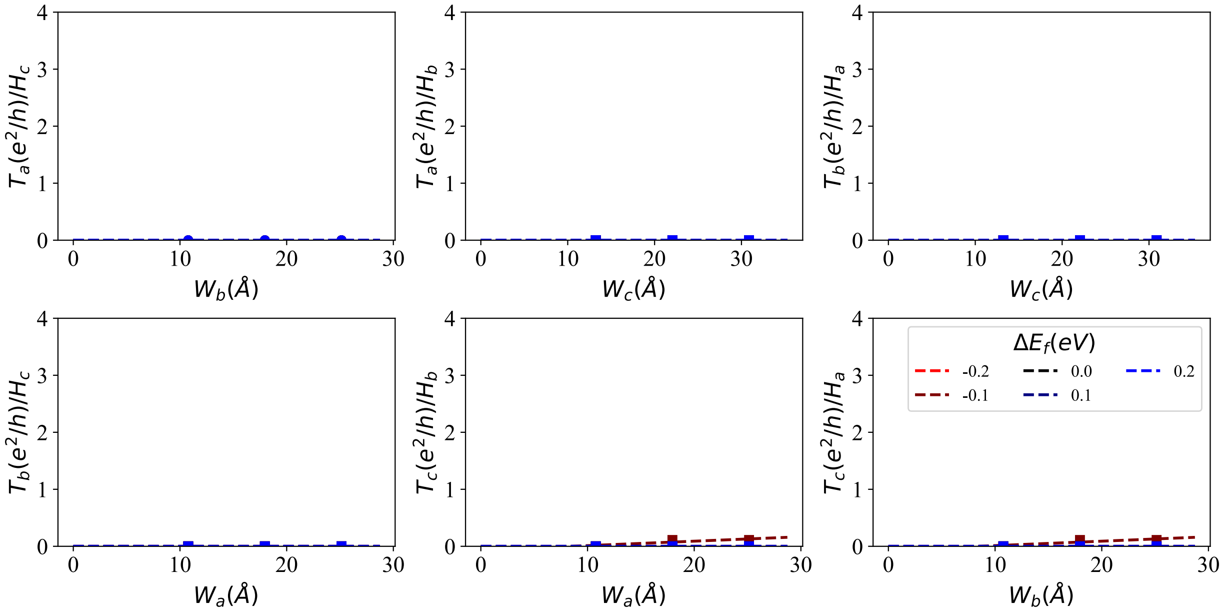

Compound: LiYGe
Materials Project ID: 14209

Lattice (conventional cell):

Parameter	Value	Unit
a	7.0924	Å
b	7.0924	Å
c	4.2591	Å
α (alpha)	90.0000	°
β (beta)	90.0000	°
γ (gamma)	120.0000	°

Crystal structure (conventional cell):

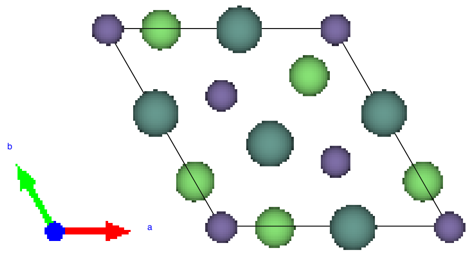

Nanowire Transmission:

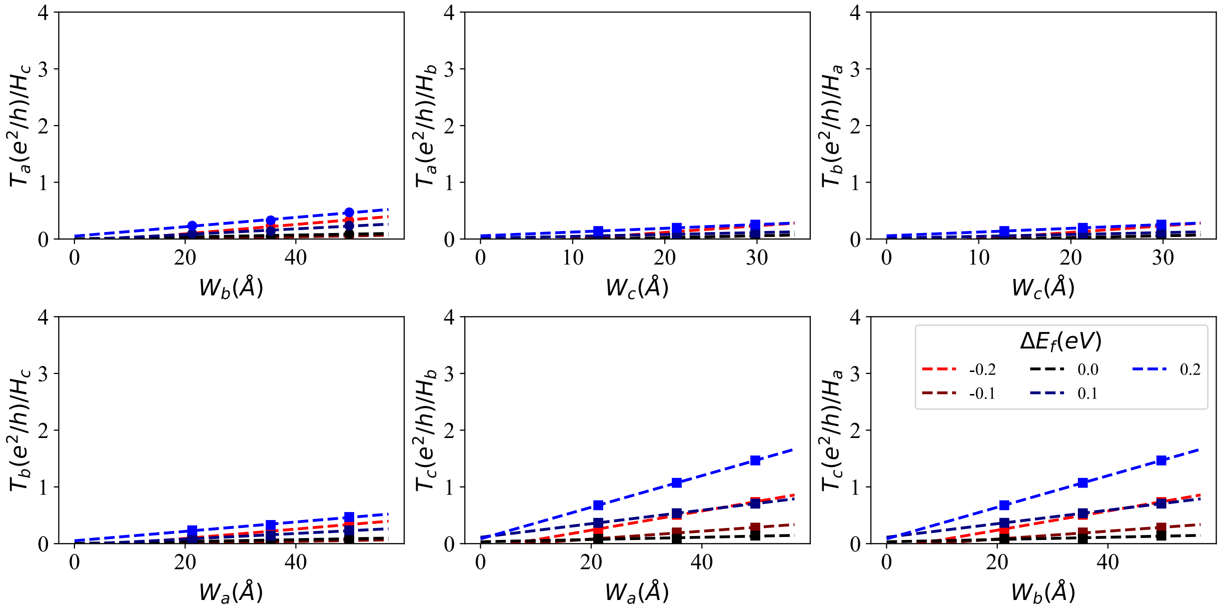

Compound: LiYSi
Materials Project ID: 14208

Lattice (conventional cell):

Parameter	Value	Unit
a	7.0152	Å
b	7.0152	Å
c	4.2400	Å
α (alpha)	90.0000	°
β (beta)	90.0000	°
γ (gamma)	120.0000	°

Crystal structure (conventional cell):

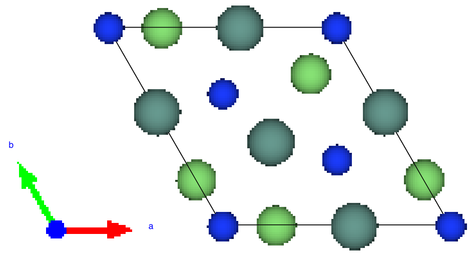

Nanowire Transmission:

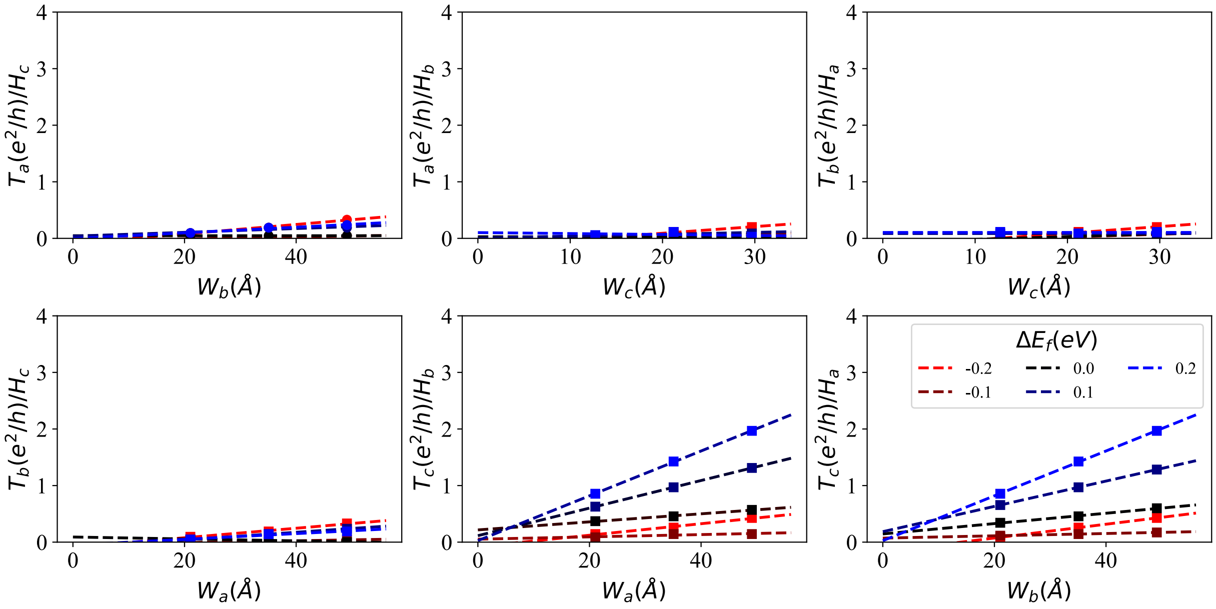

Compound: Mo
Materials Project ID: 129

Lattice (conventional cell):

Parameter	Value	Unit
a	3.1676	Å
b	3.1676	Å
c	3.1676	Å
α (alpha)	90.0000	°
β (beta)	90.0000	°
γ (gamma)	90.0000	°

Crystal structure (conventional cell):

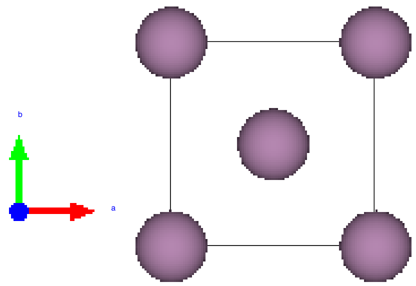

Nanowire Transmission:

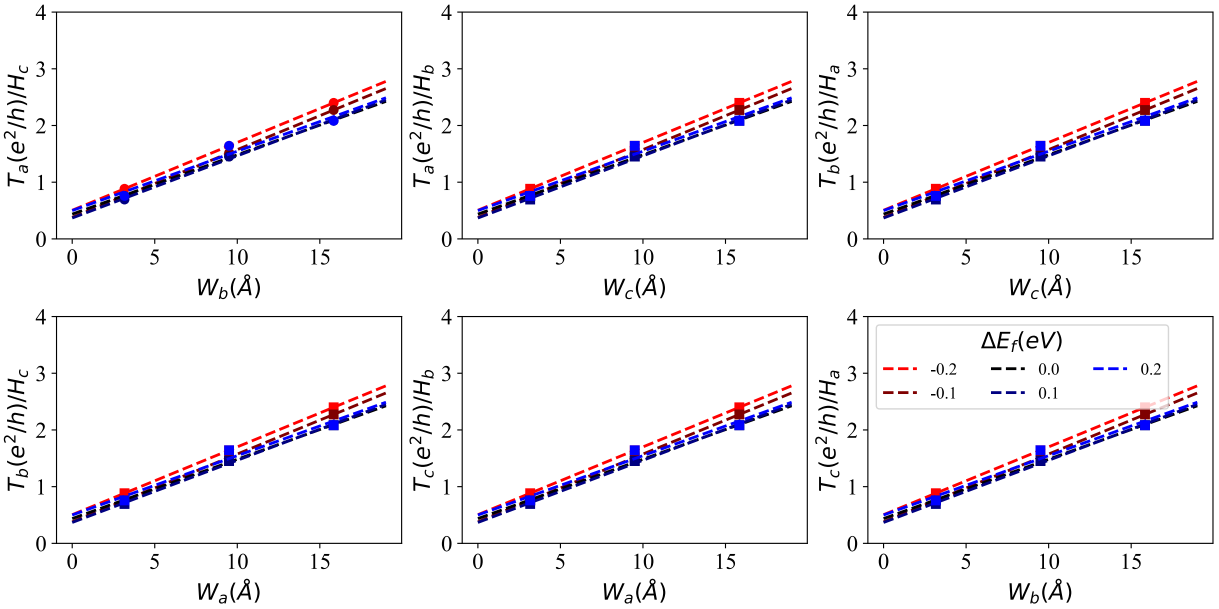

Compound: MoC
Materials Project ID: 2305

Lattice (conventional cell):

Parameter	Value	Unit
a	2.9241	Å
b	2.9241	Å
c	2.8351	Å
α (alpha)	90.0000	°
β (beta)	90.0000	°
γ (gamma)	120.0000	°

Crystal structure (conventional cell):

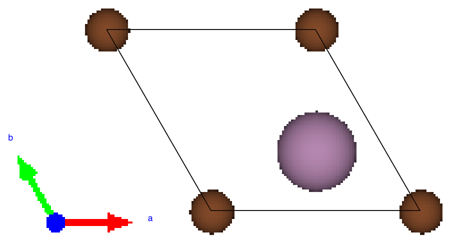

Nanowire Transmission:

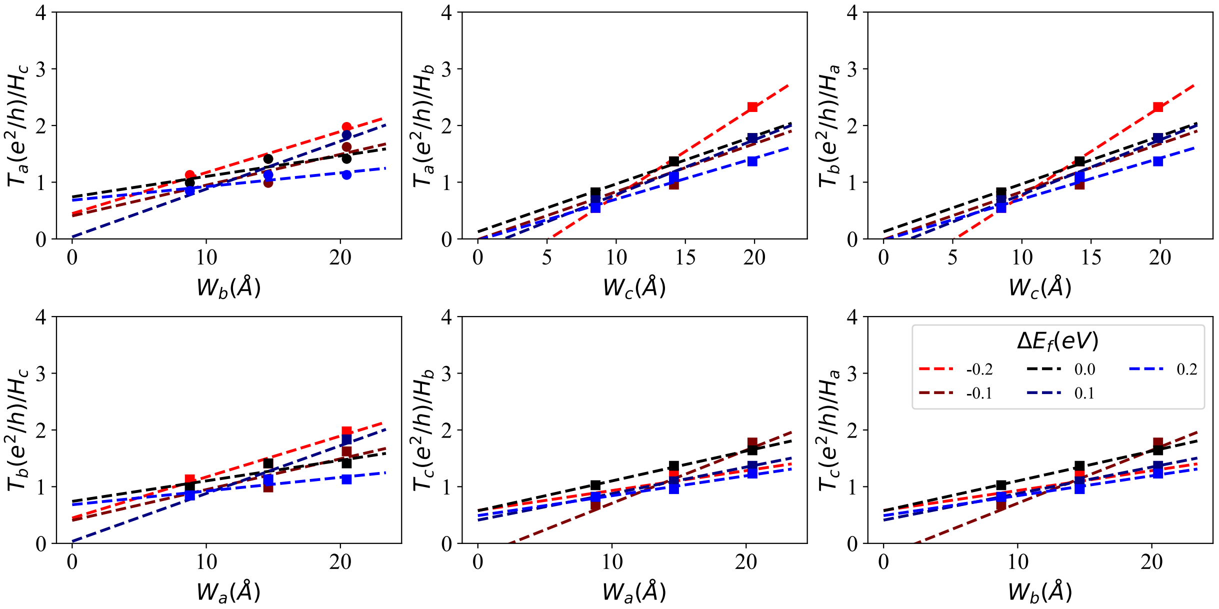

Compound: MoN
Materials Project ID: 13036

Lattice (conventional cell):

Parameter	Value	Unit
a	2.8858	Å
b	2.8858	Å
c	2.8561	Å
α (alpha)	90.0000	°
β (beta)	90.0000	°
γ (gamma)	120.0000	°

Crystal structure (conventional cell):

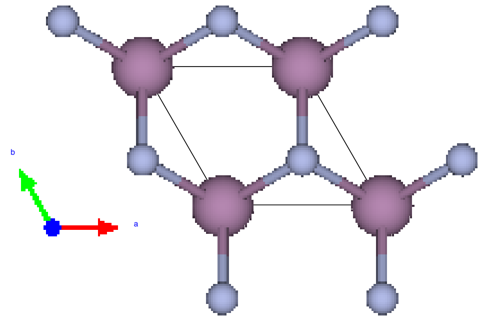

Nanowire Transmission:

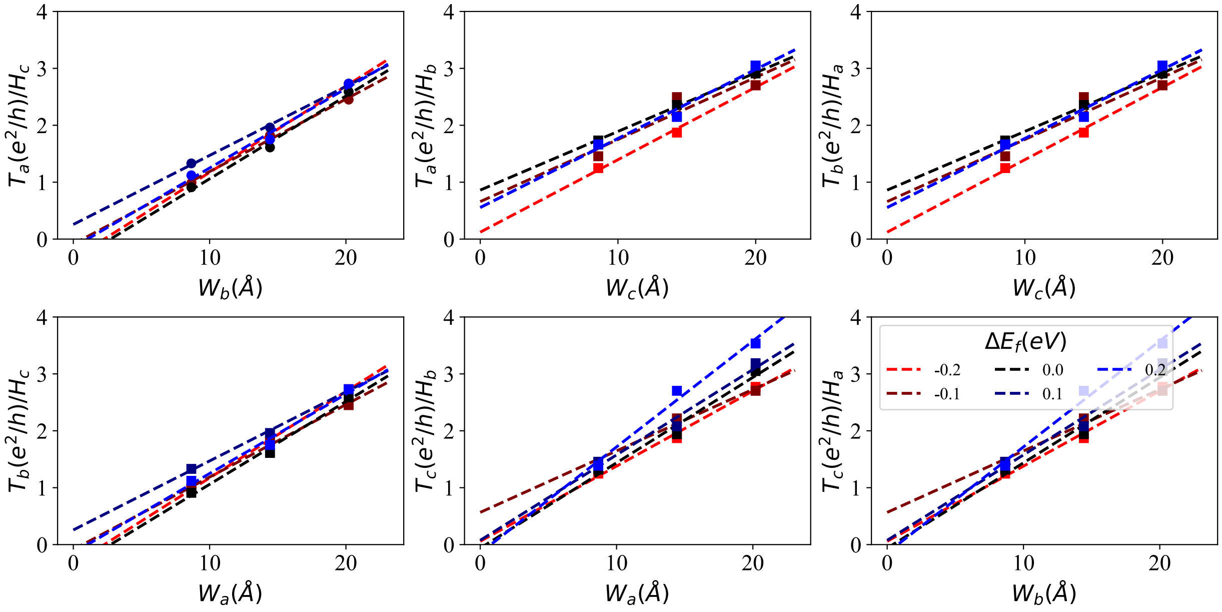

Compound: MoP
Materials Project ID: 219

Lattice (conventional cell):

Parameter	Value	Unit
a	3.2446	Å
b	3.2446	Å
c	3.2005	Å
α (alpha)	90.0000	°
β (beta)	90.0000	°
γ (gamma)	120.0000	°

Crystal structure (conventional cell):

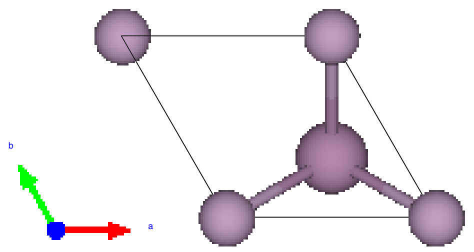

Nanowire Transmission:

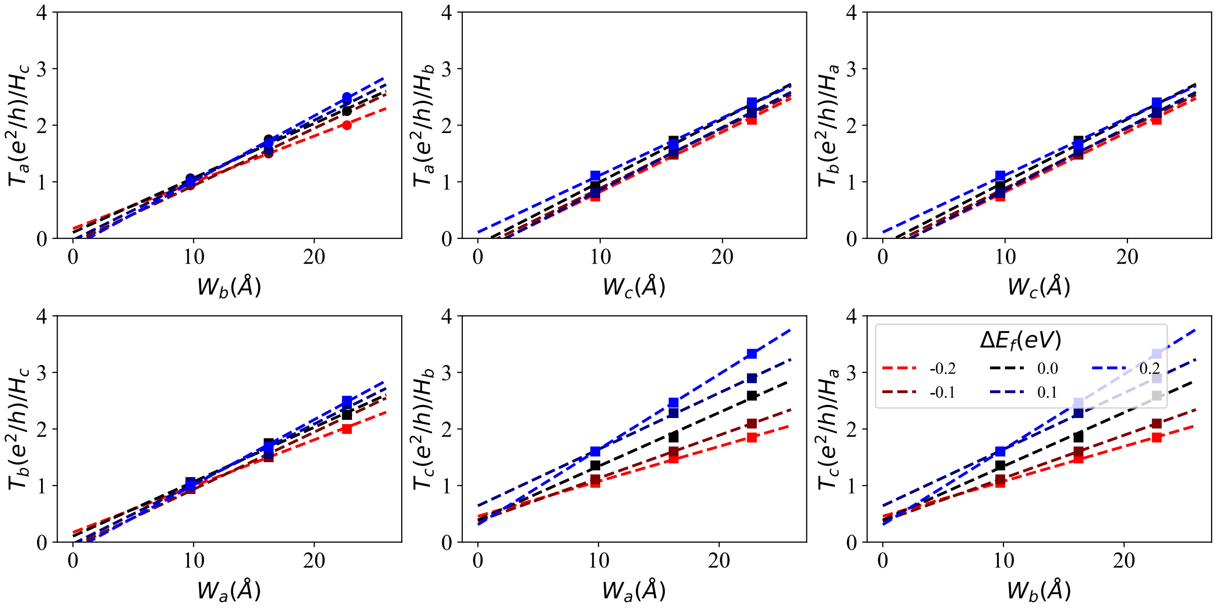

Compound: NbAs
Materials Project ID: 2059

Lattice (conventional cell):

Parameter	Value	Unit
a	3.4847	Å
b	3.4847	Å
c	11.7615	Å
α (alpha)	90.0000	°
β (beta)	90.0000	°
γ (gamma)	90.0000	°

Crystal structure (conventional cell):

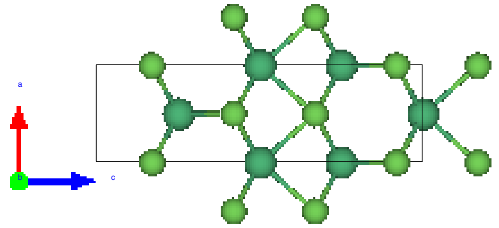

Nanowire Transmission:

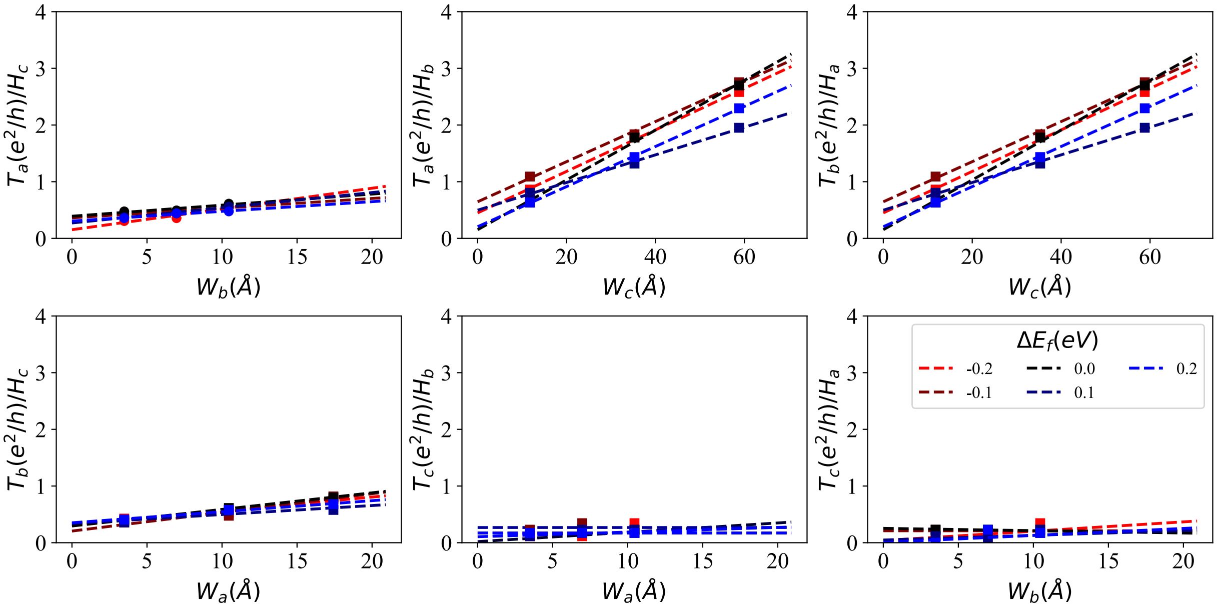

Compound: $NbAs_2$
Materials Project ID: 7598

Lattice (conventional cell):

Parameter	Value	Unit
a	9.4518	Å
b	3.4178	Å
c	7.8850	Å
α (alpha)	90.0000	°
β (beta)	119.3863	°
γ (gamma)	90.0000	°

Crystal structure (conventional cell):

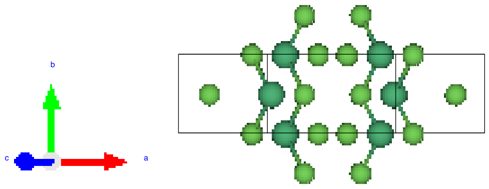

Nanowire Transmission:

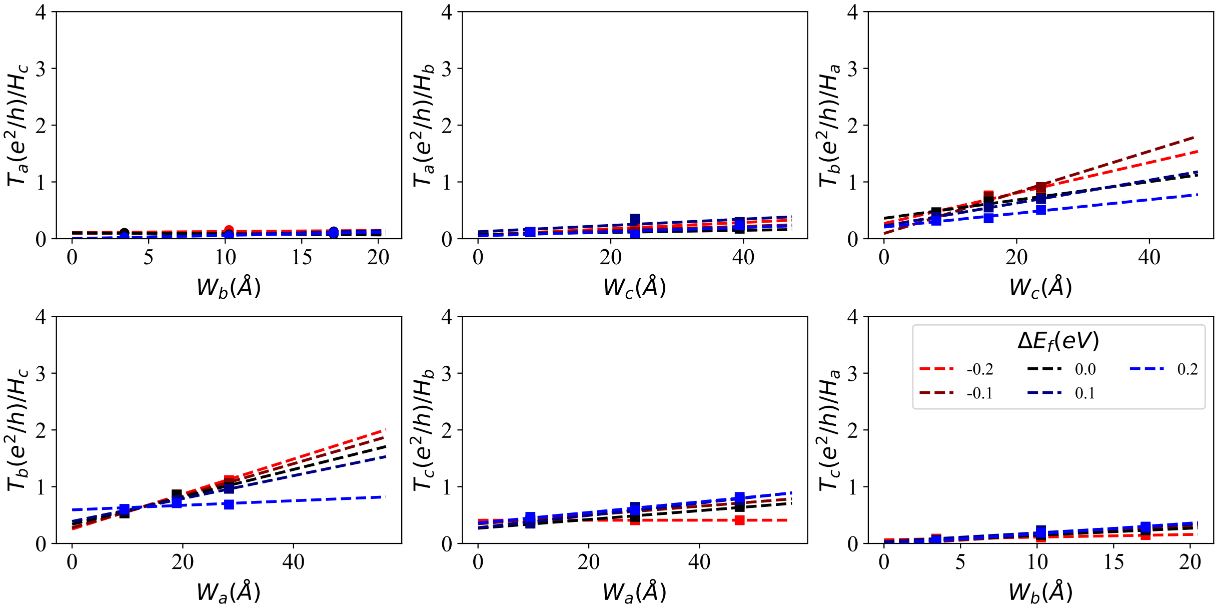

Compound: $NbInSe_2$
Materials Project ID: 20279

Lattice (conventional cell):

Parameter	Value	Unit
a	3.4646	Å
b	3.4646	Å
c	9.3914	Å
α (alpha)	90.0000	°
β (beta)	90.0000	°
γ (gamma)	120.0000	°

Crystal structure (conventional cell):

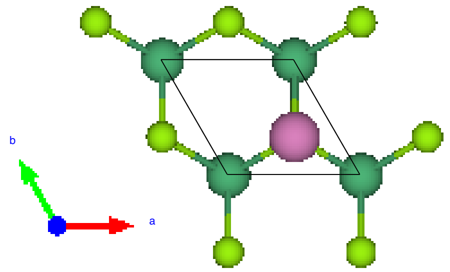

Nanowire Transmission:

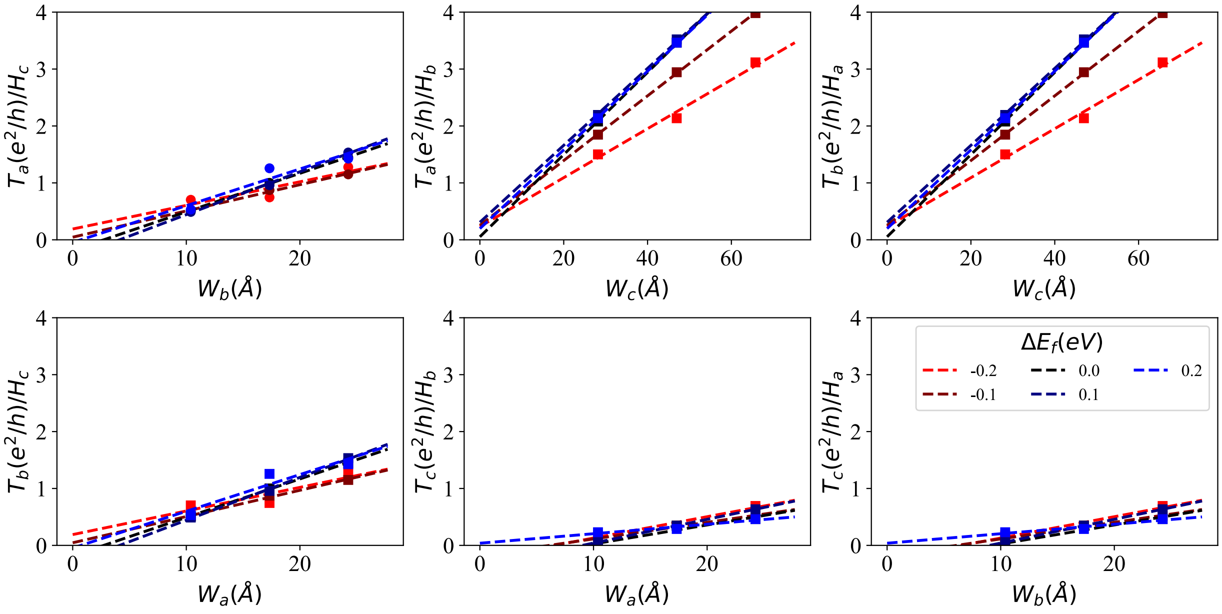

Compound: NbN
Materials Project ID: 2634

Lattice (conventional cell):

Parameter	Value	Unit
a	2.9762	Å
b	2.9762	Å
c	2.8987	Å
α (alpha)	90.0000	°
β (beta)	90.0000	°
γ (gamma)	120.0000	°

Crystal structure (conventional cell):

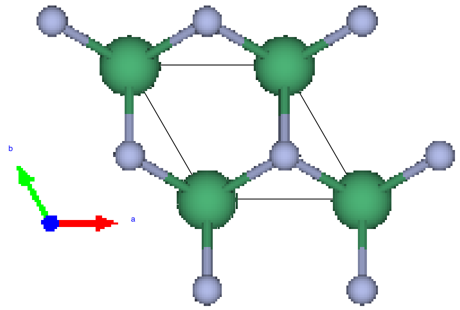

Nanowire Transmission:

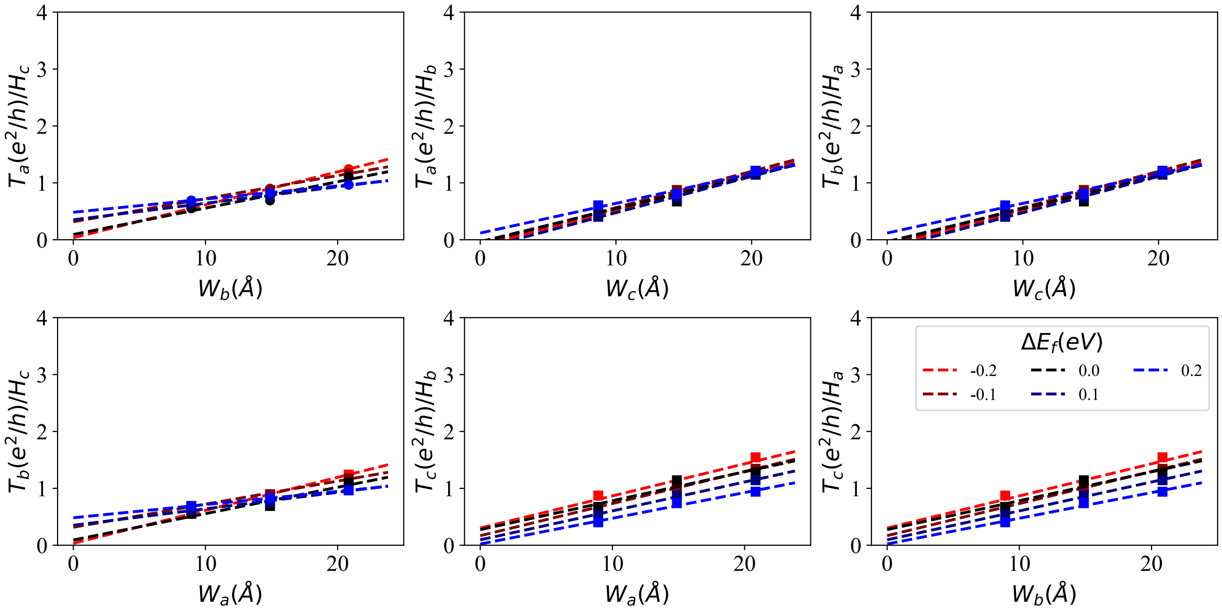

Compound: NbP
Materials Project ID: 9339

Lattice (conventional cell):

Parameter	Value	Unit
a	3.3564	Å
b	3.3564	Å
c	11.4365	Å
α (alpha)	90.0000	°
β (beta)	90.0000	°
γ (gamma)	90.0000	°

Crystal structure (conventional cell):

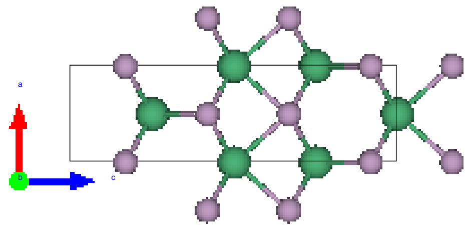

Nanowire Transmission:

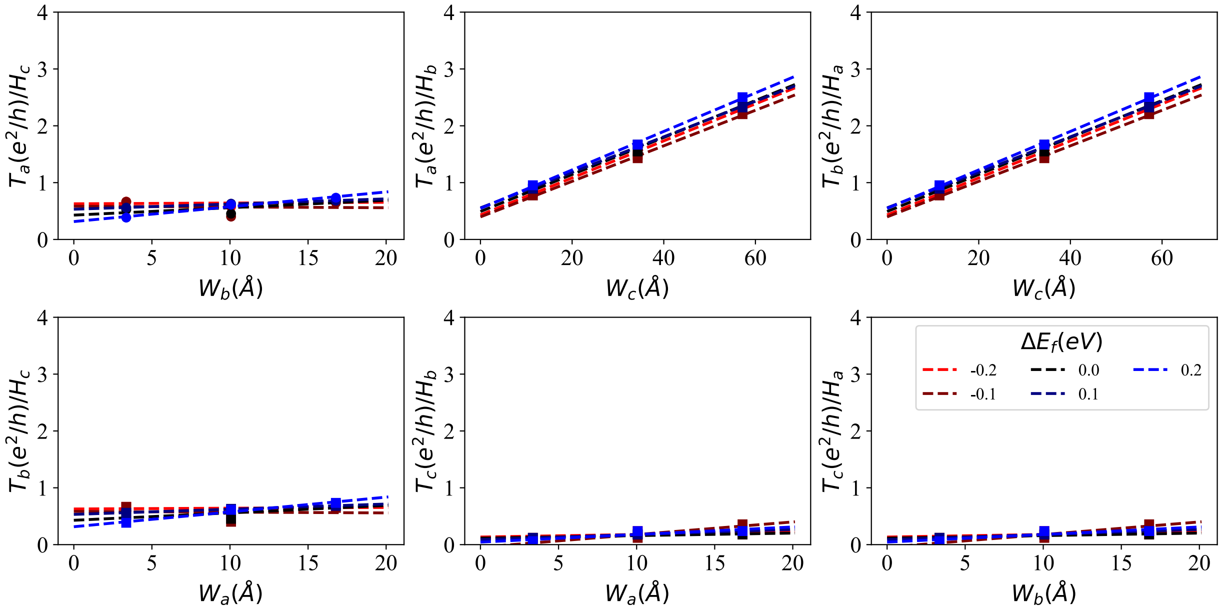

Compound: NbP_2
Materials Project ID: 1077116

Lattice (conventional cell):

Parameter	Value	Unit
a	8.9129	Å
b	3.2925	Å
c	7.5664	Å
α (alpha)	90.0000	°
β (beta)	118.9943	°
γ (gamma)	90.0000	°

Crystal structure (conventional cell):

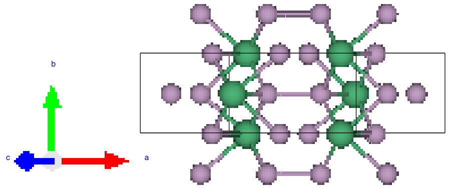

Nanowire Transmission:

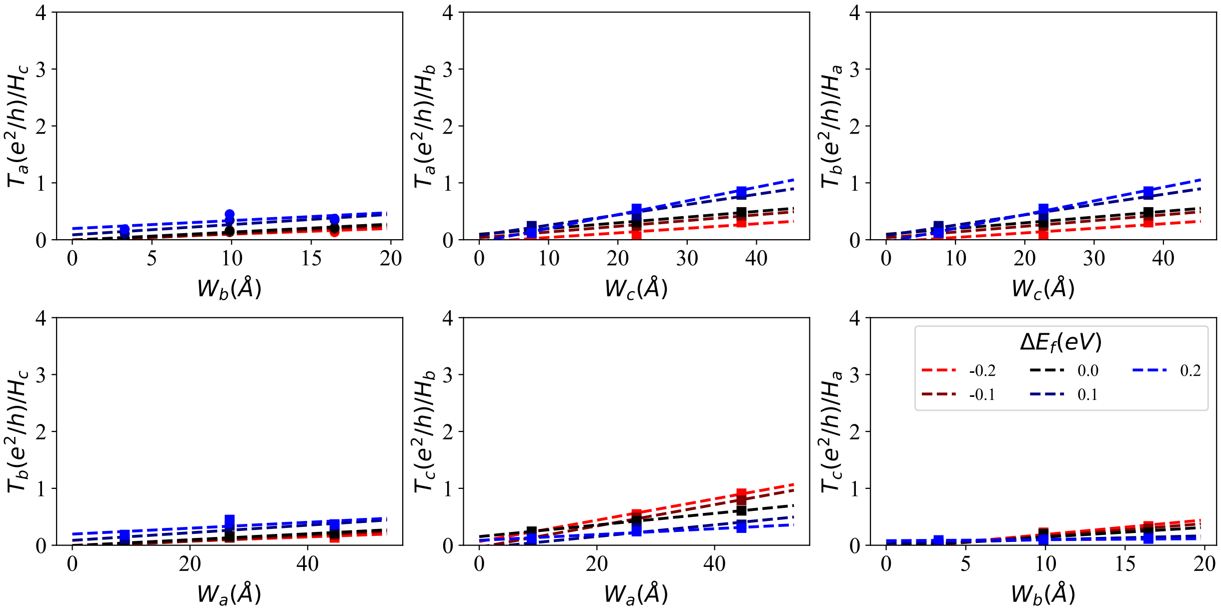

Compound: NbS
Materials Project ID: 2243

Lattice (conventional cell):

Parameter	Value	Unit
a	3.3186	Å
b	3.3186	Å
c	3.3243	Å
α (alpha)	90.0000	°
β (beta)	90.0000	°
γ (gamma)	120.0000	°

Crystal structure (conventional cell):

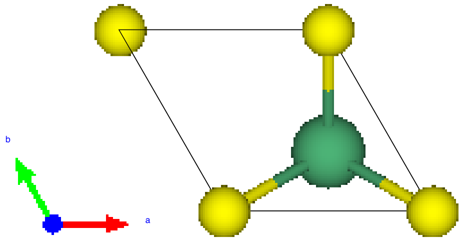

Nanowire Transmission:

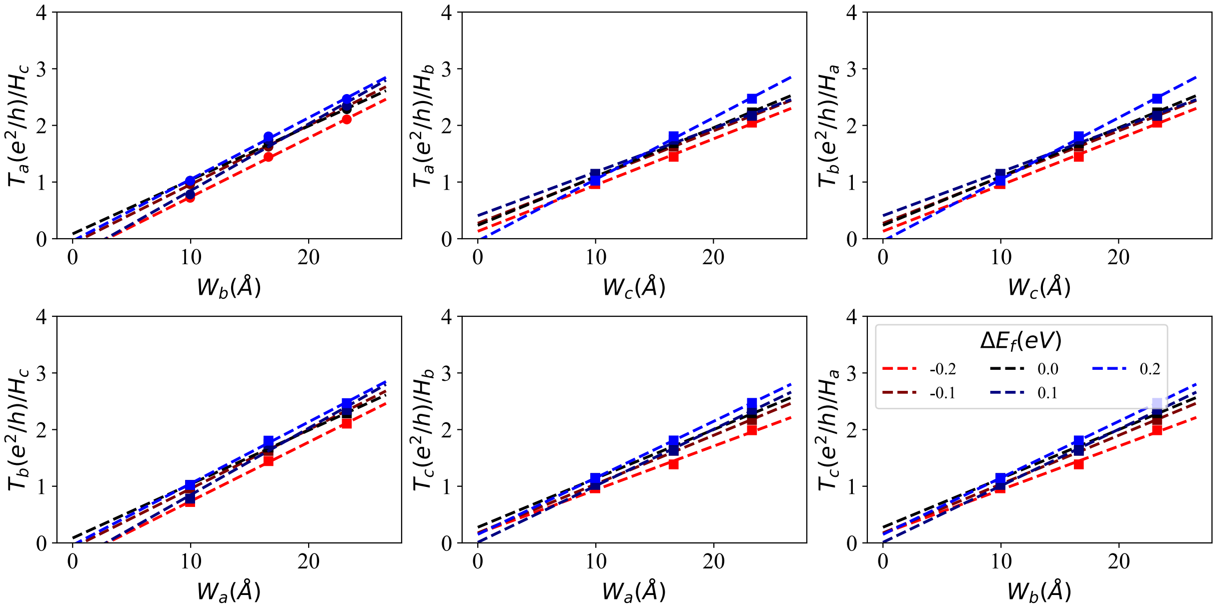

Compound: $NbSe_4$
Materials Project ID: 1078815

Lattice (conventional cell):

Parameter	Value	Unit
a	5.9708	Å
b	5.9708	Å
c	6.5262	Å
α (alpha)	90.0000	°
β (beta)	90.0000	°
γ (gamma)	90.0000	°

Crystal structure (conventional cell):

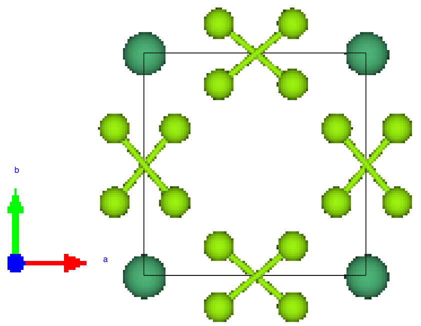

Nanowire Transmission:

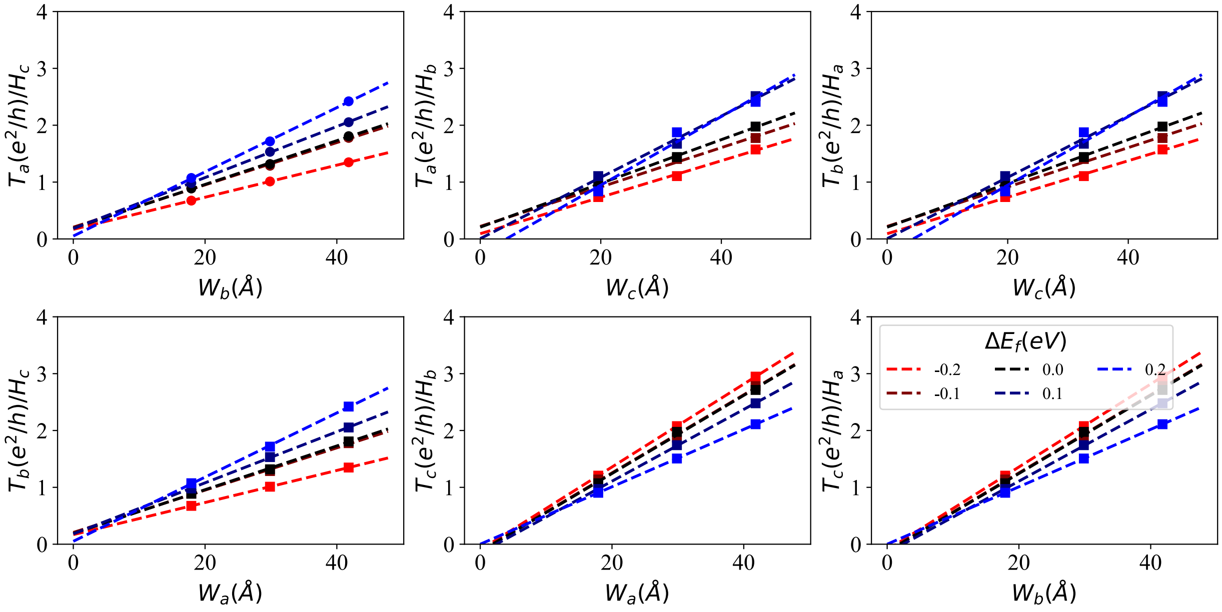

Compound: NbSiAs
Materials Project ID: 21907

Lattice (conventional cell):

Parameter	Value	Unit
a	3.5165	Å
b	3.5165	Å
c	7.9705	Å
α (alpha)	90.0000	°
β (beta)	90.0000	°
γ (gamma)	90.0000	°

Crystal structure (conventional cell):

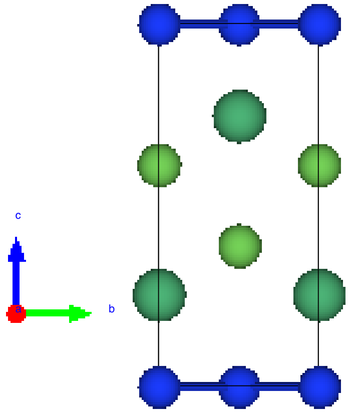

Nanowire Transmission:

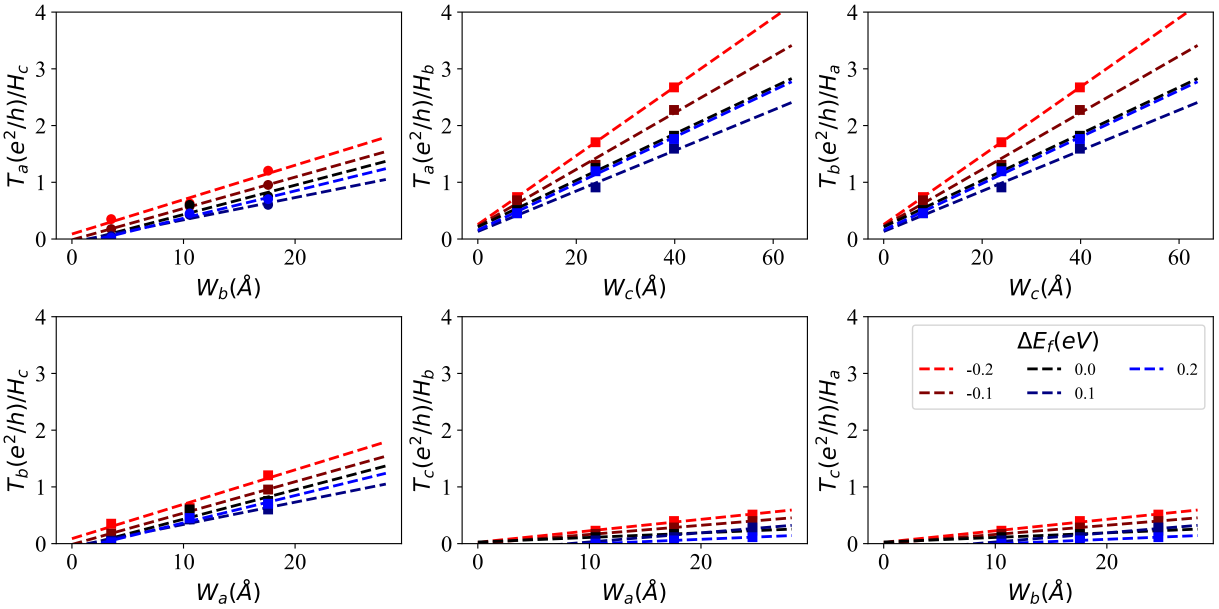

Compound: Ni(BMo)$_2$
Materials Project ID: 9999

Lattice (conventional cell):

Parameter	Value	Unit
a	3.1850	Å
b	4.5764	Å
c	7.1383	Å
α (alpha)	90.0000	°
β (beta)	90.0000	°
γ (gamma)	90.0000	°

Crystal structure (conventional cell):

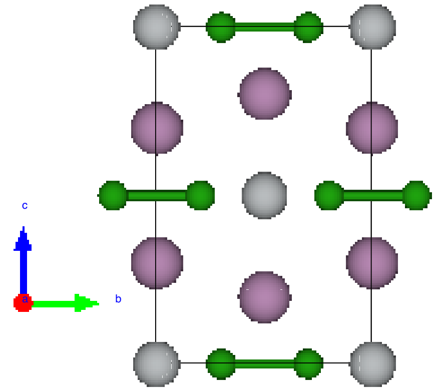

Nanowire Transmission:

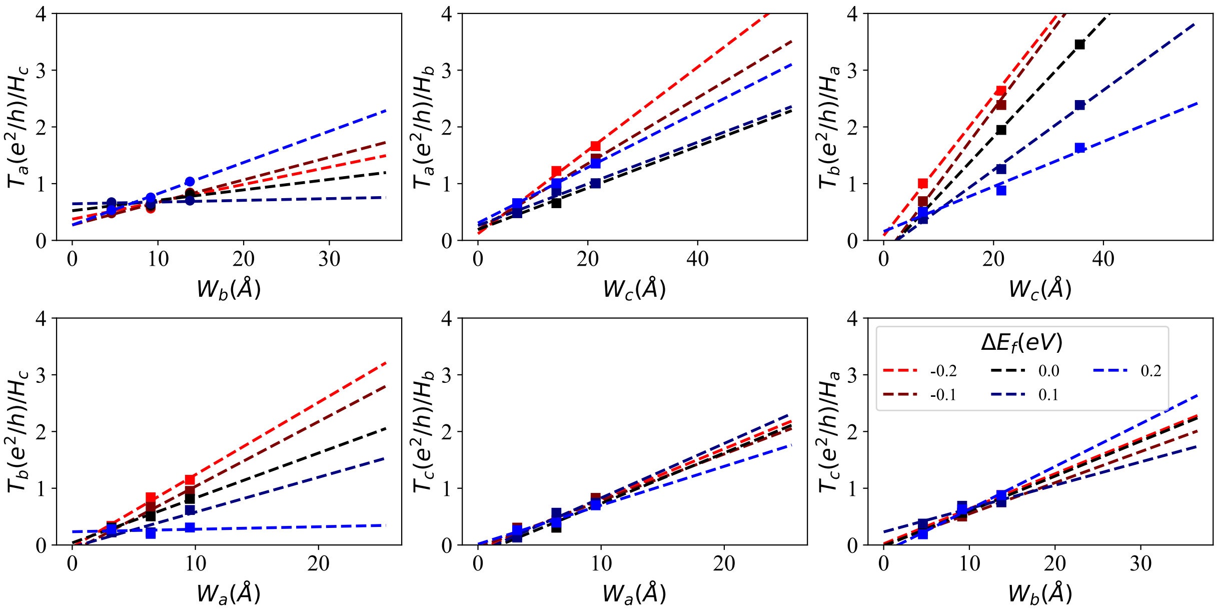

Compound: NiS
Materials Project ID: 1547

Lattice (conventional cell):

Parameter	Value	Unit
a	9.5644	Å
b	9.5644	Å
c	3.1260	Å
α (alpha)	90.0000	°
β (beta)	90.0000	°
γ (gamma)	120.0000	°

Crystal structure (conventional cell):

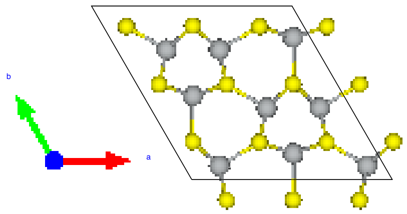

Nanowire Transmission:

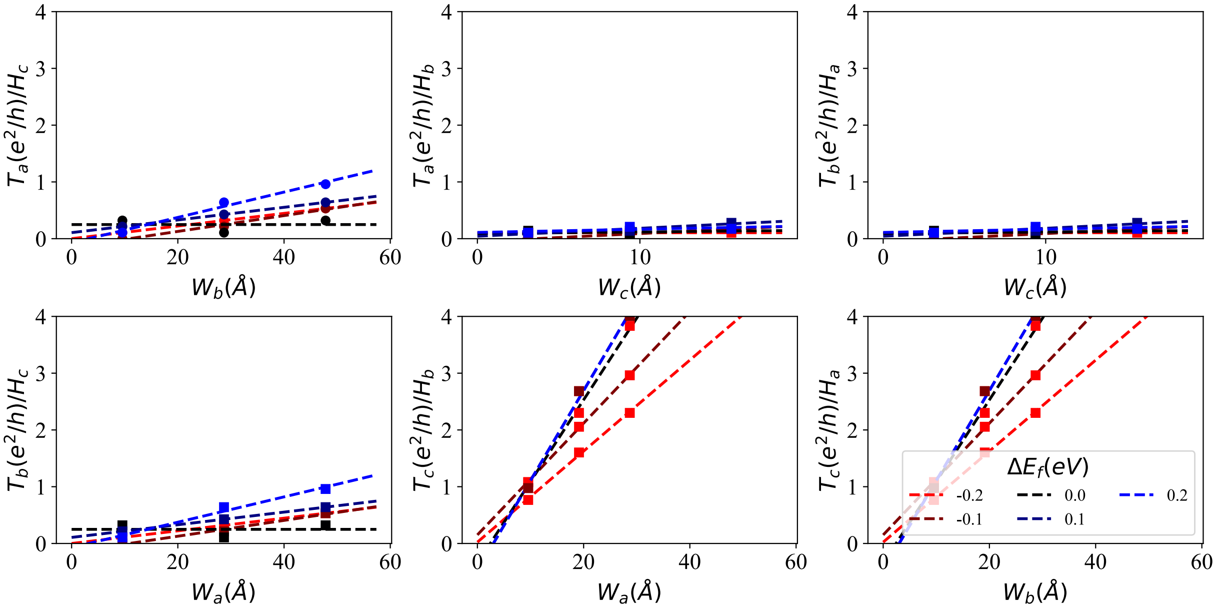

Compound: NiSe
Materials Project ID: 15651

Lattice (conventional cell):

Parameter	Value	Unit
a	10.0013	Å
b	10.0013	Å
c	3.3350	Å
α (alpha)	90.0000	°
β (beta)	90.0000	°
γ (gamma)	120.0000	°

Crystal structure (conventional cell):

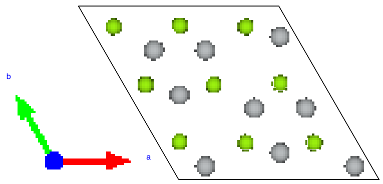

Nanowire Transmission:

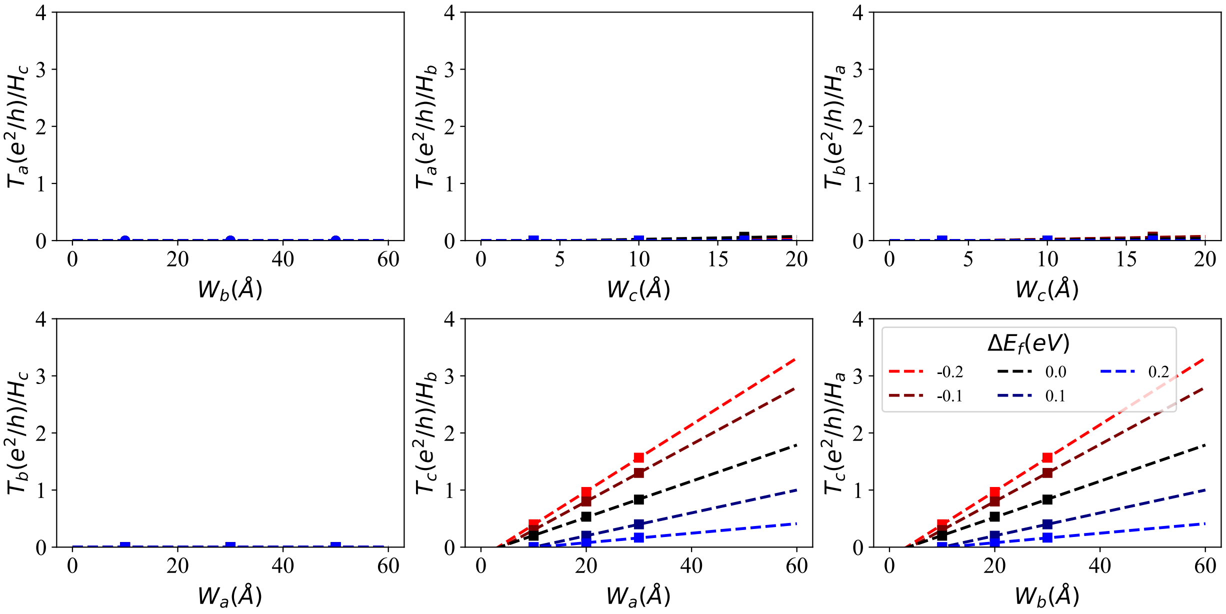

Compound: Pd_2N
Materials Project ID: 510087

Lattice (conventional cell):

Parameter	Value	Unit
a	2.9424	Å
b	4.8726	Å
c	5.4625	Å
α (alpha)	90.0000	°
β (beta)	90.0000	°
γ (gamma)	90.0000	°

Crystal structure (conventional cell):

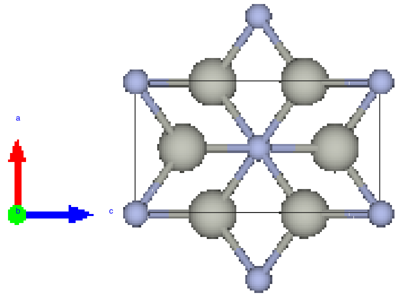

Nanowire Transmission:

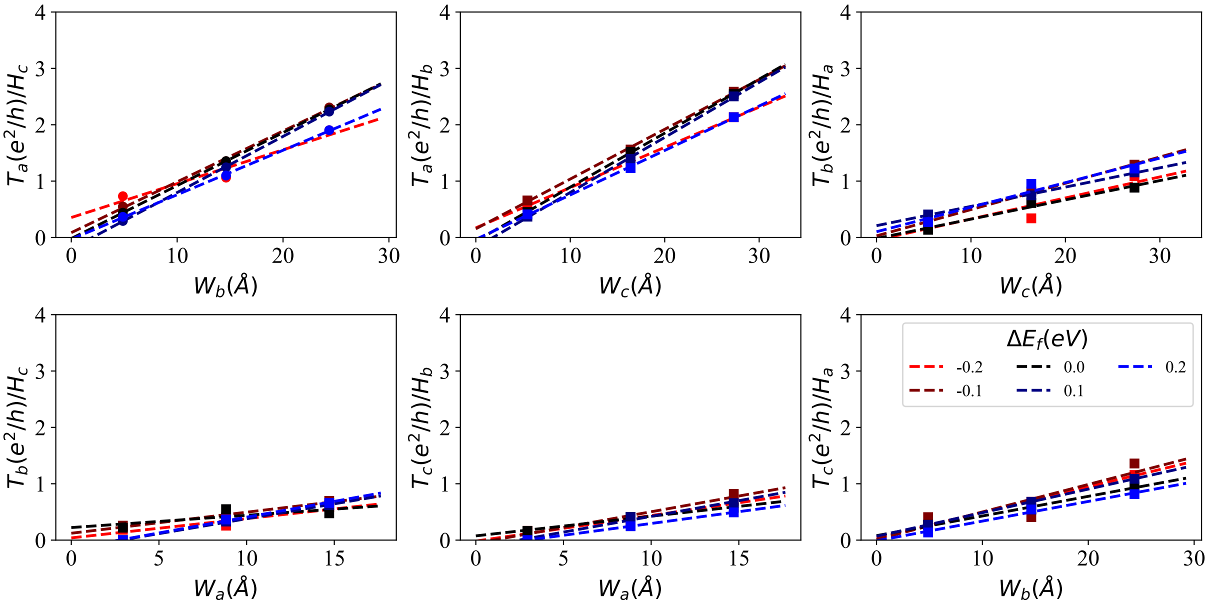

Compound: Re
Materials Project ID: 8

Lattice (conventional cell):

Parameter	Value	Unit
a	2.7811	Å
b	2.7811	Å
c	4.4971	Å
α (alpha)	90.0000	°
β (beta)	90.0000	°
γ (gamma)	120.0000	°

Crystal structure (conventional cell):

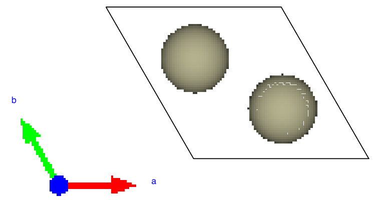

Nanowire Transmission:

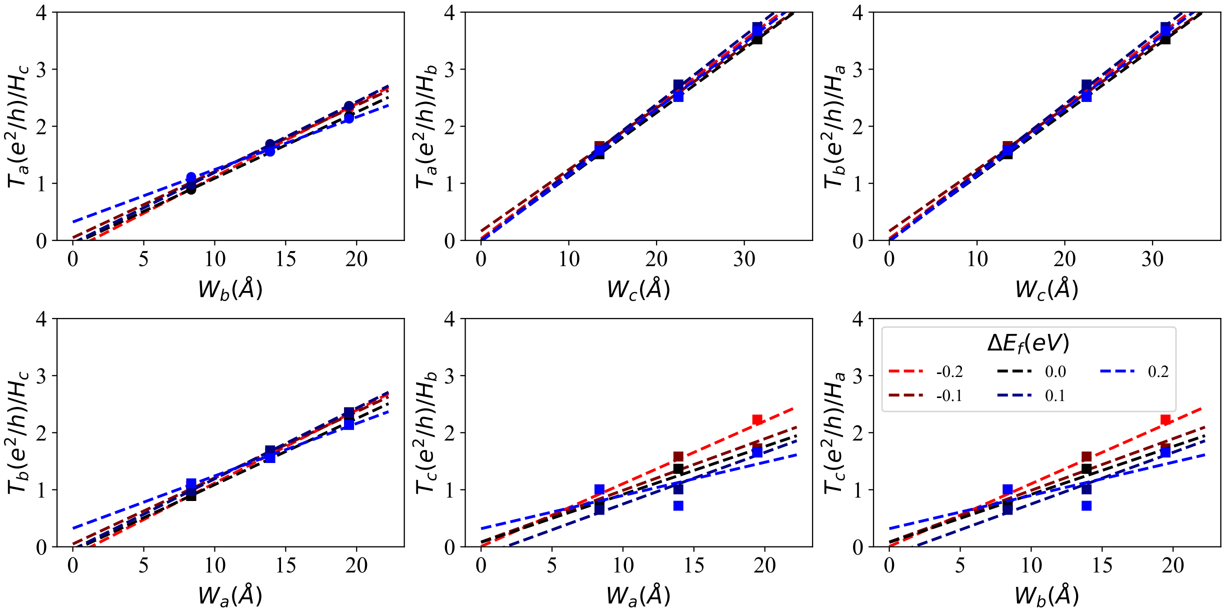

Compound: Ru
Materials Project ID: 33

Lattice (conventional cell):

Parameter	Value	Unit
a	2.7329	Å
b	2.7329	Å
c	4.3139	Å
α (alpha)	90.0000	°
β (beta)	90.0000	°
γ (gamma)	120.0000	°

Crystal structure (conventional cell):

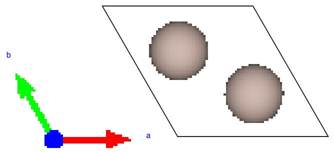

Nanowire Transmission:

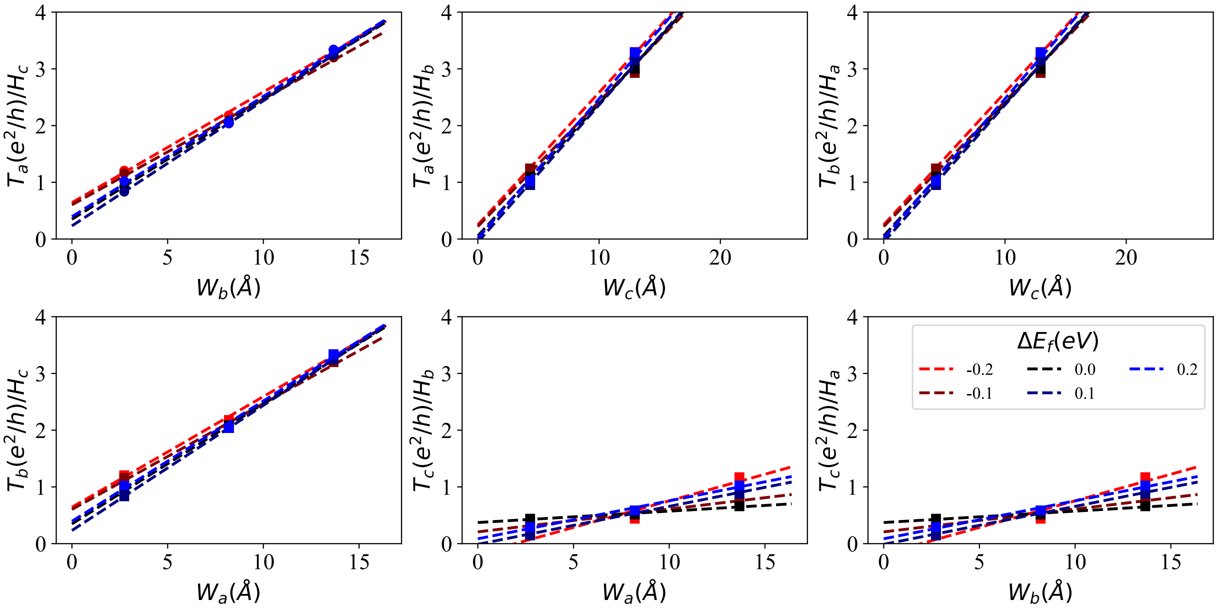

Compound: $ScGa_3$
Materials Project ID: 932

Lattice (conventional cell):

Parameter	Value	Unit
a	4.1216	Å
b	4.1216	Å
c	4.1216	Å
α (alpha)	90.0000	°
β (beta)	90.0000	°
γ (gamma)	90.0000	°

Crystal structure (conventional cell):

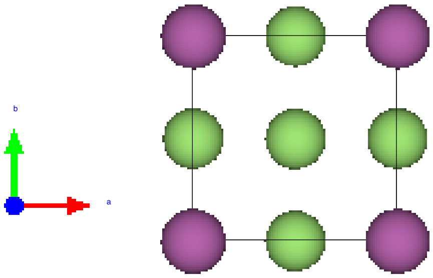

Nanowire Transmission:

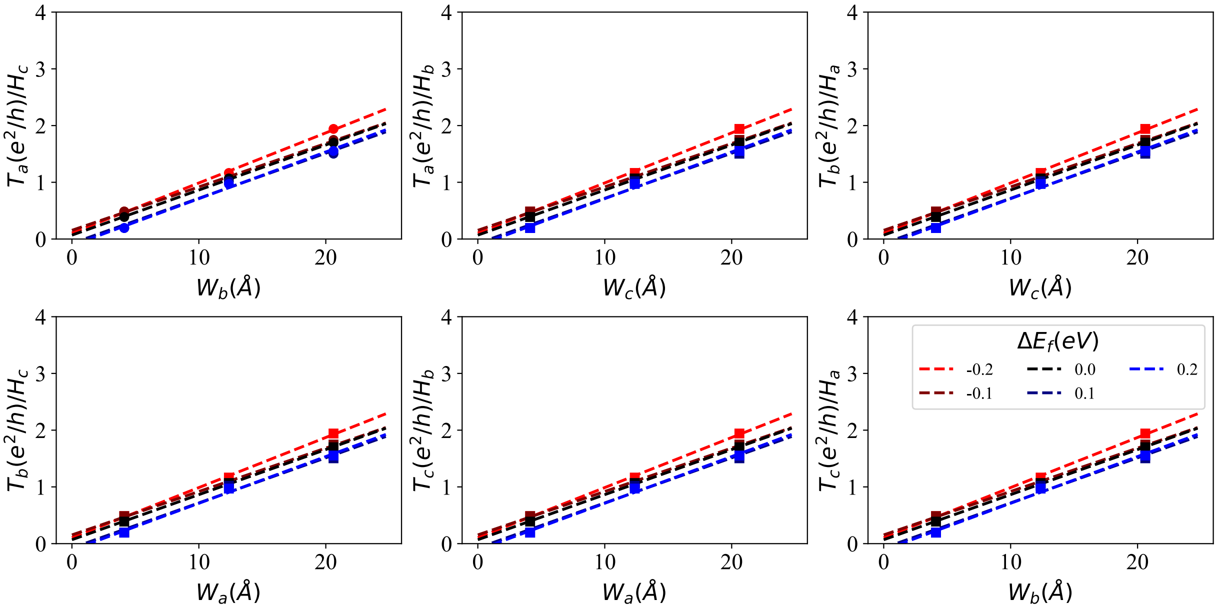

Compound: Se
Materials Project ID: 14

Lattice (conventional cell):

Parameter	Value	Unit
a	4.5195	Å
b	4.5195	Å
c	5.0500	Å
α (alpha)	90.0000	°
β (beta)	90.0000	°
γ (gamma)	120.0000	°

Crystal structure (conventional cell):

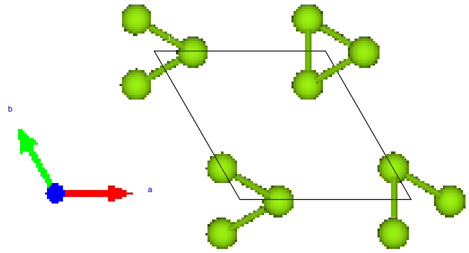

Nanowire Transmission:

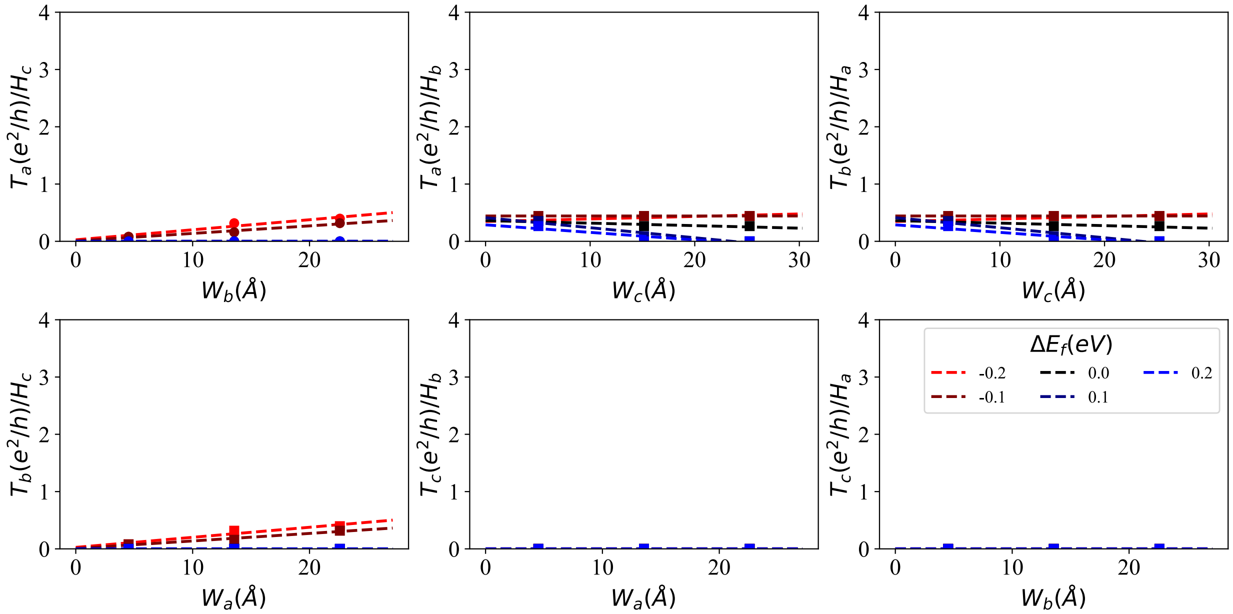

Compound: SiRh
Materials Project ID: 1483

Lattice (conventional cell):

Parameter	Value	Unit
a	4.7295	Å
b	4.7295	Å
c	4.7295	Å
α (alpha)	90.0000	°
β (beta)	90.0000	°
γ (gamma)	90.0000	°

Crystal structure (conventional cell):

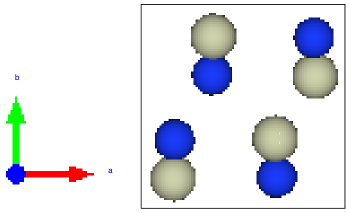

Nanowire Transmission:

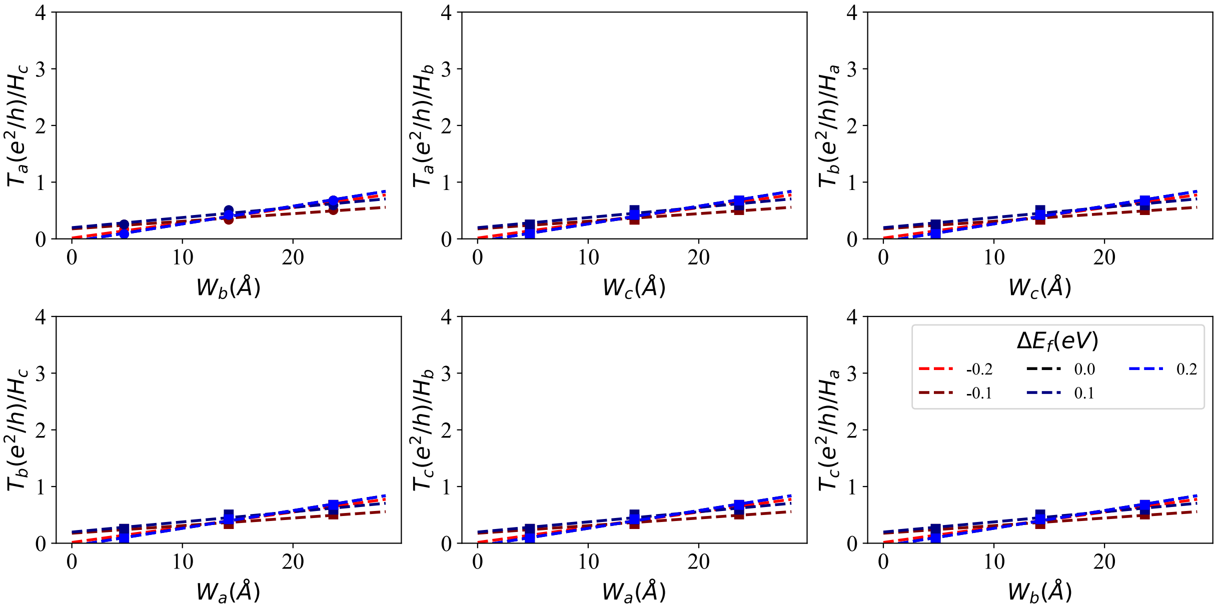

Compound: ScCuGe
Materials Project ID: 1078430

Lattice (conventional cell):

Parameter	Value	Unit
a	6.5606	Å
b	6.5606	Å
c	3.9977	Å
α (alpha)	90.0000	°
β (beta)	90.0000	°

Crystal structure (conventional cell):

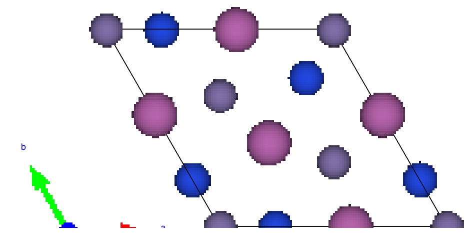

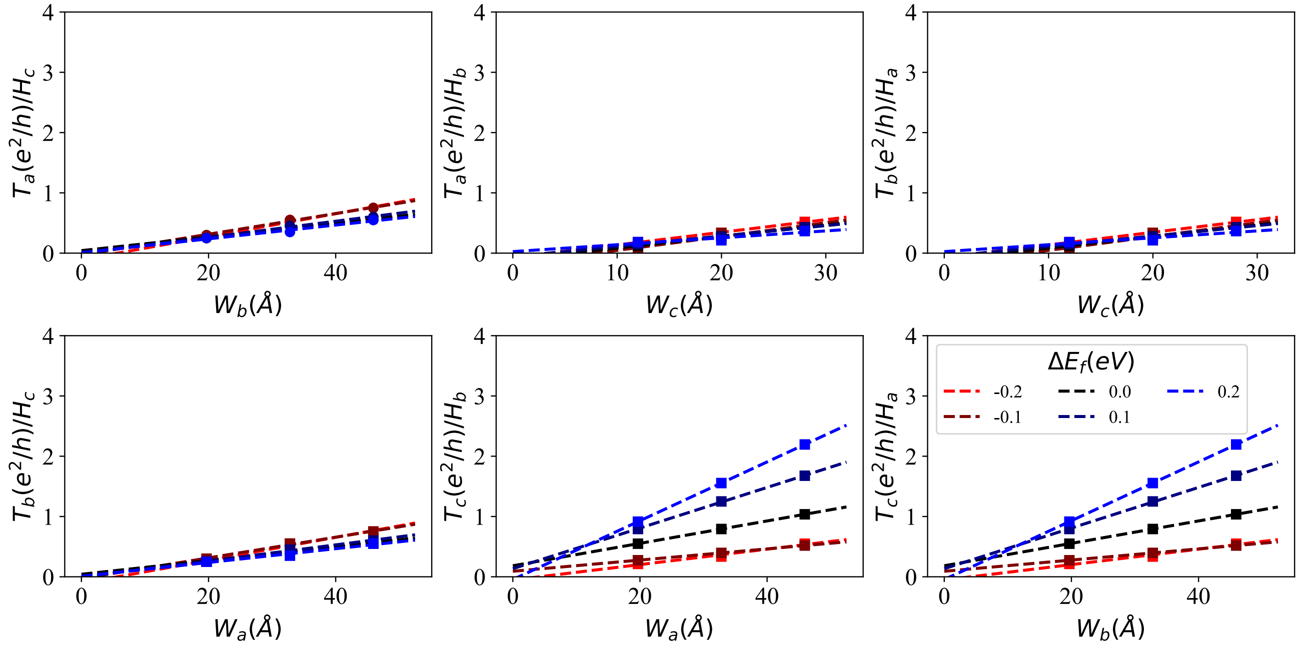

Compound: SnH_4
Materials Project ID: 1080725

Lattice (conventional cell):

Parameter	Value	Unit
a	3.9522	Å
b	3.9522	Å
c	6.4992	Å
α (alpha)	90.0000	°
β (beta)	90.0000	°
γ (gamma)	120.0000	°

Crystal structure (conventional cell):

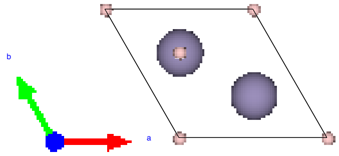

Nanowire Transmission:

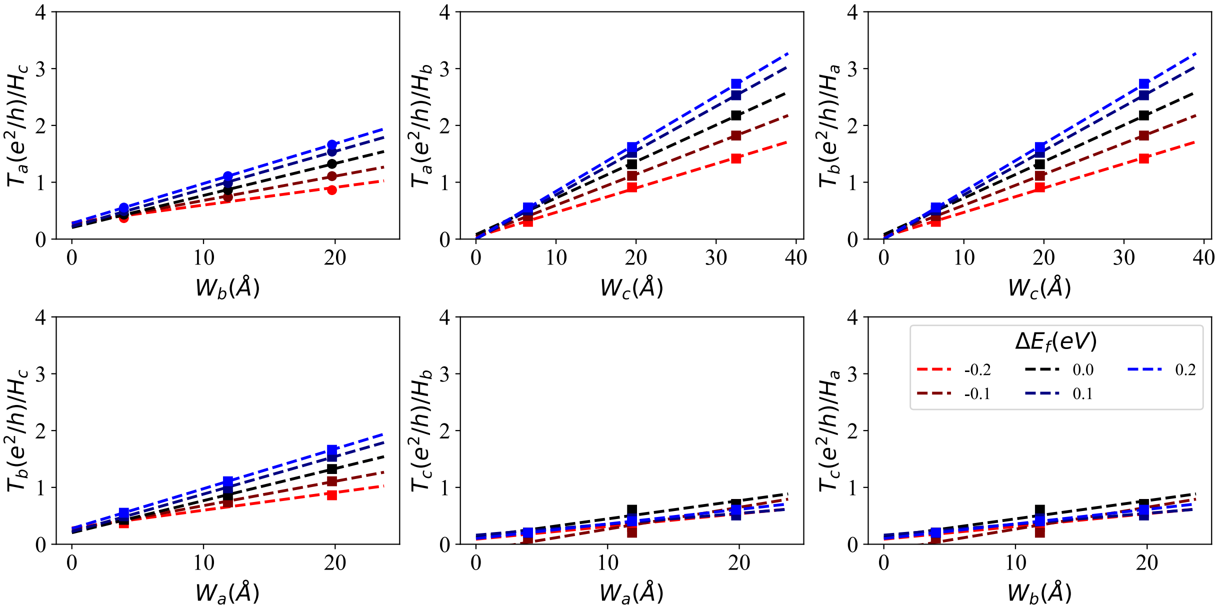

Compound: $Sn(HgSe_2)_2$
Materials Project ID: 10955

Lattice (conventional cell):

Parameter	Value	Unit
a	5.9365	Å
b	5.9365	Å
c	11.9940	Å
α (alpha)	90.0000	°
β (beta)	90.0000	°
γ (gamma)	90.0000	°

Crystal structure (conventional cell):

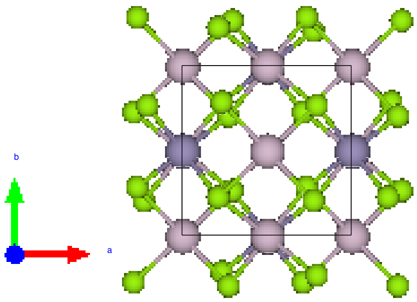

Nanowire Transmission:

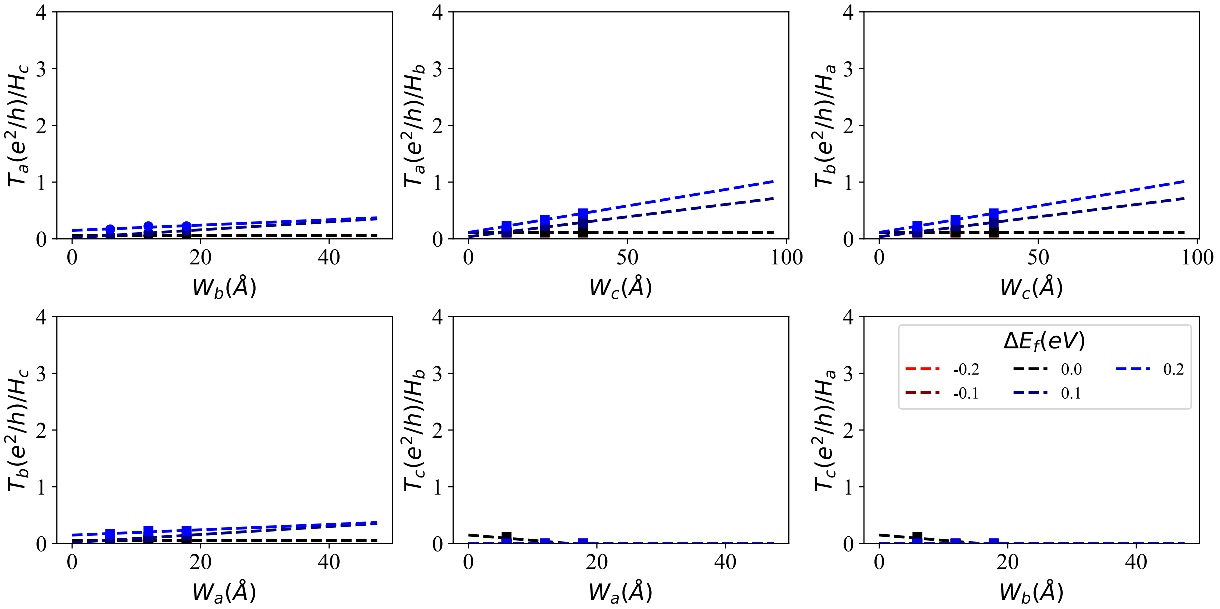

Compound: $Sr(CdSb)_2$
Materials Project ID: 7432

Lattice (conventional cell):

Parameter	Value	Unit
a	4.7972	Å
b	4.7972	Å
c	7.9056	Å
α (alpha)	90.0000	°
β (beta)	90.0000	°
γ (gamma)	120.0000	°

Crystal structure (conventional cell):

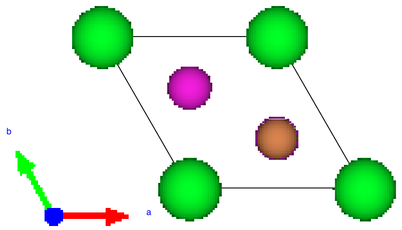

Nanowire Transmission:

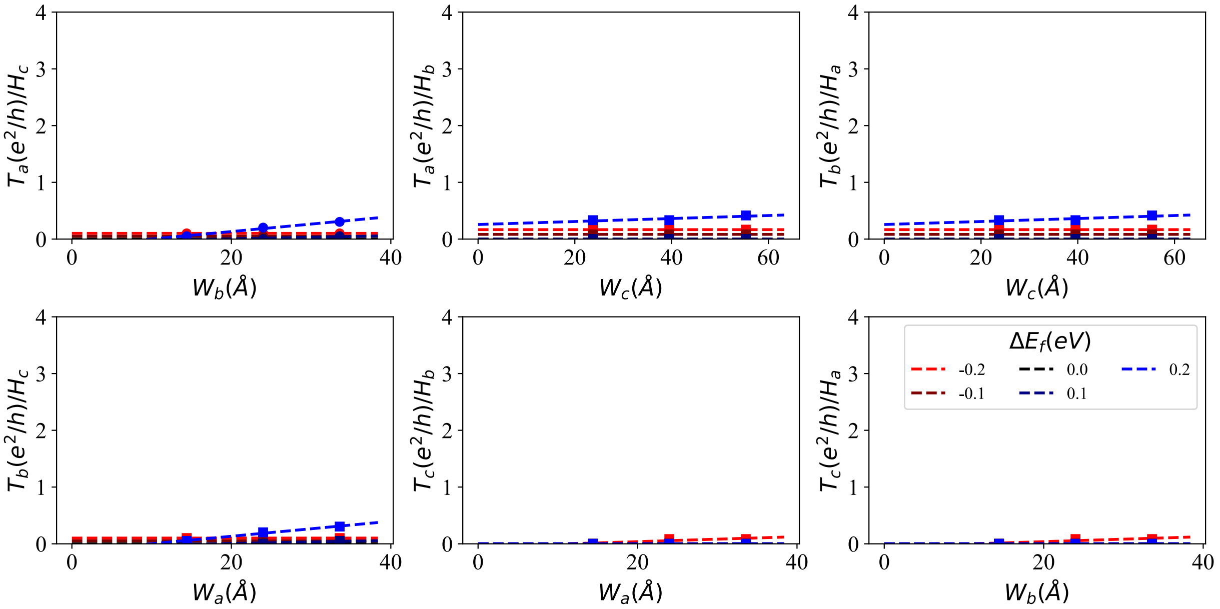

Compound: $Sr_3(AlGe)_2$
Materials Project ID: 571216

Lattice (conventional cell):

Parameter	Value	Unit
a	12.7411	Å
b	4.2166	Å
c	8.9551	Å
α (alpha)	90.0000	°
β (beta)	110.1782	°
γ (gamma)	90.0000	°

Crystal structure (conventional cell):

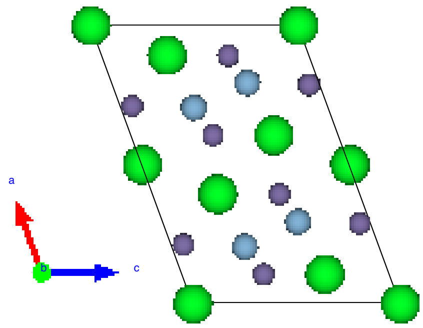

Nanowire Transmission:

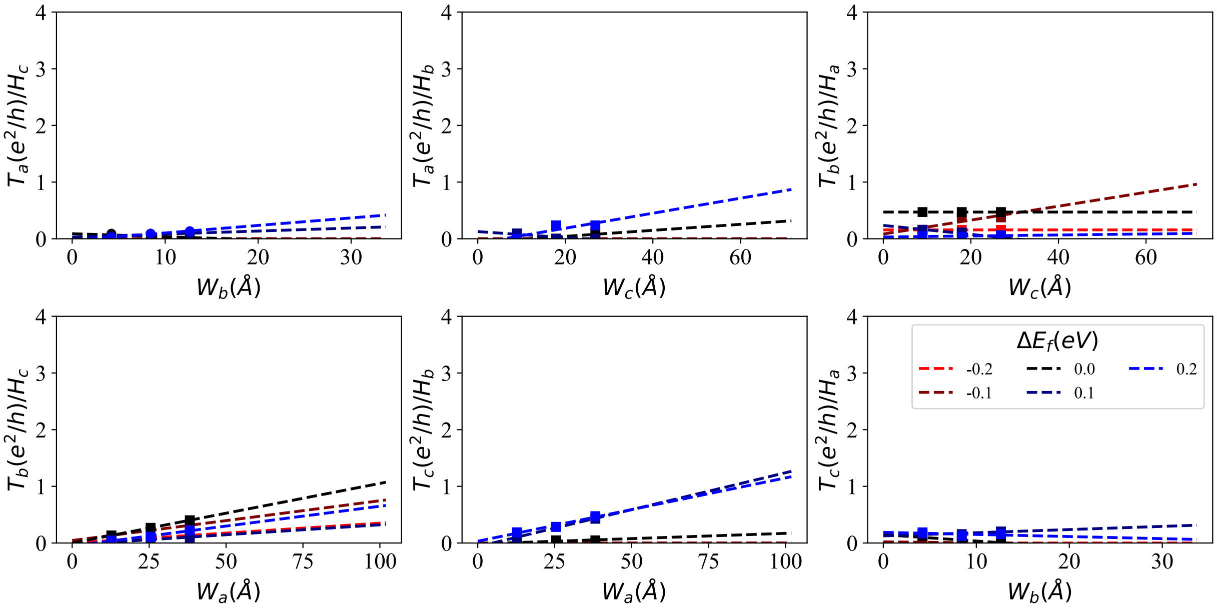

Compound: $SrAs_3$
Materials Project ID: 9907

Lattice (conventional cell):

Parameter	Value	Unit
a	9.7449	Å
b	7.7486	Å
c	5.9535	Å
α (alpha)	90.0000	°
β (beta)	112.9053	°
γ (gamma)	90.0000	°

Crystal structure (conventional cell):

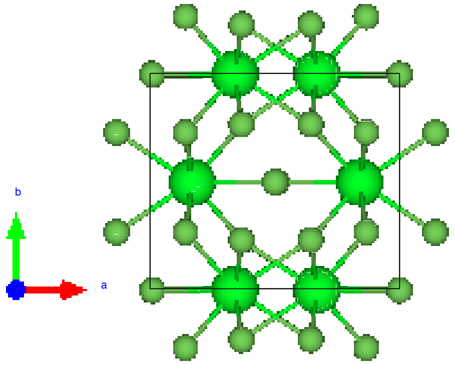

Nanowire Transmission:

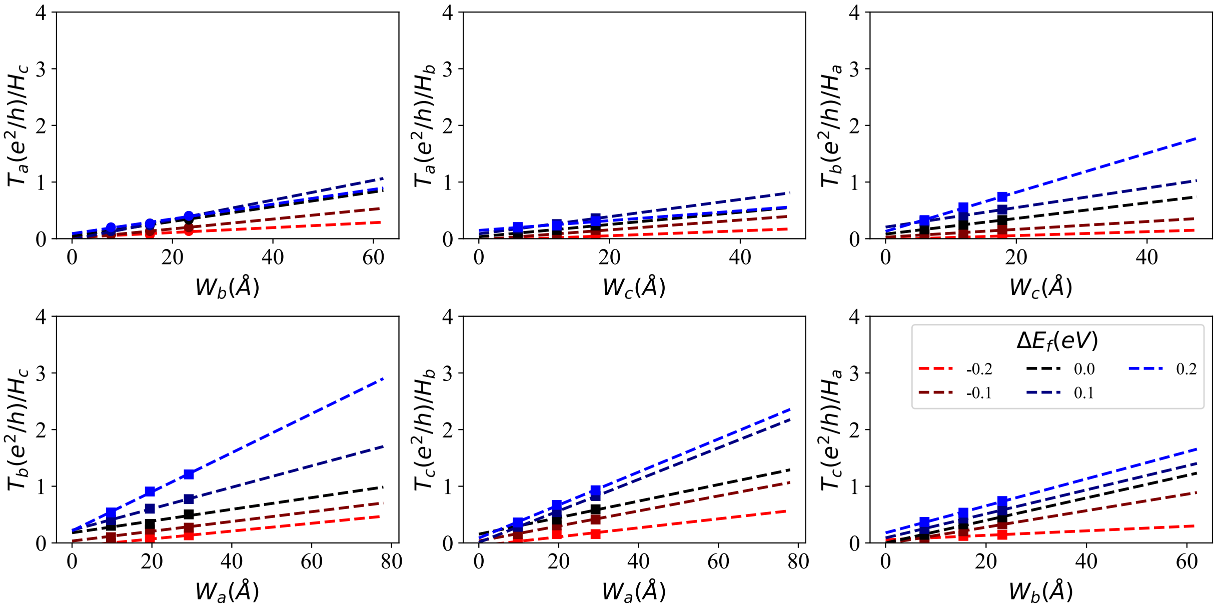

Compound: Sr_2Bi
Materials Project ID: 29619

Lattice (conventional cell):

Parameter	Value	Unit
a	5.1526	Å
b	5.1526	Å
c	18.1556	Å
α (alpha)	90.0000	°
β (beta)	90.0000	°
γ (gamma)	90.0000	°

Crystal structure (conventional cell):

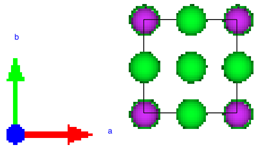

Nanowire Transmission:

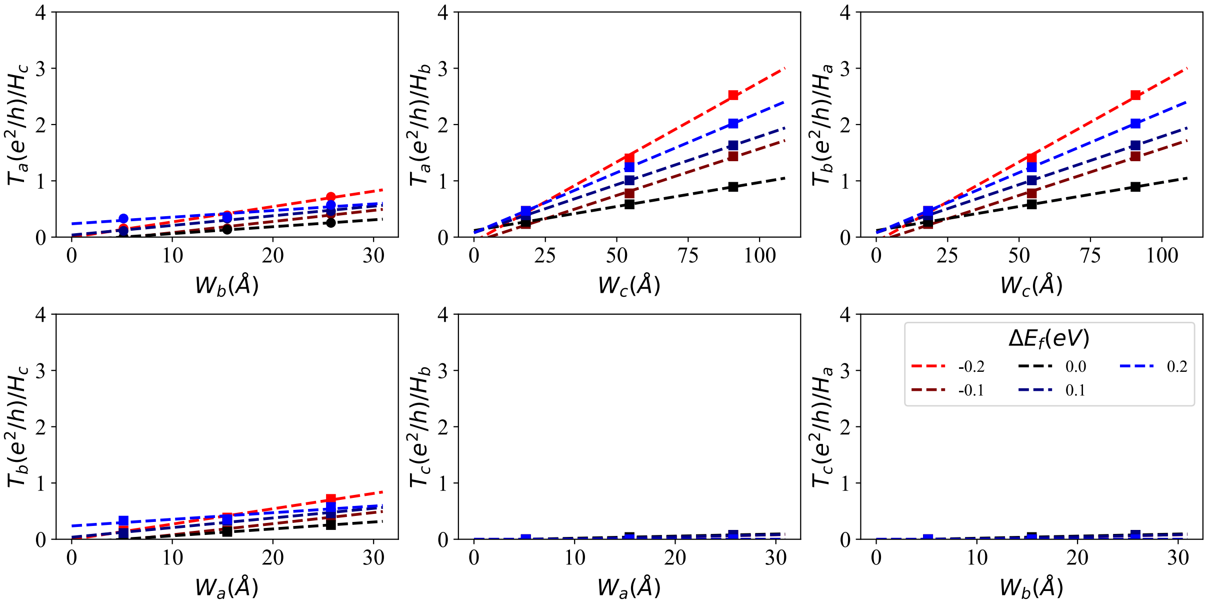

Compound: $SrGe_2$
Materials Project ID: 7390

Lattice (conventional cell):

Parameter	Value	Unit
a	4.1699	Å
b	4.1699	Å
c	5.1991	Å
α (alpha)	90.0000	°
β (beta)	90.0000	°
γ (gamma)	120.0000	°

Crystal structure (conventional cell):

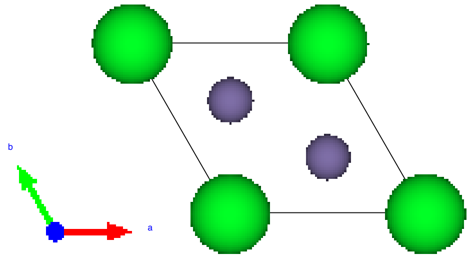

Nanowire Transmission:

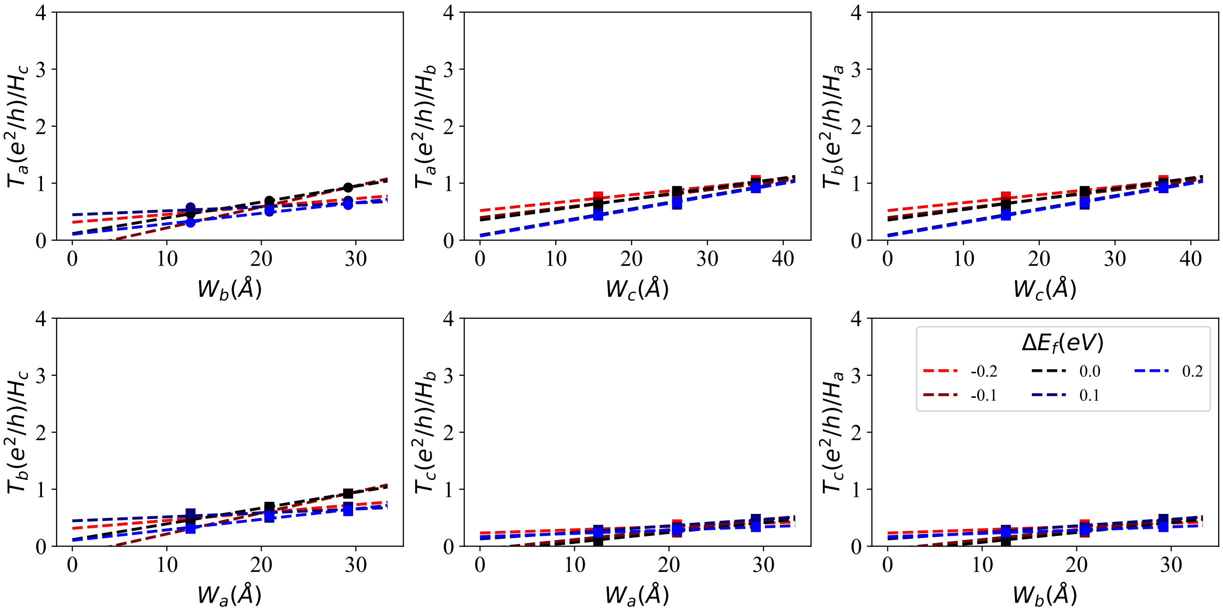

Compound: $SrMnSb_2$
Materials Project ID: 1079721

Lattice (conventional cell):

Parameter	Value	Unit
a	4.4170	Å
b	4.4170	Å
c	11.7201	Å
α (alpha)	90.0000	°
β (beta)	90.0000	°
γ (gamma)	90.0000	°

Crystal structure (conventional cell):

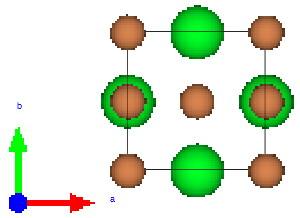

Nanowire Transmission:

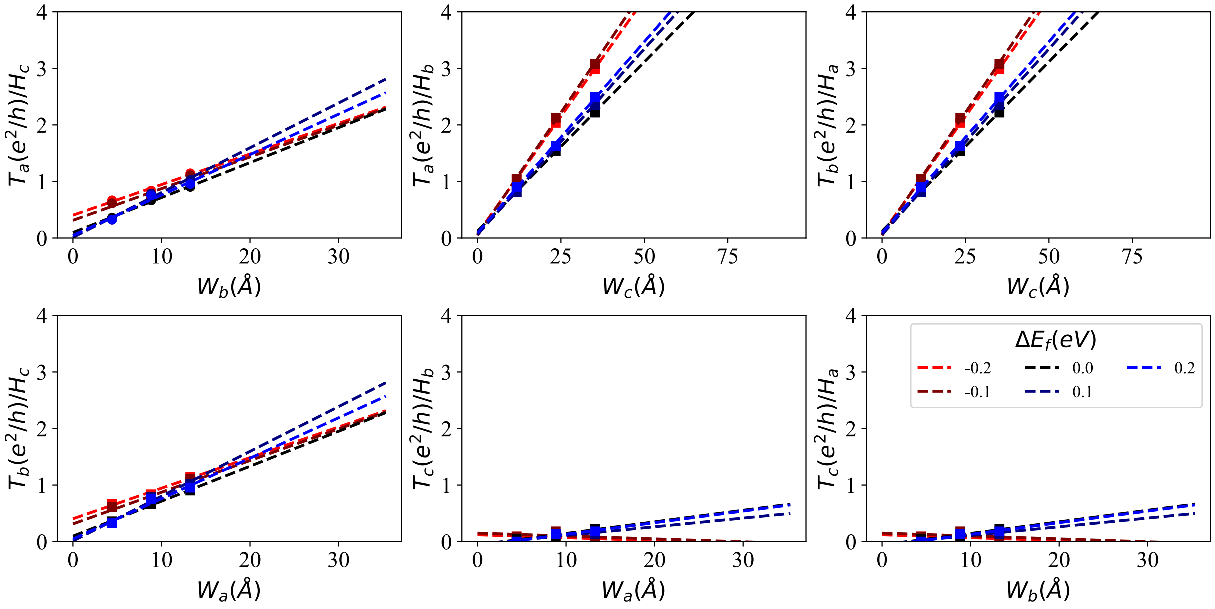

Compound: $SrSi_2$
Materials Project ID: 1727

Lattice (conventional cell):

Parameter	Value	Unit
a	4.4377	Å
b	4.4377	Å
c	13.9661	Å
α (alpha)	90.0000	°
β (beta)	90.0000	°
γ (gamma)	90.0000	°

Crystal structure (conventional cell):

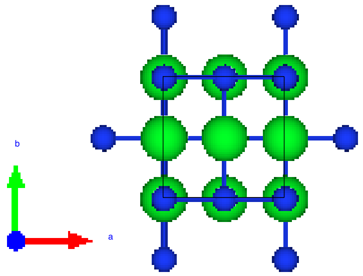

Nanowire Transmission:

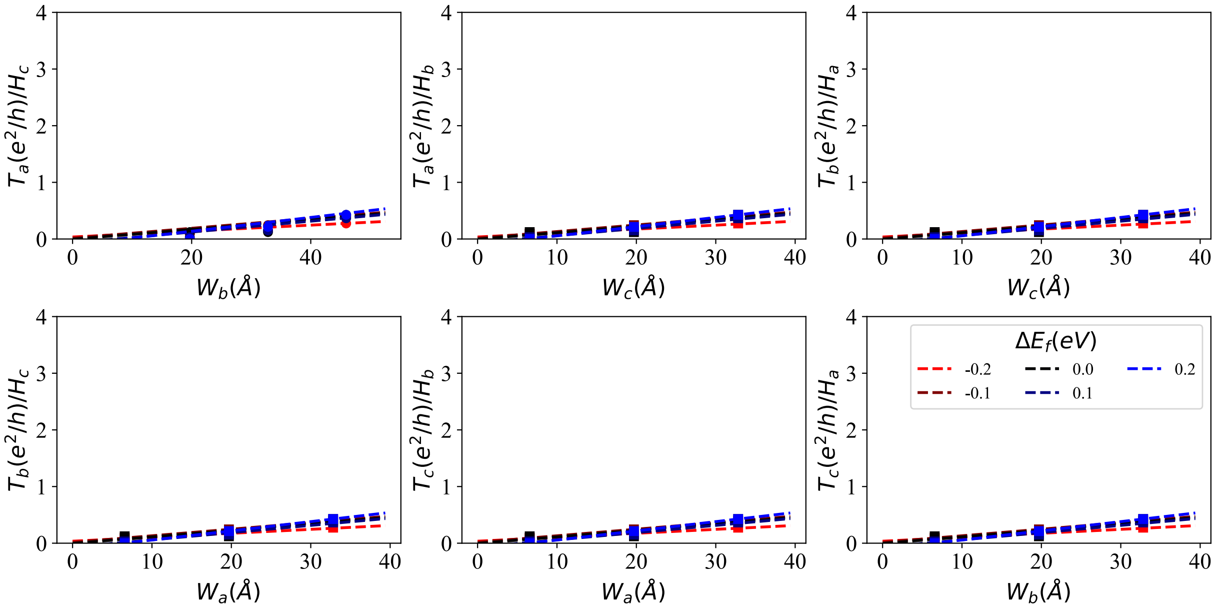

Compound: SrSnHg
Materials Project ID: 1019259

Lattice (conventional cell):

Parameter	Value	Unit
a	5.0060	Å
b	5.0060	Å
c	8.3087	Å
α (alpha)	90.0000	°
β (beta)	90.0000	°
γ (gamma)	120.0000	°

Crystal structure (conventional cell):

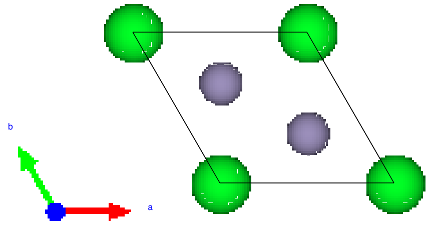

Nanowire Transmission:

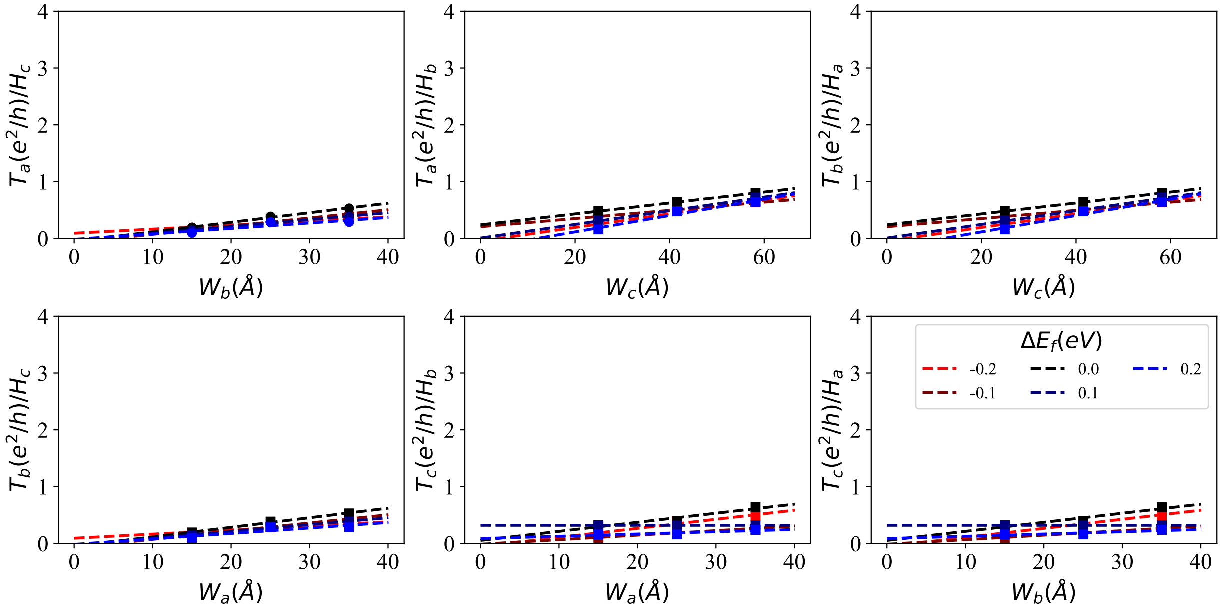

Compound: TaAs
Materials Project ID: 1936

Lattice (conventional cell):

Parameter	Value	Unit
a	3.4696	Å
b	3.4696	Å
c	11.7349	Å
α (alpha)	90.0000	°
β (beta)	90.0000	°
γ (gamma)	90.0000	°

Crystal structure (conventional cell):

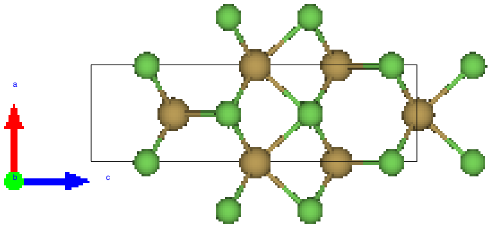

Nanowire Transmission:

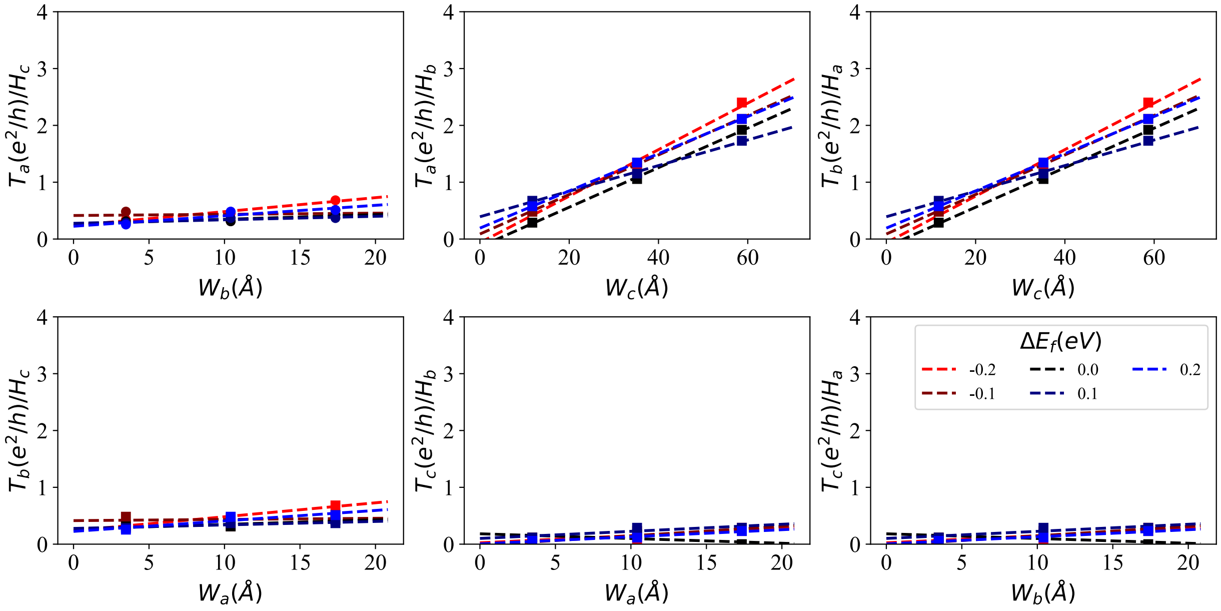

Compound: $TaAs_2$
Materials Project ID: 12561

Lattice (conventional cell):

Parameter	Value	Unit
a	9.4399	Å
b	3.4259	Å
c	7.8478	Å
α (alpha)	90.0000	°
β (beta)	119.7016	°
γ (gamma)	90.0000	°

Crystal structure (conventional cell):

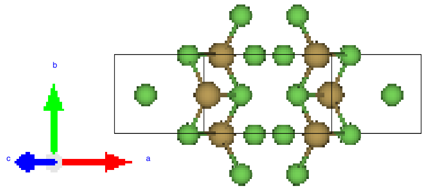

Nanowire Transmission:

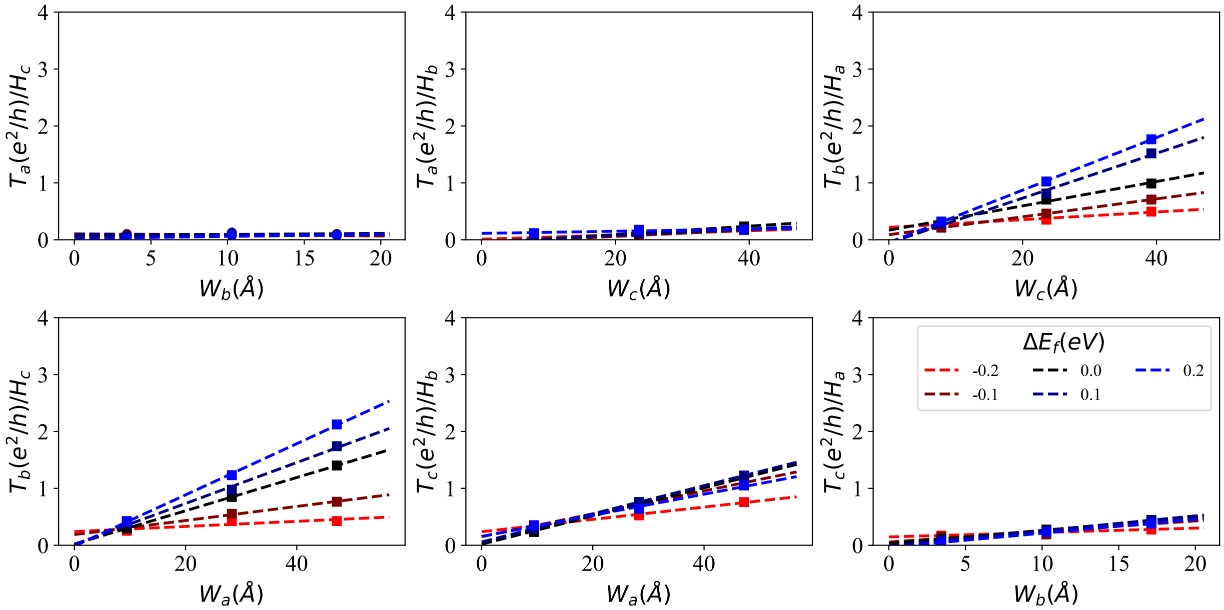

Compound: $TaInS_2$
Materials Project ID: 22332

Lattice (conventional cell):

Parameter	Value	Unit
a	3.3249	Å
b	3.3249	Å
c	8.9364	Å
α (alpha)	90.0000	°
β (beta)	90.0000	°
γ (gamma)	120.0000	°

Crystal structure (conventional cell):

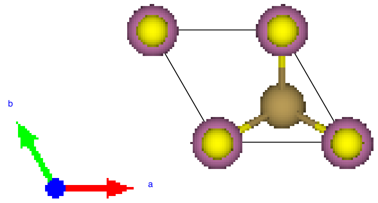

Nanowire Transmission:

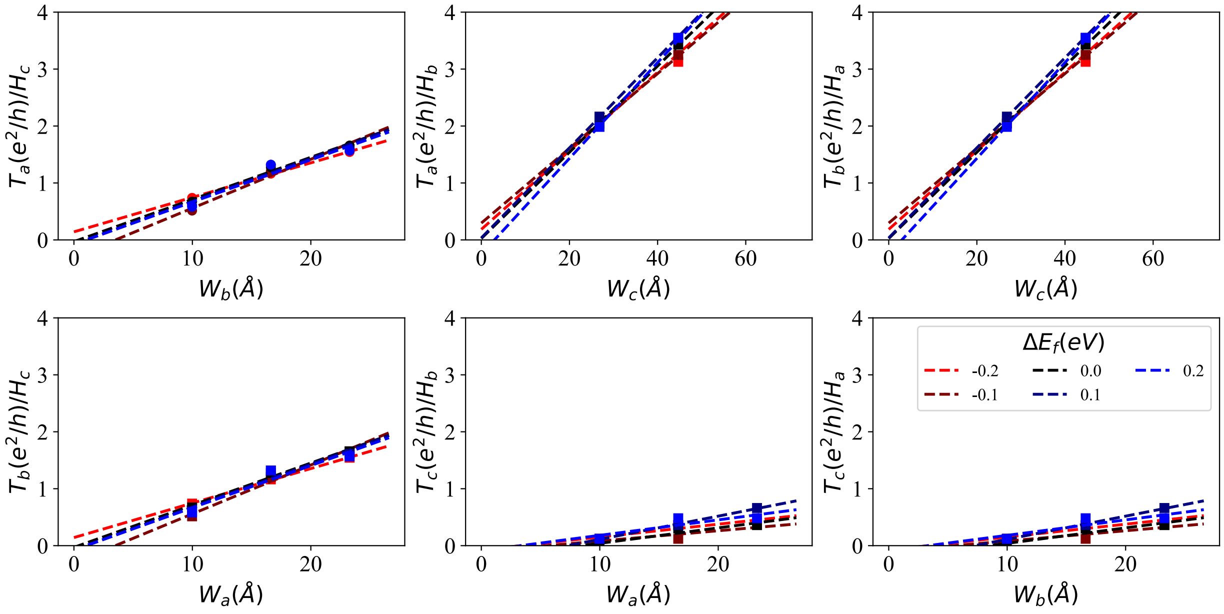

Compound: TaN
Materials Project ID: 1459

Lattice (conventional cell):

Parameter	Value	Unit
a	2.9540	Å
b	2.9540	Å
c	2.9015	Å
α (alpha)	90.0000	°
β (beta)	90.0000	°
γ (gamma)	120.0000	°

Crystal structure (conventional cell):

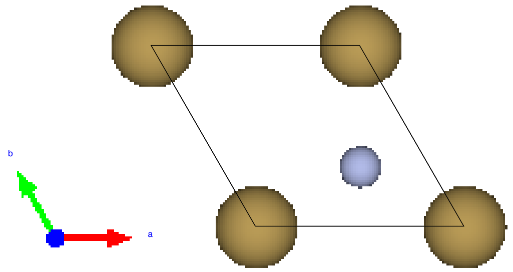

Nanowire Transmission:

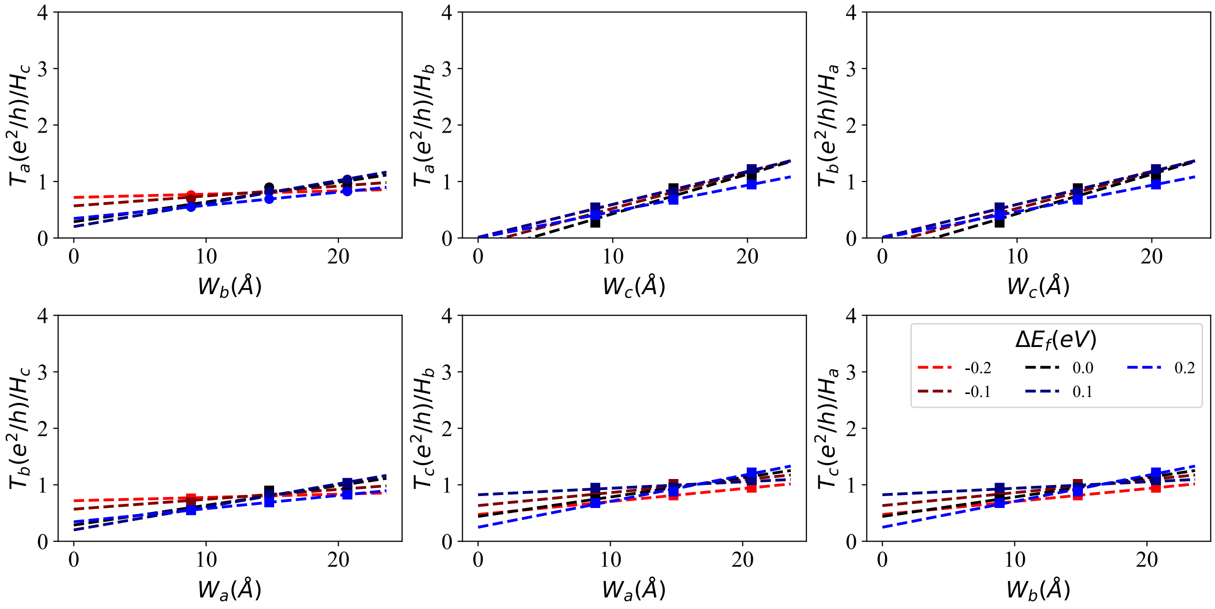

Compound: TaP
Materials Project ID: 1067587

Lattice (conventional cell):

Parameter	Value	Unit
a	3.3402	Å
b	3.3402	Å
c	11.4036	Å
α (alpha)	90.0000	°
β (beta)	90.0000	°
γ (gamma)	90.0000	°

Crystal structure (conventional cell):

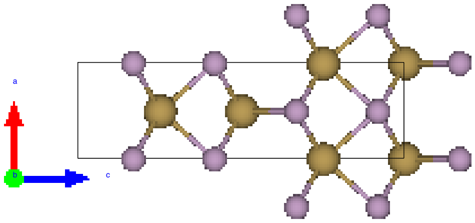

Nanowire Transmission:

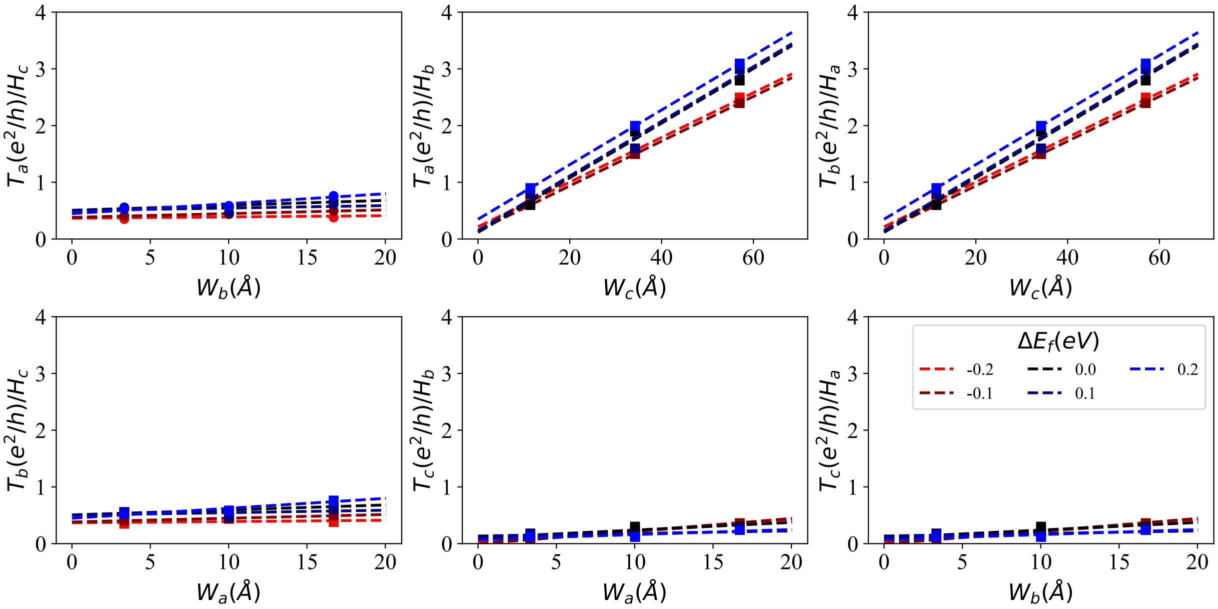

Compound: $TaPbSe_2$
Materials Project ID: 567736

Lattice (conventional cell):

Parameter	Value	Unit
a	3.4835	Å
b	3.4835	Å
c	9.4755	Å
α (alpha)	90.0000	°
β (beta)	90.0000	°
γ (gamma)	120.0000	°

Crystal structure (conventional cell):

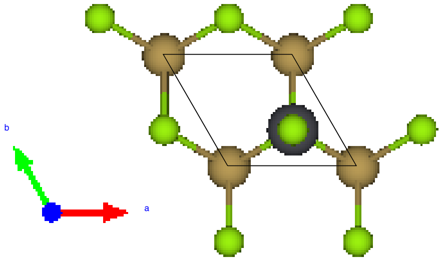

Nanowire Transmission:

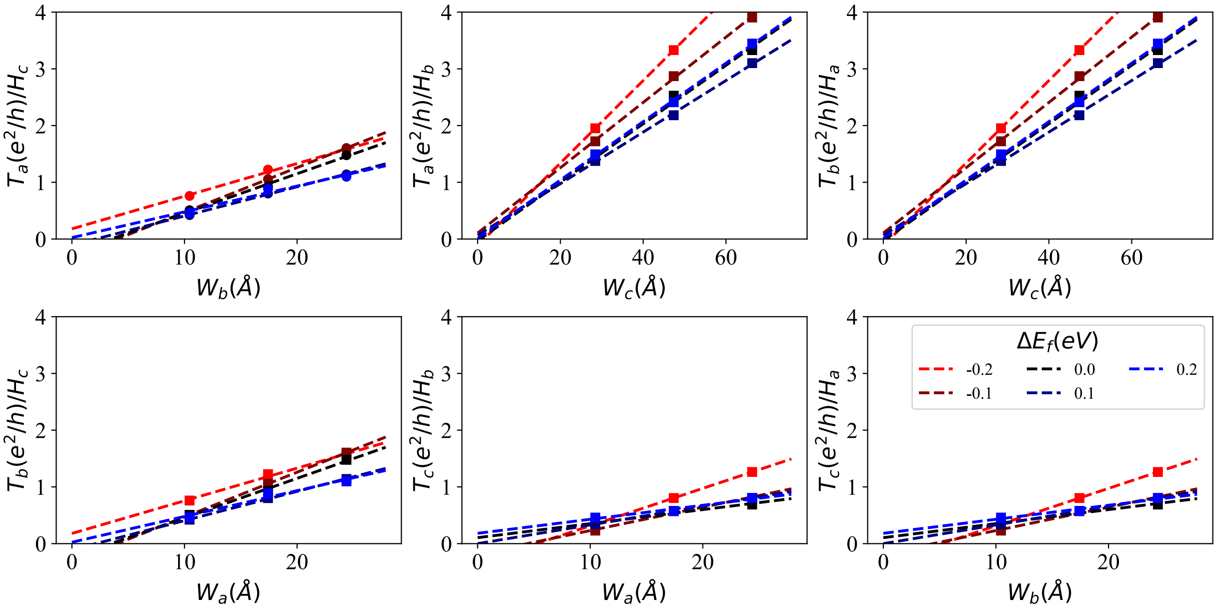

Compound: TaSiAs
Materials Project ID: 81

Lattice (conventional cell):

Parameter	Value	Unit
a	3.5206	Å
b	3.5206	Å
c	7.9142	Å
α (alpha)	90.0000	°
β (beta)	90.0000	°
γ (gamma)	90.0000	°

Crystal structure (conventional cell):

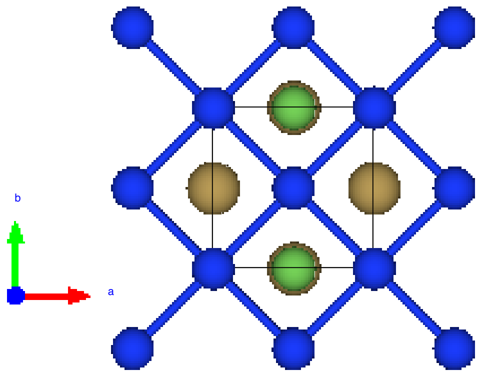

Nanowire Transmission:

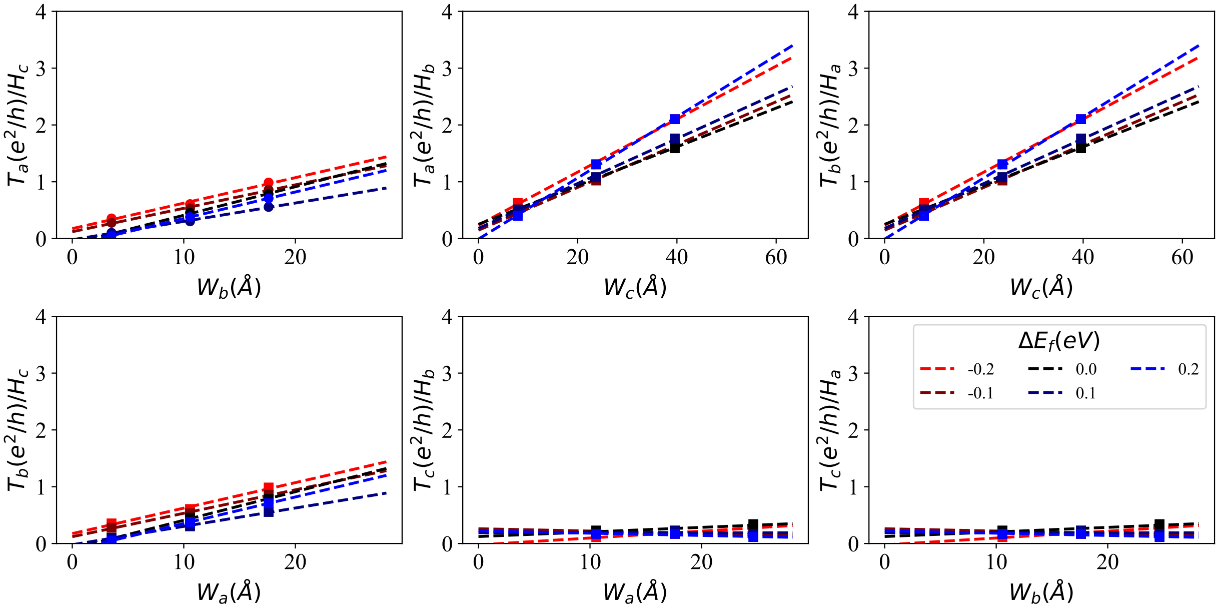

Compound: Te
Materials Project ID: 19

Lattice (conventional cell):

Parameter	Value	Unit
a	4.5124	Å
b	4.5124	Å
c	5.9599	Å
α (alpha)	90.0000	°
β (beta)	90.0000	°
γ (gamma)	120.0000	°

Crystal structure (conventional cell):

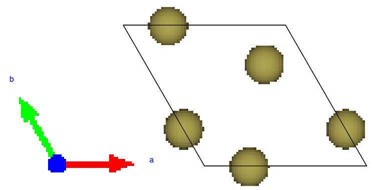

Nanowire Transmission:

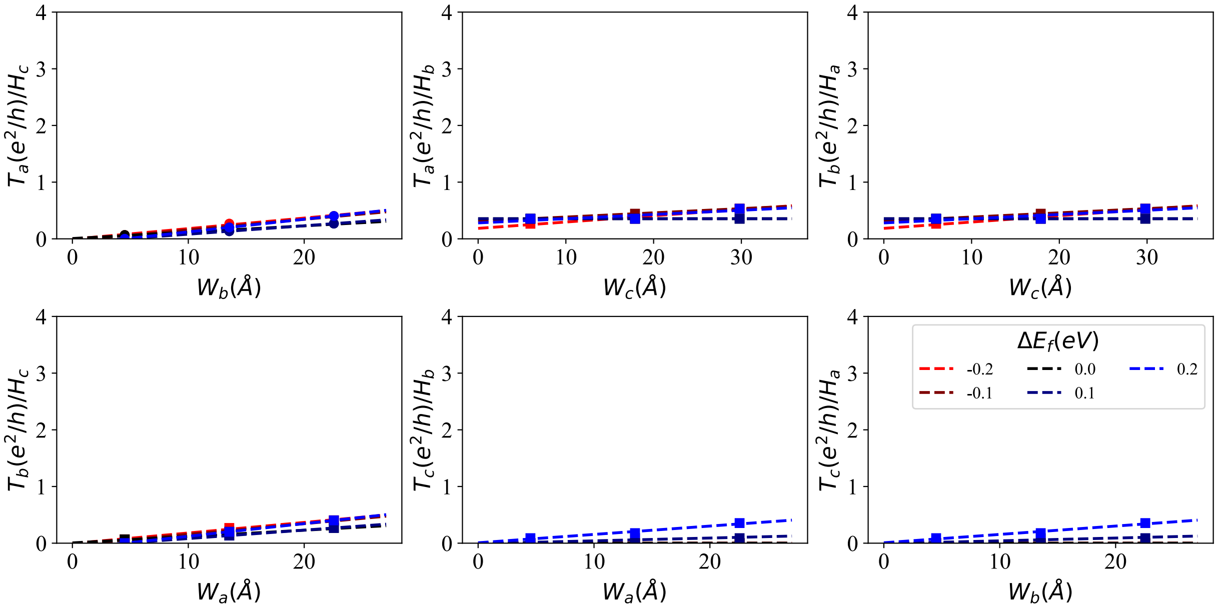

Compound: Te_2Ir
Materials Project ID: 2285

Lattice (conventional cell):

Parameter	Value	Unit
a	3.9878	Å
b	3.9878	Å
c	5.5433	Å
α (alpha)	90.0000	°
β (beta)	90.0000	°
γ (gamma)	120.0000	°

Crystal structure (conventional cell):

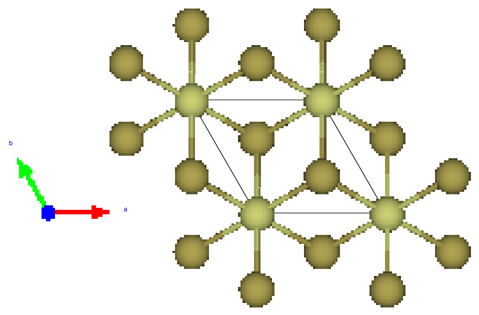

Nanowire Transmission:

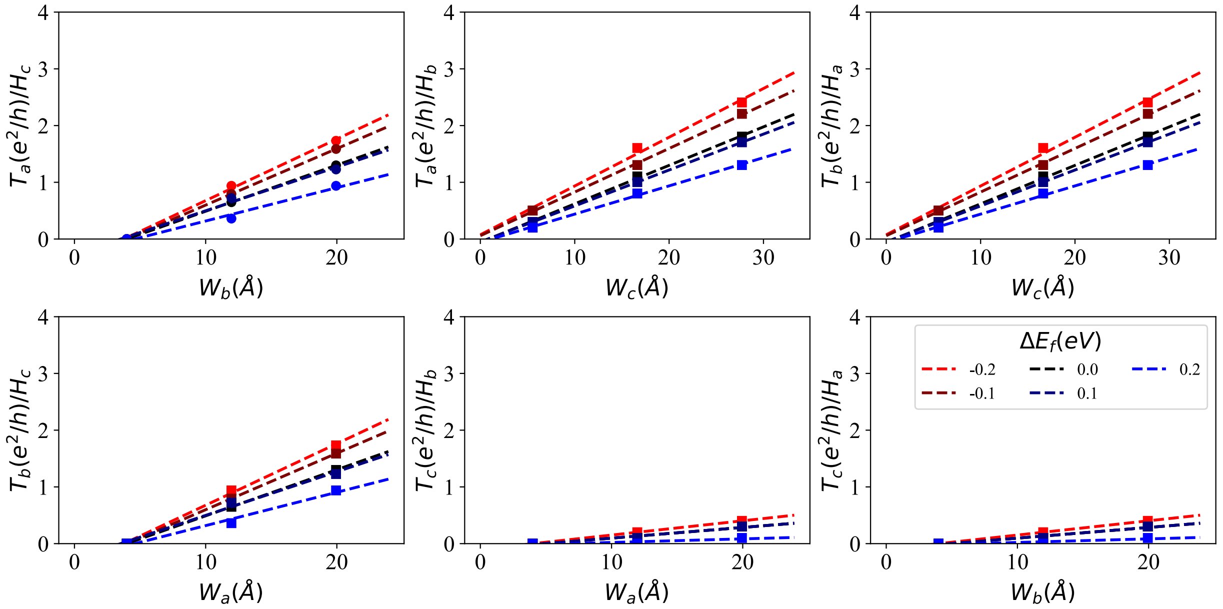

Compound: Te_2Mo
Materials Project ID: 7459

Lattice (conventional cell):

Parameter	Value	Unit
a	6.3662	Å
b	3.4869	Å
c	15.5479	Å
α (alpha)	90.0000	°
β (beta)	92.3879	°
γ (gamma)	90.0000	°

Crystal structure (conventional cell):

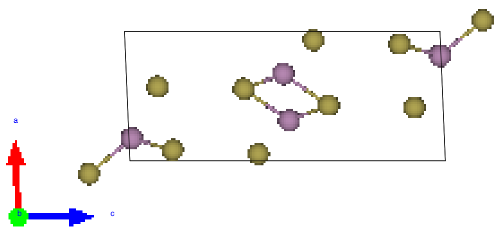

Nanowire Transmission:

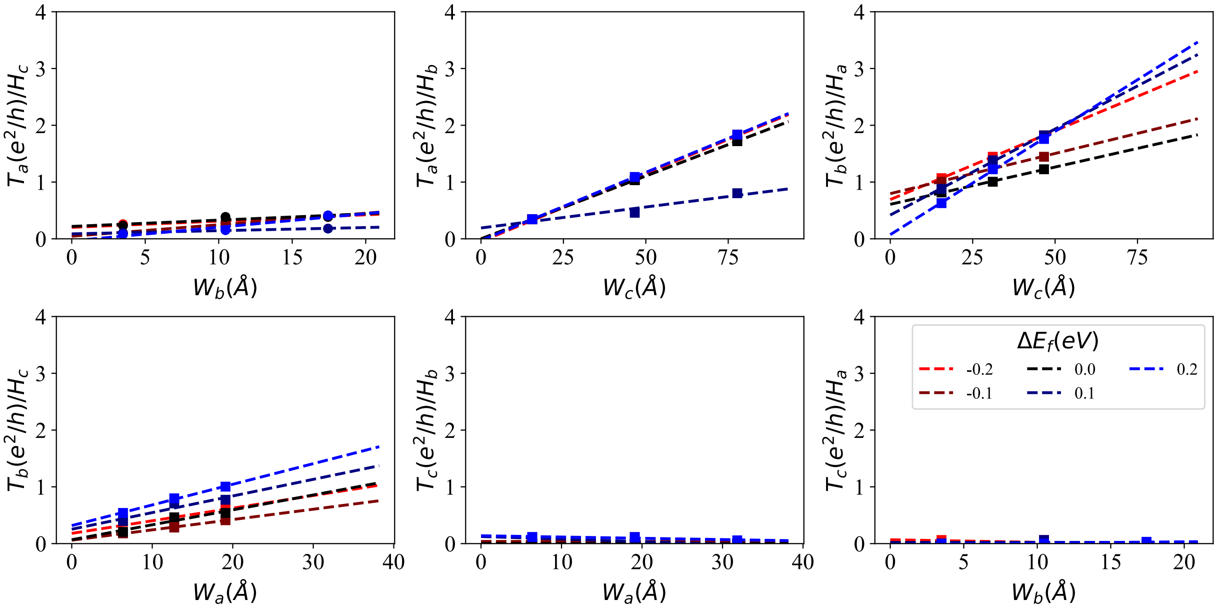

Compound: Te_2W
Materials Project ID:22693

Lattice (conventional cell):

Parameter	Value	Unit
a	3.4979	Å
b	6.3383	Å
c	15.4319	Å
α (alpha)	90.0000	°
β (beta)	90.0000	°
γ (gamma)	90.0000	°

Crystal structure (conventional cell):

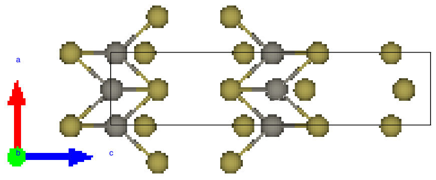

Nanowire Transmission:

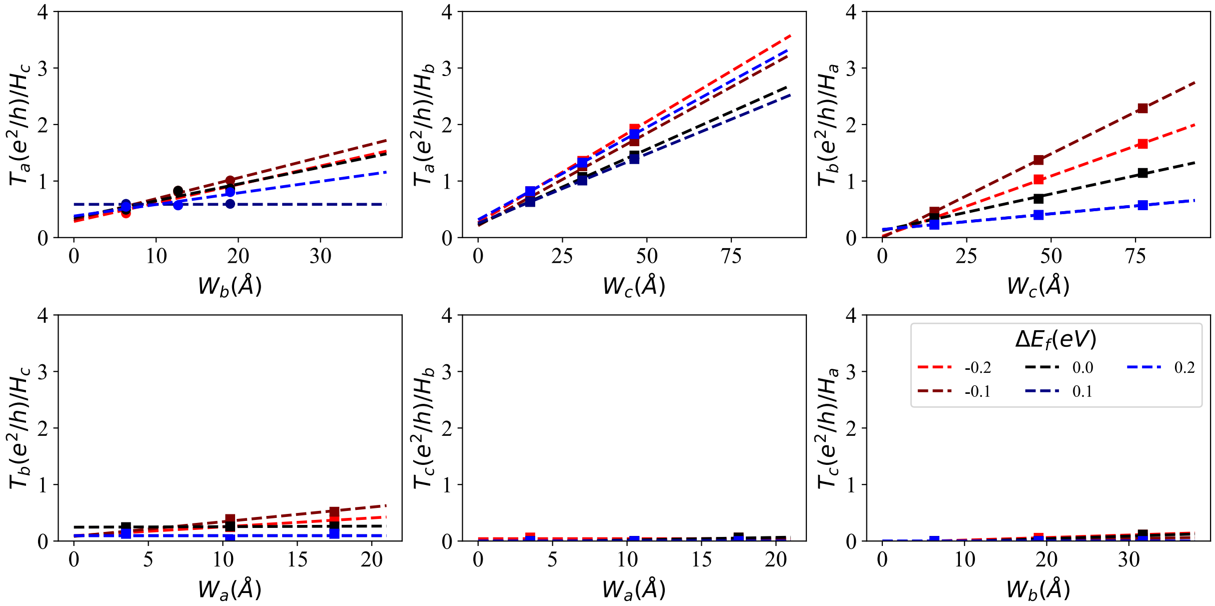

Compound: TiB_2
Materials Project ID: 1145

Lattice (conventional cell):

Parameter	Value	Unit
a	3.0351	Å
b	3.0351	Å
c	3.2232	Å
α (alpha)	90.0000	°
β (beta)	90.0000	°
γ (gamma)	120.0000	°

Crystal structure (conventional cell):

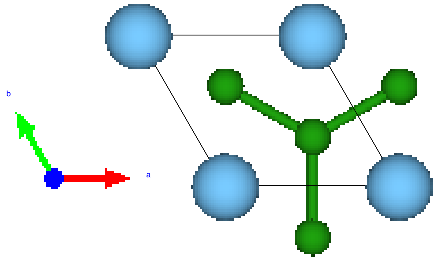

Nanowire Transmission:

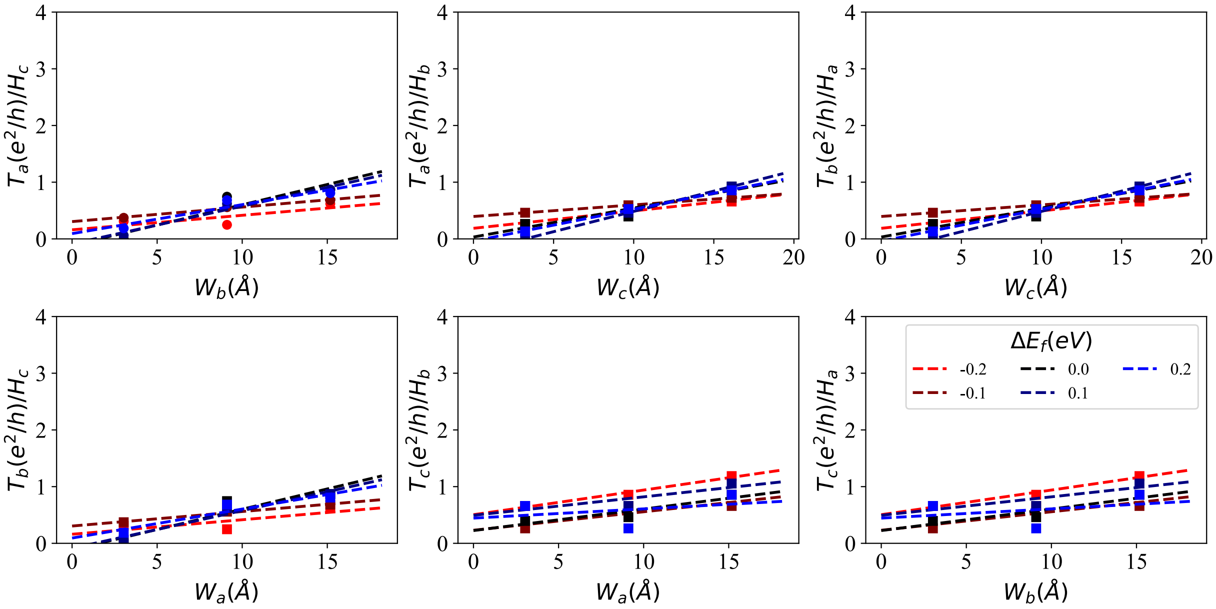

Compound: $TiCu_2$
Materials Project ID: 1077023

Lattice (conventional cell):

Parameter	Value	Unit
a	4.5123	Å
b	7.9862	Å
c	4.4641	Å
α (alpha)	90.0000	°
β (beta)	90.0000	°
γ (gamma)	90.0000	°

Crystal structure (conventional cell):

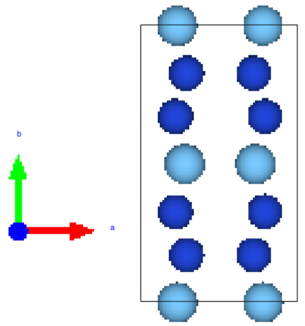

Nanowire Transmission:

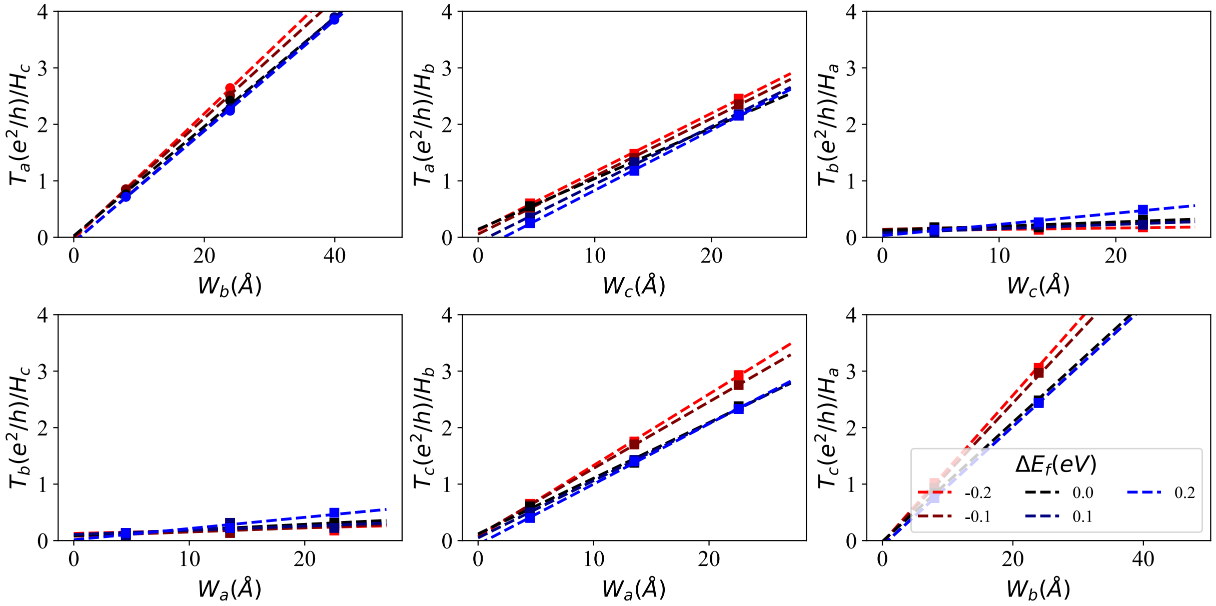

Compound: TiS
Materials Project ID: 1018028

Lattice (conventional cell):

Parameter	Value	Unit
a	3.2739	Å
b	3.2739	Å
c	3.2191	Å
α (alpha)	90.0000	°
β (beta)	90.0000	°
γ (gamma)	120.0000	°

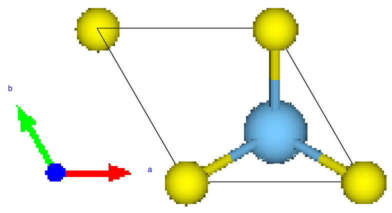

Nanowire Transmission:

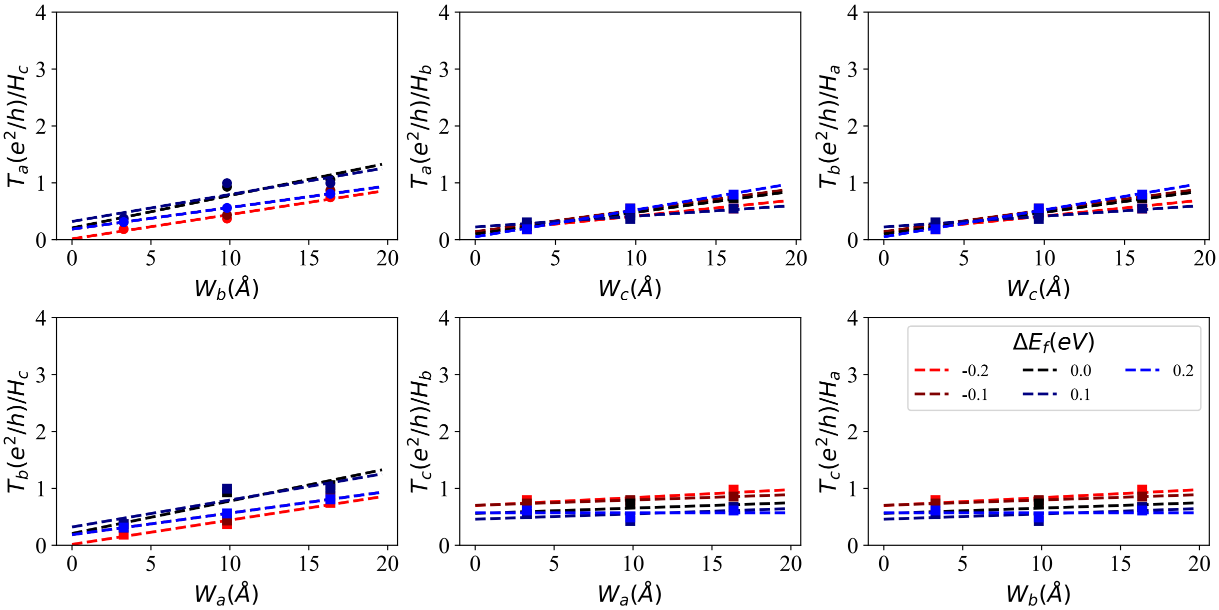

Compound: WC
Materials Project ID: 1894

Lattice (conventional cell):

Parameter	Value	Unit
a	2.9283	Å
b	2.9283	Å
c	2.8529	Å
α (alpha)	90.0000	°
β (beta)	90.0000	°
γ (gamma)	120.0000	°

Crystal structure (conventional cell):

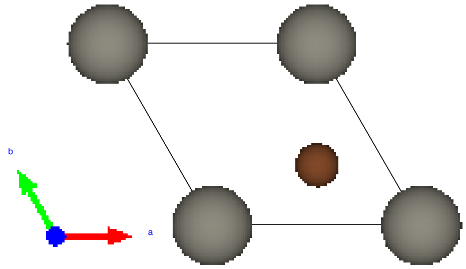

Nanowire Transmission:

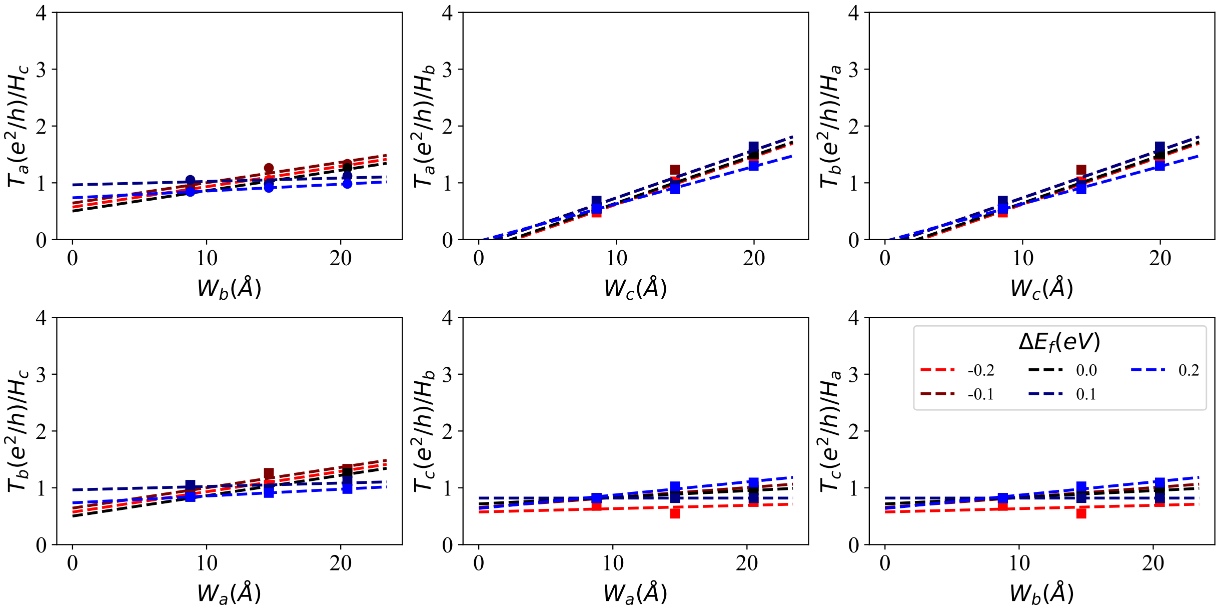

Compound: WN
Materials Project ID: 991

Lattice (conventional cell):

Parameter	Value	Unit
a	2.8760	Å
b	2.8760	Å
c	2.9080	Å
α (alpha)	90.0000	°
β (beta)	90.0000	°
γ (gamma)	120.0000	°

Crystal structure (conventional cell):

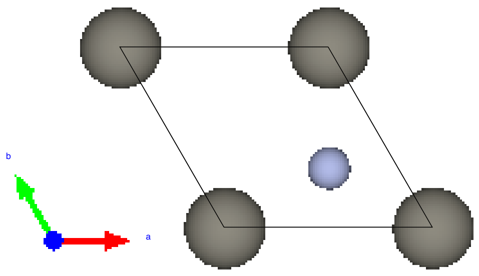

Nanowire Transmission:

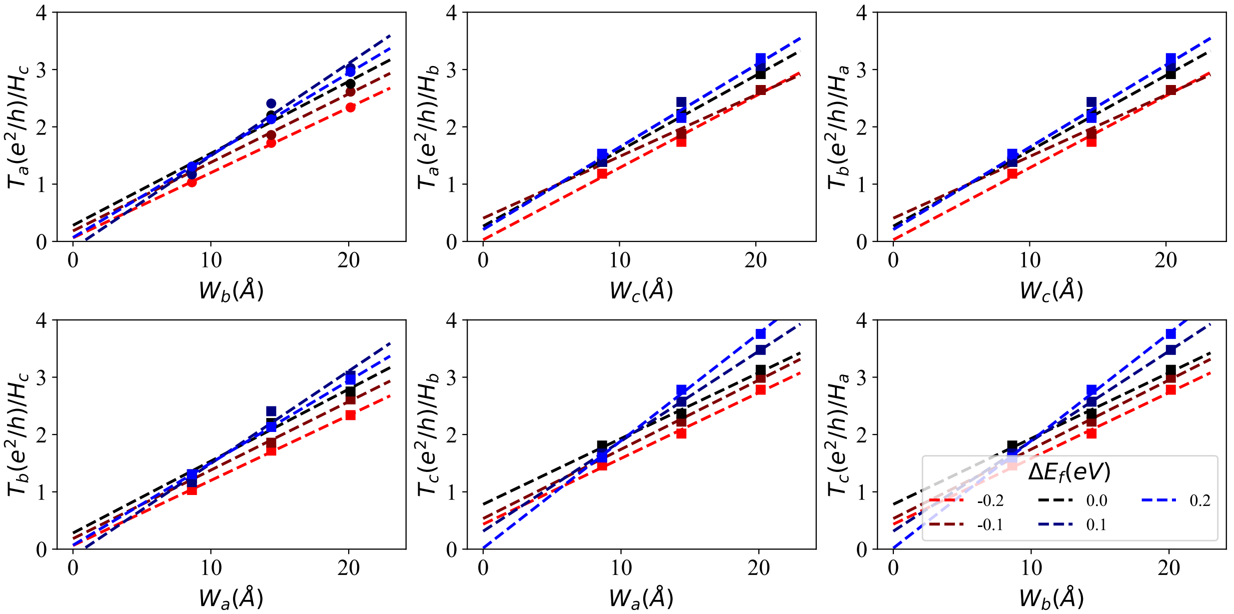

Compound: YAgPb
Materials Project ID: 21505

Lattice (conventional cell):

Parameter	Value	Unit
a	7.5818	Å
b	7.5818	Å
c	4.5605	Å
α (alpha)	90.0000	°
β (beta)	90.0000	°
γ (gamma)	120.0000	°

Crystal structure (conventional cell):

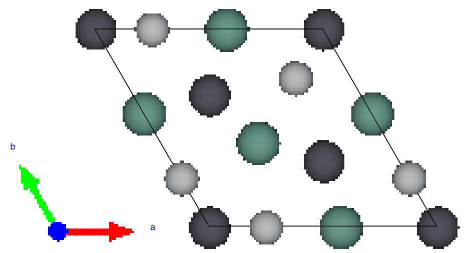

Nanowire Transmission:

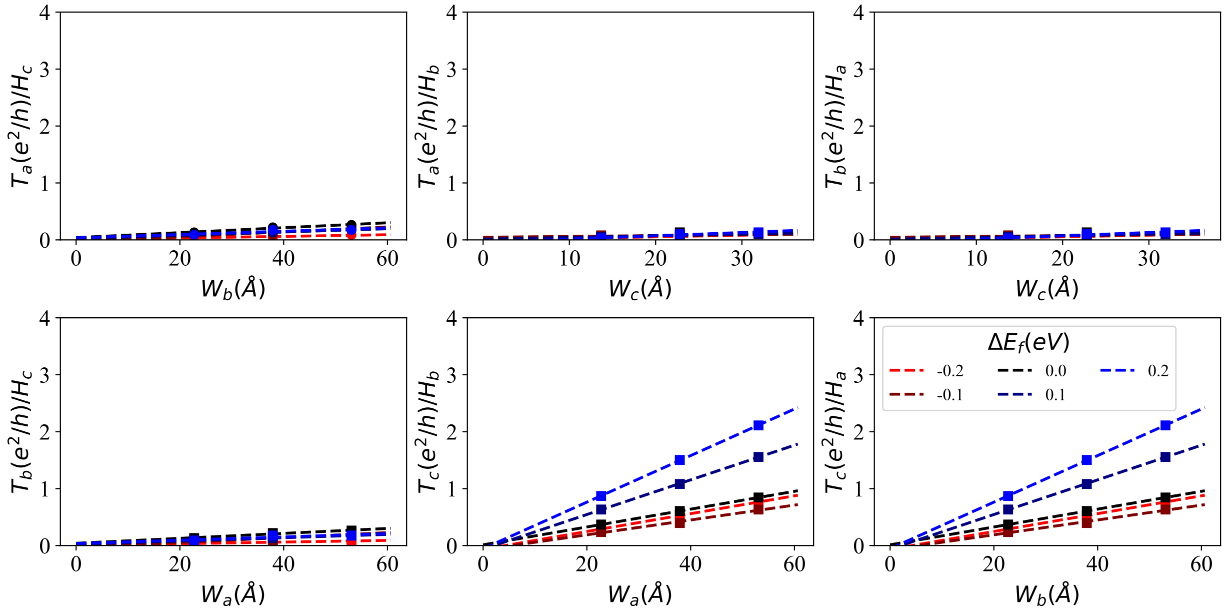

Compound: ZrB_2
Materials Project ID: 1472

Lattice (conventional cell):

Parameter	Value	Unit
a	3.1805	Å
b	3.1805	Å
c	3.5455	Å
α (alpha)	90.0000	°
β (beta)	90.0000	°
γ (gamma)	120.0000	°

Crystal structure (conventional cell):

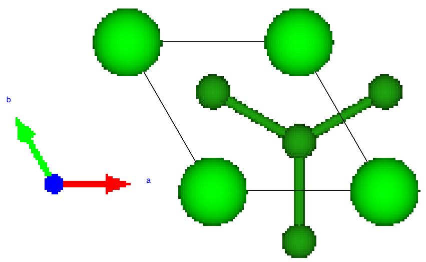

Nanowire Transmission:

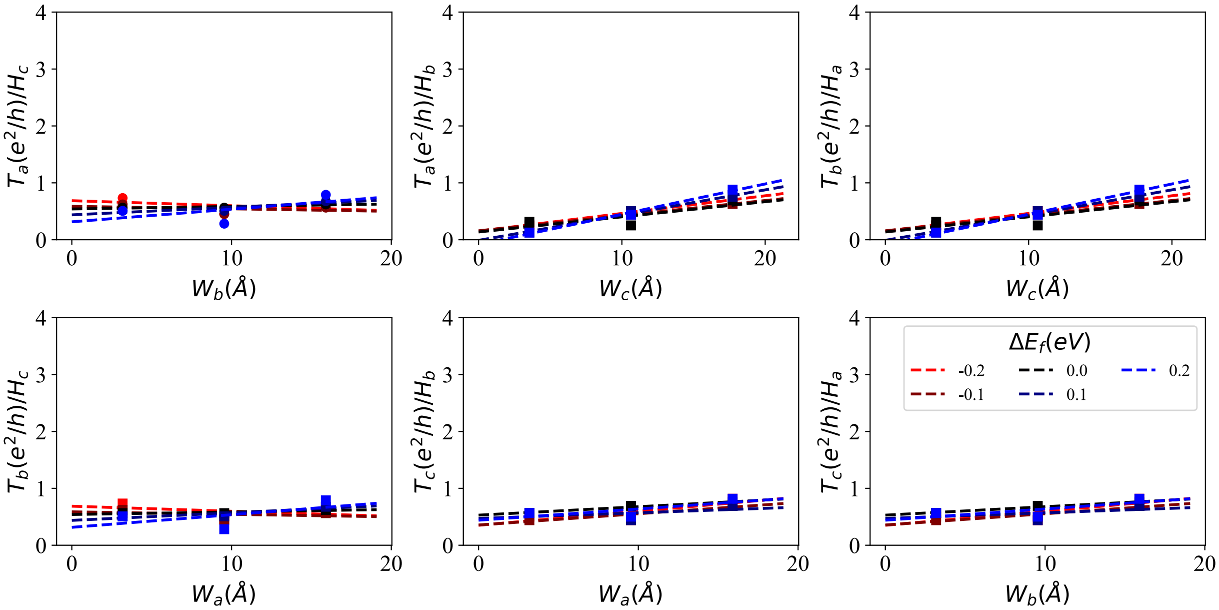

Compound: ZrGePt
Materials Project ID: 1095610

Lattice (conventional cell):

Parameter	Value	Unit
a	4.0158	Å
b	6.7301	Å
c	7.7560	Å
α (alpha)	90.0000	°
β (beta)	90.0000	°
γ (gamma)	90.0000	°

Crystal structure (conventional cell):

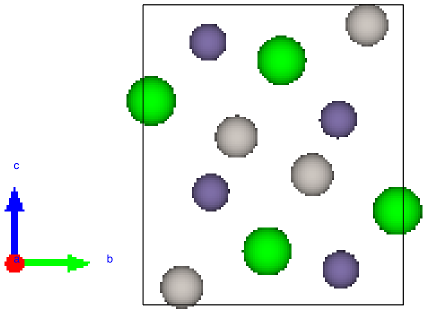

Nanowire Transmission:

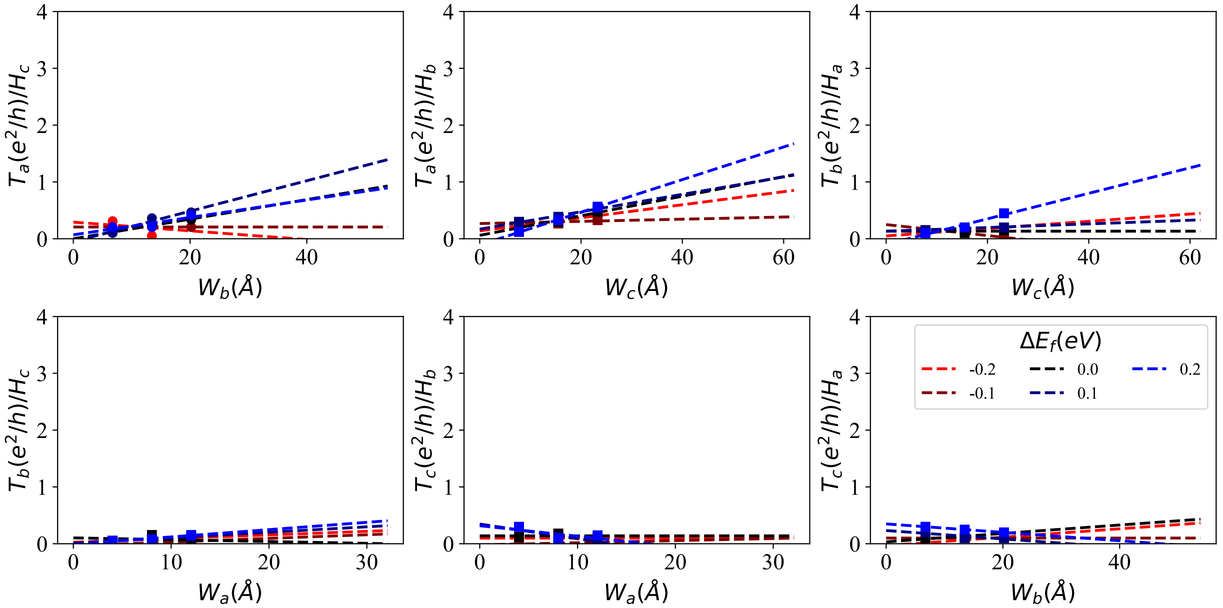

Compound: ZrSiPt
Materials Project ID: 972187

Lattice (conventional cell):

Parameter	Value	Unit
a	3.9323	Å
b	6.6654	Å
c	7.6233	Å
α (alpha)	90.0000	°
β (beta)	90.0000	°
γ (gamma)	90.0000	°

Crystal structure (conventional cell):

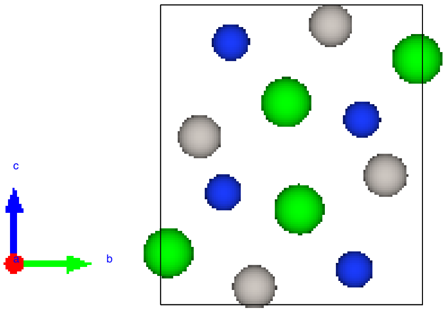

Nanowire Transmission:

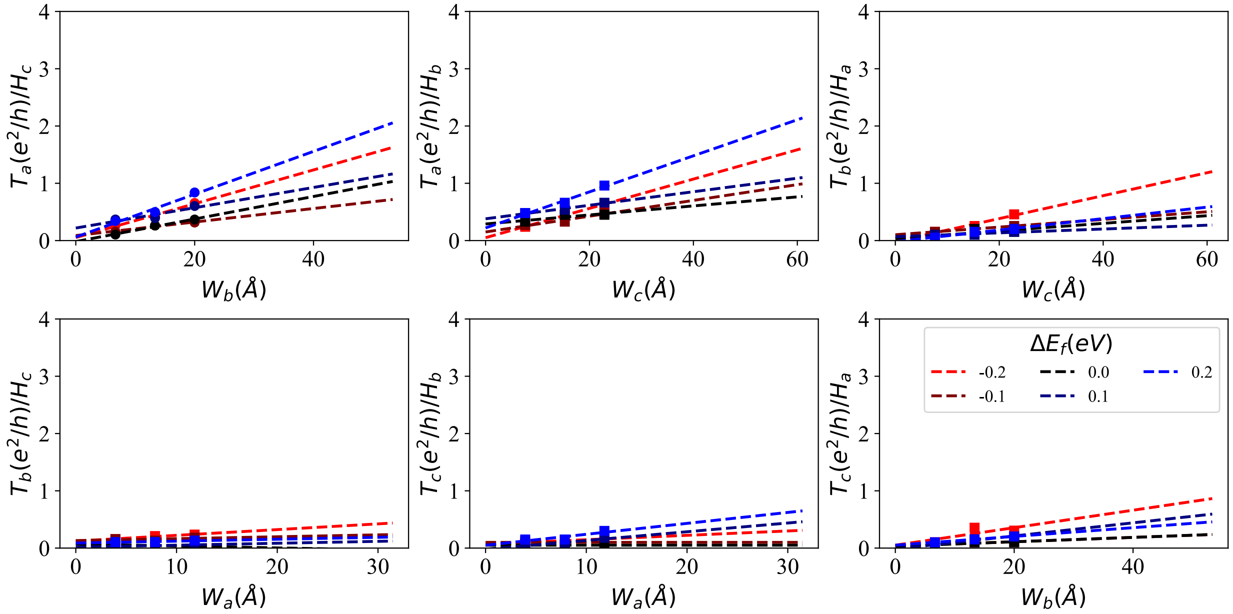

Compound: ZrSiS
Materials Project ID: 3938

Lattice (conventional cell):

Parameter	Value	Unit
a	3.6168	Å
b	3.6168	Å
c	8.6761	Å
α (alpha)	90.0000	°
β (beta)	90.0000	°
γ (gamma)	90.0000	°

Crystal structure (conventional cell):

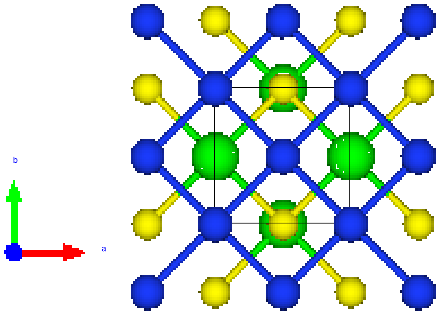

Nanowire Transmission:

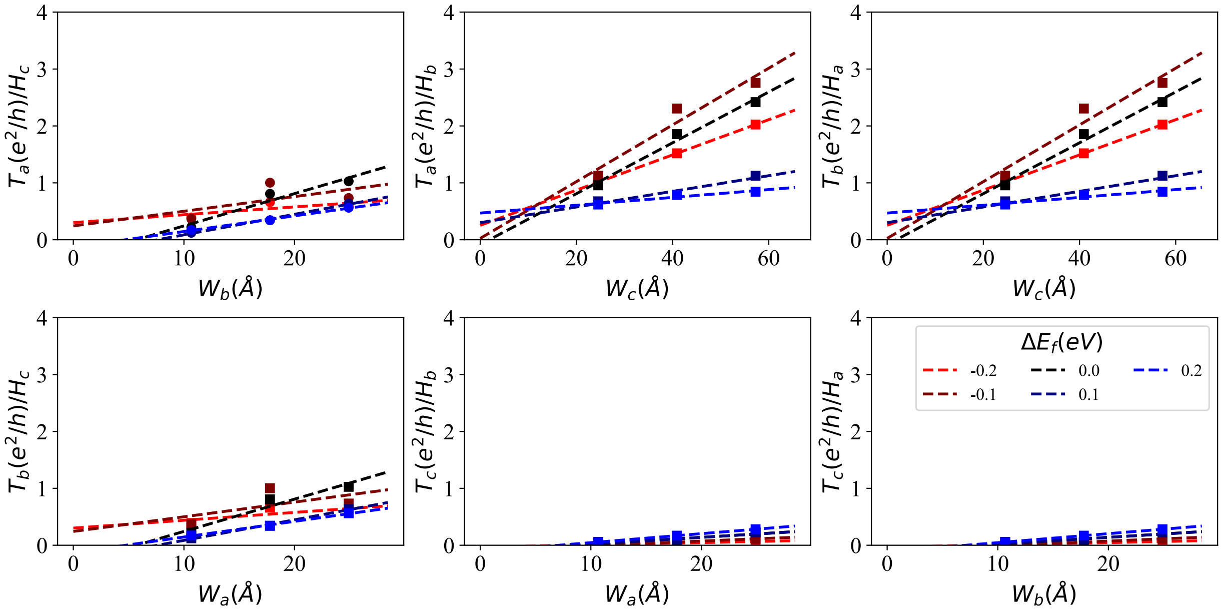

Compound: ZrSiSe
Materials Project ID: 81

Lattice (conventional cell):

Parameter	Value	Unit
a	3.6168	Å
b	3.6168	Å
c	8.6761	Å
α (alpha)	90.0000	°
β (beta)	90.0000	°
γ (gamma)	90.0000	°

Crystal structure (conventional cell):

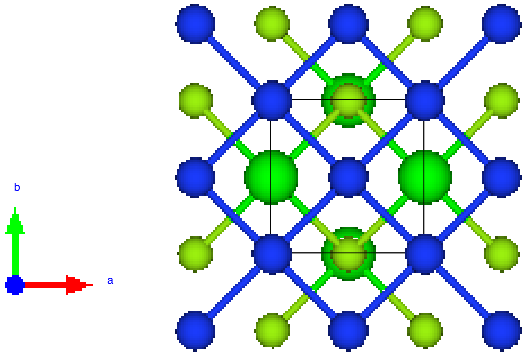

Nanowire Transmission:

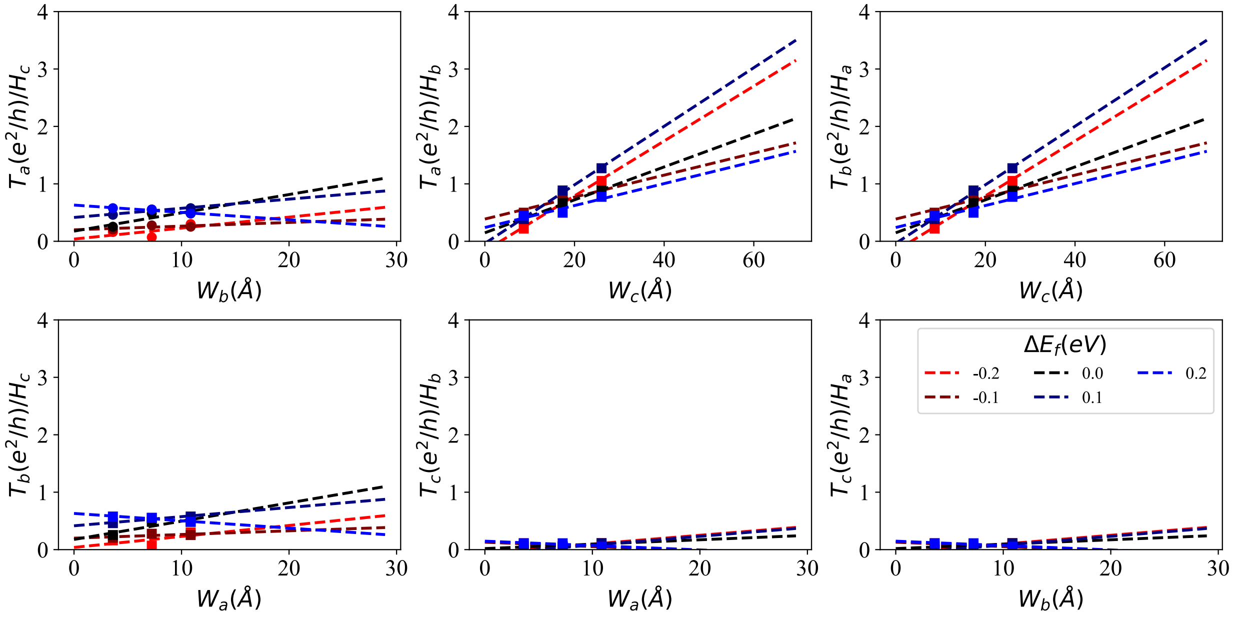

Compound: ZrTe
Materials Project ID: 1539

Lattice (conventional cell):

Parameter	Value	Unit
a	3.7994	Å
b	3.7994	Å
c	3.8986	Å
α (alpha)	90.0000	°
β (beta)	90.0000	°
γ (gamma)	120.0000	°

Crystal structure (conventional cell):

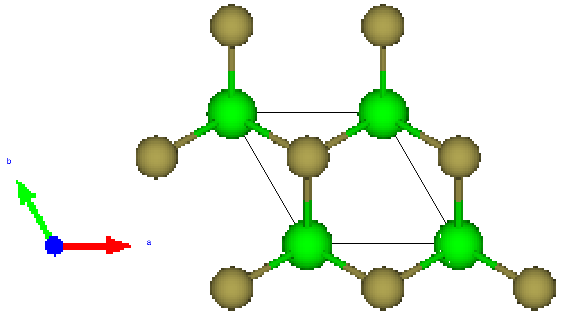

Nanowire Transmission:

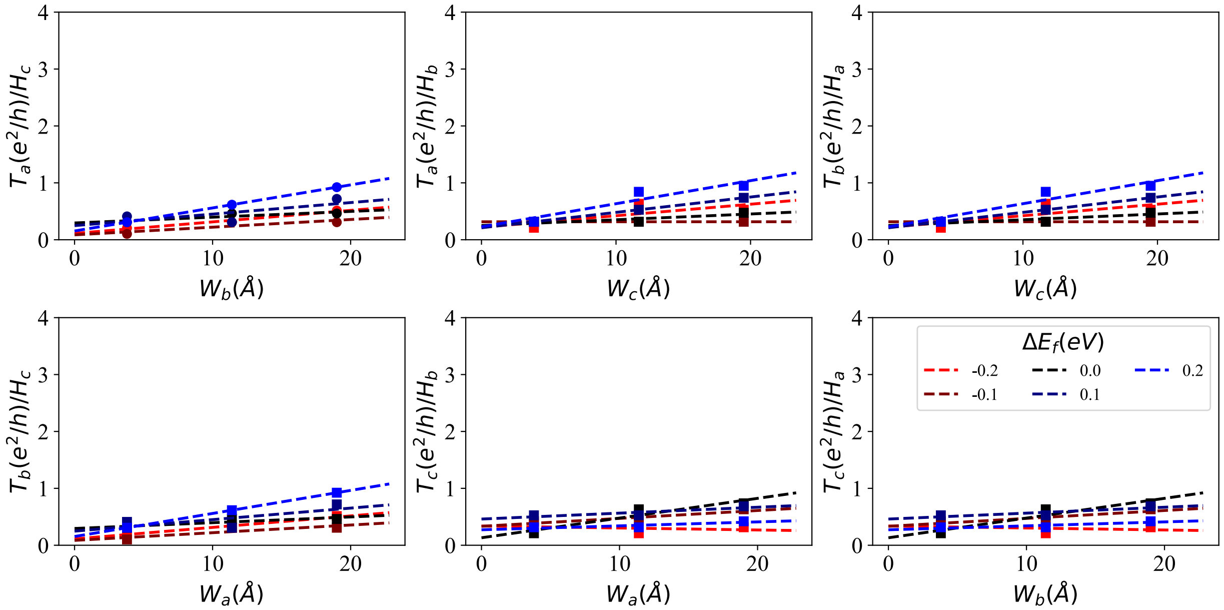

Supplement: Supplementary file 1 — Supporting File: advs73619‐sup‐0001‐SuppMat.pdf. [file ADVS-13-e20535-s001.pdf]
